# Association between the dietary inflammatory index, bowel habits, and systemic serum inflammatory markers: insights from NHANES (2005–2010)

**DOI:** 10.3389/fnut.2025.1543715

**Published:** 2025-03-26

**Authors:** Zeyang Zhou, Xiangyong Li, Mengya Xiong, Yuee He, Xinmeng Cheng, Jianbo Deng, Yanan Li, Xiaoyang Zhang, Zhengcao Zhang, Chenxi Zhou, Xiaodong Yang

**Affiliations:** ^1^Department of Gastrointestinal Surgery, The Second Affiliated Hospital of Soochow University, Soochow, China; ^2^Department of Operating Room, The Second Affiliated Hospital of Soochow University, Suzhou, China; ^3^National Center of Technology Innovation for Biopharmaceuticals, Suzhou, China

**Keywords:** DII, bowel habits, inflammation-related markers, NHANES, Bristol Stool Scale

## Abstract

**Objective:**

To examine the relationship between the Dietary Inflammatory Index (DII), abnormal bowel habits, and systemic serum inflammatory markers.

**Methods:**

Data from 9,880 participants in the National Health and Nutrition Examination Survey (NHANES) 2005–2010 were analyzed. The DII was calculated from two 24-h dietary recalls. Bowel habits were assessed using the Bristol Stool Form Scale, and systemic inflammatory markers included AAPR, IBI, NLR, LMR, PNLR, LCR, LA, and PLR. Statistical analyses were performed using R, Zstats, and EmpowerStats to evaluate associations.

**Results:**

Higher DII scores were positively associated with abnormal bowel habits, including constipation [*β* (95% CI): 0.11 (0.01–0.22)] and diarrhea [*β* (95% CI): 0.42 (0.32–0.53)], and with PNLR [*β* (95% CI): 0.01 (0.01–0.01)], PNLRQ4 [*β* (95% CI): 0.13 (0.05–0.20)], IBI [*β* (95% CI): 0.02 (0.01–0.02)], and IBIQ4 [*β* (95% CI): 0.33 (0.25–0.42)] (*p* < 0.05). Negative associations were found with AAPR [*β* (95% CI): −0.33 (−0.60 - −0.06)] and AAPRQ4 [*β* (95% CI): −0.18 (−0.34 - −0.01)], while no significant associations were observed with LA, LCR, or LMR. Subgroup analyses confirmed stable associations between DII and both chronic diarrhea and constipation across seven subgroups. Smoothed curve fitting revealed nonlinear relationships. A J-shaped association between DII and chronic constipation was identified in BMI and IBI subgroups. For BMI >30, the breakpoint (K) was 1.89, with ORs of 1.228 (95% CI: 1.097–1.375) below and 3.318 (95% CI: 1.531–7.191) above this point. In the IBI Q4 subgroup, the breakpoint was 1.96, with ORs of 1.145 (95% CI: 1.013–1.294) below and 5.794 (95% CI: 2.359–14.228) above. In the diarrhea group, a U-shaped association was observed in the AAPR Q4 population, with a breakpoint of −1.312 and ORs of 0.657 (95% CI: 0.478–0.901) below and 1.266 (95% CI: 1.057–1.518) above.

**Conclusion:**

Higher DII scores are linked to an increased risk of chronic constipation and diarrhea and are associated with systemic inflammatory markers and factors such as BMI.

## Introduction

Alterations in bowel habits, including constipation and diarrhea, are common gastrointestinal issues. Constipation is characterized by reduced bowel movement frequency and difficulty passing stool. It is typically mild and intermittent, often self-managed with over-the-counter fiber supplements and laxatives (1). Chronic diarrhea, on the other hand, is one of the leading reasons for referrals to gastroenterology clinics (2). Estimating its prevalence in Western populations is challenging due to demographic variations and definitional complexities. The etiology of gastrointestinal disorders is multifactorial, with contributing factors such as low fiber intake, dehydration, bacterial infections, and unhealthy behaviors like smoking, excessive alcohol consumption, and lack of physical activity. Diet plays a critical role in regulating the gut microbiome, providing an energy source and influencing inflammatory potential (3). Changes in gut barrier function and the gut microbiome are associated with various disease states (4). Disruption of the gut barrier and increased intestinal permeability are linked to numerous pathological conditions, including ulcerative colitis, colorectal cancer, and metabolic syndrome (5–7).

To assess the inflammatory potential of an individual’s diet, this study employed the Dietary Inflammatory Index (DII), an objective tool for quantifying diet-related inflammation by analyzing the effects of various foods and nutrients (8–10). Elevated DII scores are linked to an increased risk of multiple chronic diseases. However, the relationship between DII and bowel habits remains unclear. This study seeks to explore this relationship and identify potential dietary interventions to alleviate these common conditions.

To enhance our understanding of the correlation between diet, inflammation, and gut health, we conducted a cross-sectional analysis using the National Health and Nutrition Examination Survey (NHANES) database. The primary objective was to examine the potential link between DII and gut health, while accounting for confounding variables such as age, sex, and other dietary factors. Our findings aim to provide valuable insights for developing more effective dietary interventions for individuals with constipation and diarrhea.

## Materials and methods

### NHANES database

The NHANES database is a nationally representative cross-sectional survey designed to assess the health and nutritional status of the non-institutionalized population in the United States. Its sampling method involves multiple stages of probabilistic clustering, stratification, and further clustering. The study protocol was approved by the Ethics Review Board of the National Center for Health Statistics (NCHS), and all participants provided written informed consent (11). We used data from the 2005–2010 NHANES cycles, as these were the only cycles that included information on bowel habits and dietary intake. Bowel habit data were collected through the BHQ questionnaire, while demographic information, including age, sex, BMI, race, smoking status, and cancer history, was gathered via interviews. The NHANES data and samples are publicly available for further research.

### Bowel habits questionnaire

Participants were classified into three categories based on their responses to the bowel habits questionnaire: chronic diarrhea, chronic constipation, or normal bowel habits. The survey was conducted in the interview rooms of the Mobile Examination Center (MEC) using the Computer-Assisted Personal Interview (CAPI) system. To assess bowel habits, the Bristol Stool Form Scale (BSFS) was used, given its correlation with gut transit time (12, 13). Participants were shown a card with colored images depicting the seven types of stool on the BSFS (Types 1–7) and asked to identify the type most representative of their usual stool. Chronic constipation was defined as having BSFS Type 1 (hard, separate lumps resembling nuts) or Type 2 (sausage-shaped but lumpy) as the most common stool type. Chronic diarrhea was classified as BSFS Type 6 (fluffy pieces with irregular edges, mushy consistency) or Type 7 (watery, entirely lacking solid pieces). Participants whose stool types did not fit these categories were classified as having normal bowel habits.

### Calculation of dietary inflammatory index score

The Dietary Inflammatory Index (DII), developed by Shivappa, is a scoring system designed to evaluate the inflammatory potential of various nutrients through an extensive review of scientific literature. Its flexibility allows for scores to be generated even when data for all 45 parameters are not available, utilizing the portions of data that are present (8). It evaluates the pro-inflammatory and anti-inflammatory effects of diets by analyzing the intake of 45 different nutrients. To calculate the DII score, the consumption of each dietary component over the previous 24 h is assessed, and its Z-score is determined. The Z-score is then converted into a percentile, multiplied by 2, and subtracted by 1 to achieve a symmetric distribution. The individual scores are summed to derive the total DII score, which serves as an important tool for evaluating the inflammatory potential of a diet (8). In this study, dietary data were derived from two 24-h dietary recall interviews (14). The first interview was conducted in person at the Mobile Examination Center (MEC), and the second was conducted via telephone 3 to 10 days later. The average of both interviews was used to calculate the dietary data. In this study, 24 dietary components were selected to calculate the DII due to limitations in the types of components assessed by NHANES. These components included protein, carbohydrates, dietary fiber, various fats (total, saturated, monounsaturated, polyunsaturated), cholesterol, vitamins (A, C, E, *β*-carotene, thiamine, riboflavin, niacin, B12, B6, folate), and minerals (iron, magnesium, selenium, zinc), as well as alcohol and caffeine (3). DII scores range from −5.22 to 4.00, with quartiles established based on score distribution. The quartiles were defined as follows: Q1 (−5.22 ≤ DII < −1.25) representing the lowest dietary inflammation, Q2 (−1.25 ≤ DII < −0.07), Q3 (−0.07 ≤ DII < 0.91), and Q4 (0.91 ≤ DII < 4.00), representing the highest dietary inflammation. Participants were categorized into anti-inflammatory diet groups (DII < 0) and pro-inflammatory diet groups (DII > 0). Establishing quartiles based on DII score distribution allows for a more detailed analysis of the relationship between dietary inflammation and bowel habits. This approach facilitates the examination of potential dose–response relationships, enhancing the rigor of the findings. Overall, the use of DII scores and quartiles provides a comprehensive method for studying the link between diet and bowel habits.

### Calculation of serum systemic inflammatory markers

This study also recorded laboratory indicators, including Albumin, ALT, AST, Alkaline Phosphatase, Cholesterol, GST, Total Protein, Triglycerides, Globulin, WBC, Lymphocytes, Monocytes, Neutrophils, Eosinophils, RBC, Hb, PLT, and CRP. Systemic inflammatory markers in serum were calculated, and their clinical relevance has been demonstrated in various cancers, including gastrointestinal tumors, as indicators of systemic inflammation (15). These markers included AAPR (Albumin/Alkaline Phosphatase), Inflammatory Burden Index (IBI, CRP × Neutrophil Count/Lymphocyte Count), NLR (Neutrophil Count/Lymphocyte Count), LMR (Lymphocyte Count/Monocyte Count), PNLR (Platelets × Neutrophil Count/Lymphocyte Count), LCR (Lymphocyte Count/CRP), LA (Lymphocyte Count × Albumin), and PLR (Platelet Count/Lymphocyte Count) (15–19).

### Additional variables

The study collected demographic information, including age, gender, and race/ethnicity. Lifestyle factors such as smoking status, alcohol consumption, and personal history of malignancies were also recorded through self-reported questionnaires. Body Mass Index (BMI) was measured during the physical examination and categorized into three groups: <25 kg/m^2^, 25–30 kg/m^2^, and > 30 kg/m^2^.

### Statistical methods

Data organization and statistical analysis were conducted using Zstats software,^1^ EmpowerStats version 3.0,^2^ and R version 4.4.0 (2024-04-24). Categorical variables were presented as percentages with 95% confidence intervals (CIs), while continuous variables were reported as mean ± standard error to provide a comprehensive understanding of data distribution. To assess the relationship between the Dietary Inflammatory Index (DII) and bowel habits or inflammation-related biomarkers, we performed linear regression, adjusting for a range of potential confounders. Results were expressed as regression coefficients (*β*) with CIs for clearer interpretation. Subgroup and interaction analyses were conducted within fully adjusted logistic regression models, stratified by factors such as age, sex, BMI, race, AAPR, PNLR, LMR, LCR, and PLR. To further explore the relationship between DII and abnormal bowel habits, we first applied smoothed curve fitting to visualize potential nonlinear trends in the constipation and diarrhea groups. This method allowed for detecting complex patterns in the data that traditional linear models might overlook. Based on the results of the smoothed curves, we conducted threshold effect analysis using a two-piece linear regression model. The turning point (i.e., threshold) was identified through iterative trials, selecting the point along predefined intervals that maximized model likelihood. This sequential approach ensured a comprehensive examination of both nonlinear trends and threshold levels where significant changes in the relationship occurred.

### Inclusion and exclusion criteria

**Figure 1:**
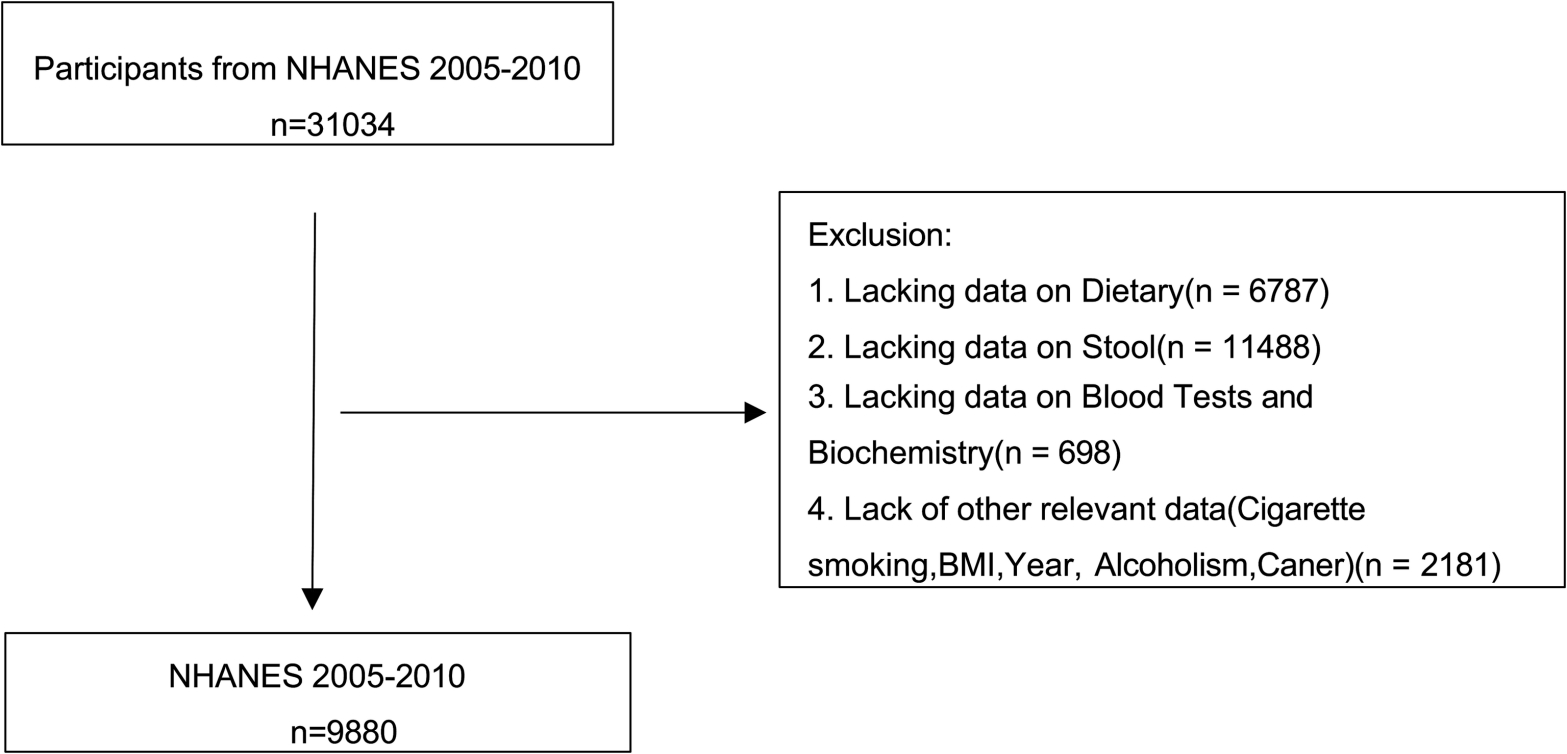
Study design flowchart.

Participants were excluded based on the following criteria: (1) missing data on the bowel habits questionnaire or the Dietary Inflammatory Index (DII), and (2) incomplete covariate information, including age, race, gender, BMI, smoking status, alcohol consumption, cancer history, or laboratory test results (details of the inclusion/exclusion criteria are provided in Figure 1). Ultimately, 9,880 participants were included in the study.

## Result

### Demographic characteristics of participants

Table 1 compares the characteristics of participants in the pro-inflammatory diet group (*N* = 4,779) and the anti-inflammatory diet group (*N* = 5,101), focusing on demographic, lifestyle, health-related factors, and bowel habits. Statistically significant differences (*p* < 0.05) were observed between the two groups for BMI, Albumin, ALT, AST, Alkaline Phosphatase, Globulin, WBC, Neutrophils, RBC, Hb, PLT, CRP, AAPR, inflammatory markers, PNLR, LA, LMR, LCR, Gender, Race, Bowel Habits, and Smoking. Conversely, no statistically significant differences (*p* > 0.05) were noted for Year, Cholesterol, GST, Total Protein, Triglycerides, Lymphocytes, Monocytes, Eosinophils, NLR, PLR, or Cancer.

**Table 1:** Weighted baseline characteristics of participants.

Variables	Total (*n* = 9,880)	Anti-inflammatory (*n* = 5,101)	Pro-inflammatory (*n* = 4,779)	Statistic	*P*
Age (years)	48.08 (34.67, 61.83)	47.58 (34.75, 61.33)	48.67 (34.58, 62.33)	Z = −1.35	0.176
BMI (kg/m^2^)	28.09 (24.54, 32.39)	27.73 (24.39, 32.08)	28.44 (24.70, 32.78)	Z = −4.79	<0.001
Gender, *n* (%)				χ^2^ = 38.63	<0.001
Female	4,773 (48.31)	2,310 (45.29)	2,463 (51.54)		
Male	5,107 (51.69)	2,791 (54.71)	2,316 (48.46)		
Race, *n* (%)				χ^2^ = 46.25	<0.001
Mexican American	1772 (17.94)	973 (19.07)	799 (16.72)		
Non-Hispanic Black	1854 (18.77)	842 (16.51)	1,012 (21.18)		
Non-Hispanic White	5,085 (51.47)	2,695 (52.83)	2,390 (50.01)		
Other Hispanic	816 (8.26)	394 (7.72)	422 (8.83)		
Other Race	353 (3.57)	197 (3.86)	156 (3.26)		
Bowel Habits, *n* (%)				χ^2^ = 6.62	0.037
Constipation	690 (6.98)	327 (6.41)	363 (7.60)		
Diarrhea	725 (7.34)	362 (7.10)	363 (7.60)		
Normal	8,465 (85.68)	4,412 (86.49)	4,053 (84.81)		
Caner, *n* (%)				χ^2^ = 0.01	0.936
No	9,028 (91.38)	4,660 (91.35)	4,368 (91.40)		
Yes	852 (8.62)	441 (8.65)	411 (8.60)		
Smoking, *n* (%)				χ^2^ = 20.60	<0.001
Current smoker	1,493 (15.11)	742 (14.55)	751 (15.71)		
Former smoker	3,660 (37.04)	1806 (35.40)	1854 (38.79)		
Non-smoker	4,727 (47.84)	2,553 (50.05)	2,174 (45.49)		
Albumin (g/dL)	4.20 (4.00, 4.50)	4.30 (4.00, 4.50)	4.20 (4.00, 4.40)	Z = −6.68	<0.001
ALT (U/L)	22.00 (17.00, 29.00)	22.00 (17.00, 30.00)	21.00 (16.00, 28.00)	Z = −5.45	<0.001
AST (U/L)	23.00 (20.00, 28.00)	24.00 (20.00, 28.00)	23.00 (20.00, 28.00)	Z = −5.33	<0.001
ALP (U/L)	66.00 (54.00, 81.00)	66.00 (54.00, 80.00)	67.00 (54.00, 82.00)	Z = −2.70	0.007
Cholesterol (g/L)	194.00 (168.75, 224.00)	195.00 (170.00, 224.00)	194.00 (167.00, 224.00)	Z = −1.14	0.252
GST (g/L)	21.00 (15.00, 32.00)	21.00 (15.00, 32.00)	21.00 (15.00, 33.00)	Z = −0.97	0.334
Total-protein (g/L)	7.10 (6.80, 7.40)	7.10 (6.80, 7.40)	7.10 (6.80, 7.40)	Z = −0.55	0.585
Triglyceride (g/L)	124.00 (82.00, 194.00)	124.00 (82.00, 195.00)	124.00 (83.00, 193.00)	Z = −0.12	0.906
Globulin, M (g/L)	2.90 (2.60, 3.20)	2.90 (2.60, 3.20)	2.90 (2.60, 3.20)	Z = −3.83	<0.001
WBC (10^9^/L)	6.90 (5.80, 8.40)	6.90 (5.70, 8.40)	7.10 (5.80, 8.40)	Z = −2.82	0.005
Lymphocyte (10^9^/L)	2.10 (1.70, 2.50)	2.00 (1.70, 2.50)	2.10 (1.70, 2.50)	Z = −1.59	0.113
Monocyte (10^9^/L)	0.50 (0.40, 0.60)	0.50 (0.40, 0.60)	0.50 (0.40, 0.60)	Z = −0.06	0.950
Neutrophil (10^9^/L)	4.00 (3.10, 5.20)	4.00 (3.10, 5.20)	4.10 (3.20, 5.20)	Z = −2.51	0.012
Eosinophil (10^9^/L)	0.20 (0.10, 0.30)	0.20 (0.10, 0.30)	0.20 (0.10, 0.30)	Z = −1.88	0.060
RBC (10^12^/L)	4.69 (4.35, 5.04)	4.72 (4.37, 5.07)	4.66 (4.32, 5.01)	Z = −4.93	<0.001
Hb (g/dL)	14.40 (13.30, 15.40)	14.50 (13.50, 15.50)	14.30 (13.20, 15.30)	Z = −6.95	<0.001
PLT (10^9^/L)	253.00 (214.00, 298.00)	250.00 (211.00, 295.00)	256.00 (217.00, 302.00)	Z = −4.99	<0.001
CRP (g/L)	0.20 (0.08, 0.46)	0.18 (0.07, 0.42)	0.21 (0.09, 0.50)	Z = −6.13	<0.001
AAPR, M (Q₁, Q₃)	0.64 (0.52, 0.79)	0.65 (0.53, 0.80)	0.63 (0.51, 0.78)	Z = −3.88	<0.001
IBI, M (Q₁, Q₃)	0.38 (0.14, 1.00)	0.35 (0.13, 0.90)	0.41 (0.16, 1.09)	Z = −6.02	<0.001
NLR, M (Q₁, Q₃)	1.96 (1.47, 2.60)	1.95 (1.48, 2.57)	1.96 (1.47, 2.63)	Z = −0.70	0.484
PNLR, M (Q₁, Q₃)	493.06 (354.76, 693.58)	483.56 (347.40, 678.90)	504.15 (361.63, 715.95)	Z = −3.72	<0.001
LA, M (Q₁, Q₃)	8.61 (6.97, 10.80)	8.58 (6.90, 10.58)	8.80 (6.97, 10.92)	Z = −3.02	0.003
LMR, M (Q₁, Q₃)	4.00 (3.11, 5.00)	4.00 (3.00, 5.00)	4.00 (3.14, 5.00)	Z = −3.80	<0.001
LCR, M (Q₁, Q₃)	10.45 (4.47, 26.00)	10.95 (4.75, 27.78)	10.00 (4.20, 24.37)	Z = −4.49	<0.001
PLR, M (Q₁, Q₃)	122.73 (97.27, 156.11)	122.00 (97.14, 154.67)	123.46 (97.37, 157.38)	Z = −1.26	0.209

Z: Mann–Whitney test, χ^2^: Chi-square test. M: median, Q₁: 1st quartile, Q₃: 3st quartile.

### The relationship between DII and bowel habits

Table 2 illustrates the associations between Dietary Inflammatory Index (DII) scores and bowel habits, AAPR, IBI, PNLR, LA, LCR, and LMR. Across the crude model, partially adjusted model, and fully adjusted model, DII scores showed significant positive associations with abnormal bowel habits, PNLR, and IBI, negative associations with AAPR, and no statistically significant relationships with LA, LCR, or LMR. In the fully adjusted model, a 1-unit increase in DII scores was associated with an increase in the incidence of chronic constipation by 0.11 units compared to the normal control group [*β* (95% CI): 0.11 (0.01–0.22)], and an increase in the incidence of chronic diarrhea by 0.42 units compared to the normal control group [*β* (95% CI): 0.42 (0.32–0.53)], both statistically significant (*p* < 0.05). Additionally, for each 1-unit increase in DII scores, IBI increased by 0.02 units [*β* (95% CI): 0.02 (0.01–0.02)], PNLR increased by 0.01 units [*β* (95% CI): 0.01 (0.01–0.01)], and AAPR decreased by 0.33 units [*β* (95% CI): −0.33 (−0.60–−0.06)], all statistically significant (*p* < 0.05). Quartile analysis further revealed significant trends. Compared to Q1, AAPR in Q4 decreased by 0.18 units [*β* (95% CI): −0.18 (−0.34–−0.01)], IBI in Q4 increased by 0.33 units [*β* (95% CI): 0.33 (0.25–0.42)], and PNLR in Q4 increased by 0.13 units [*β* (95% CI): 0.13 (0.05–0.20)], all statistically significant (*p* < 0.05).

**Table 2:** Association between DII, bowel habits, and serum inflammatory markers.

Variables	Model1		Model2		Model3	
	β (95%CI)	*P*	β (95%CI)	*P*	β (95%CI)	*P*
Bowel habits						
Normal	0.00 (Reference)		0.00 (Reference)		0.00 (Reference)	
Constipation	0.16 (0.05 ~ 0.27)	**0.004**	0.16 (0.06 ~ 0.27)	**0.003**	0.11 (0.01 ~ 0.22)	**0.036**
Diarrhea	0.44 (0.33 ~ 0.55)	**<0.001**	0.40 (0.29 ~ 0.51)	**<0.001**	0.42 (0.32 ~ 0.53)	**<0.001**
AAPR	−0.68 (−0.80 ~ −0.55)	**<0.001**	−0.42 (−0.70 ~ −0.15)	**0.002**	−0.33 (−0.60 ~ −0.06)	**0.016**
AAPR quantile						
Q1 (0.12–0.52)	0.00 (Reference)		0.00 (Reference)		0.00 (Reference)	
Q2 (0.52–0.67)	−0.21 (−0.29 ~ −0.13)	**<0.001**	−0.15 (−0.24 ~ −0.06)	**<0.001**	−0.15 (−0.24 ~ −0.06)	**<0.001**
Q3 (0.67–0.89)	−0.29 (−0.37 ~ −0.22)	**<0.001**	−0.18 (−0.28 ~ −0.07)	**0.002**	−0.15 (−0.26 ~ −0.04)	**0.006**
Q4 (0.79–3.75)	−0.42 (−0.50 ~ −0.34)	**<0.001**	−0.22 (−0.38 ~ −0.05)	**0.011**	−0.18 (−0.34 ~ −0.01)	**0.035**
IBI	0.03 (0.02 ~ 0.04)	**<0.001**	0.03 (0.02 ~ 0.04)	**<0.001**	0.02 (0.01 ~ 0.02)	**<0.001**
IBI quantile						
Q1 (0.01–0.14)	0.00 (Reference)		0.00 (Reference)		0.00 (Reference)	
Q2 (0.14–0.38)	0.18 (0.10 ~ 0.26)	**<0.001**	0.20 (0.12 ~ 0.28)	**<0.001**	0.16 (0.09 ~ 0.24)	**<0.001**
Q3 (0.38–1.00)	0.38 (0.30 ~ 0.46)	**<0.001**	0.39 (0.31 ~ 0.47)	**<0.001**	0.29 (0.21 ~ 0.37)	**<0.001**
Q4 (1.00–129.41)	0.54 (0.46 ~ 0.62)	**<0.001**	0.54 (0.46 ~ 0.62)	**<0.001**	0.33 (0.25 ~ 0.42)	**<0.001**
PNLR	0.01 (0.01 ~ 0.01)	**0.002**	0.01 (0.01 ~ 0.01)	**0.002**	0.01 (0.01 ~ 0.01)	**0.046**
PNLR quantile						
Q1 (11.23–354.76)	0.00 (Reference)		0.00 (Reference)		0.00 (Reference)	
Q2 (354.76–493.06)	0.10 (0.02 ~ 0.18)	**0.017**	0.10 (0.03 ~ 0.18)	**0.009**	0.08 (0.01 ~ 0.15)	**0.049**
Q3 (493.06–693.58)	0.03 (−0.05 ~ 0.11)	0.400	0.04 (−0.03 ~ 0.12)	0.273	0.03 (−0.05 ~ 0.11)	0.465
Q4 (693.58–28397.28)	0.19 (0.11 ~ 0.27)	**<0.001**	0.19 (0.11 ~ 0.27)	**<0.001**	0.13 (0.05 ~ 0.20)	**0.001**
LA	0.00 (−0.00 ~ 0.01)	0.526	0.00 (−0.00 ~ 0.01)	0.404	0.00 (−0.00 ~ 0.01)	0.470
LCR	−0.01 (−0.99 ~ −0.01)	**<0.001**	−0.01 (−0.99 ~ −0.01)	**0.001**	−0.00 (−0.00 ~ 0.00)	0.484
LMR	0.02 (0.01 ~ 0.03)	**0.042**	0.00 (−0.01 ~ 0.02)	0.835	0.00 (−0.01 ~ 0.02)	0.673

CI: Confidence Interval. Model1: Crude. Model2: Adjust: gender, year, race. Model3: Adjust: gender, year, race, BMI, caner, ALT, AST, cholesterol, GST, triglyceride, eosinophil, smoking. Bold values indicate statistical significance (*p* < 0.05).

### Stratified analysis

**Figure 2:**
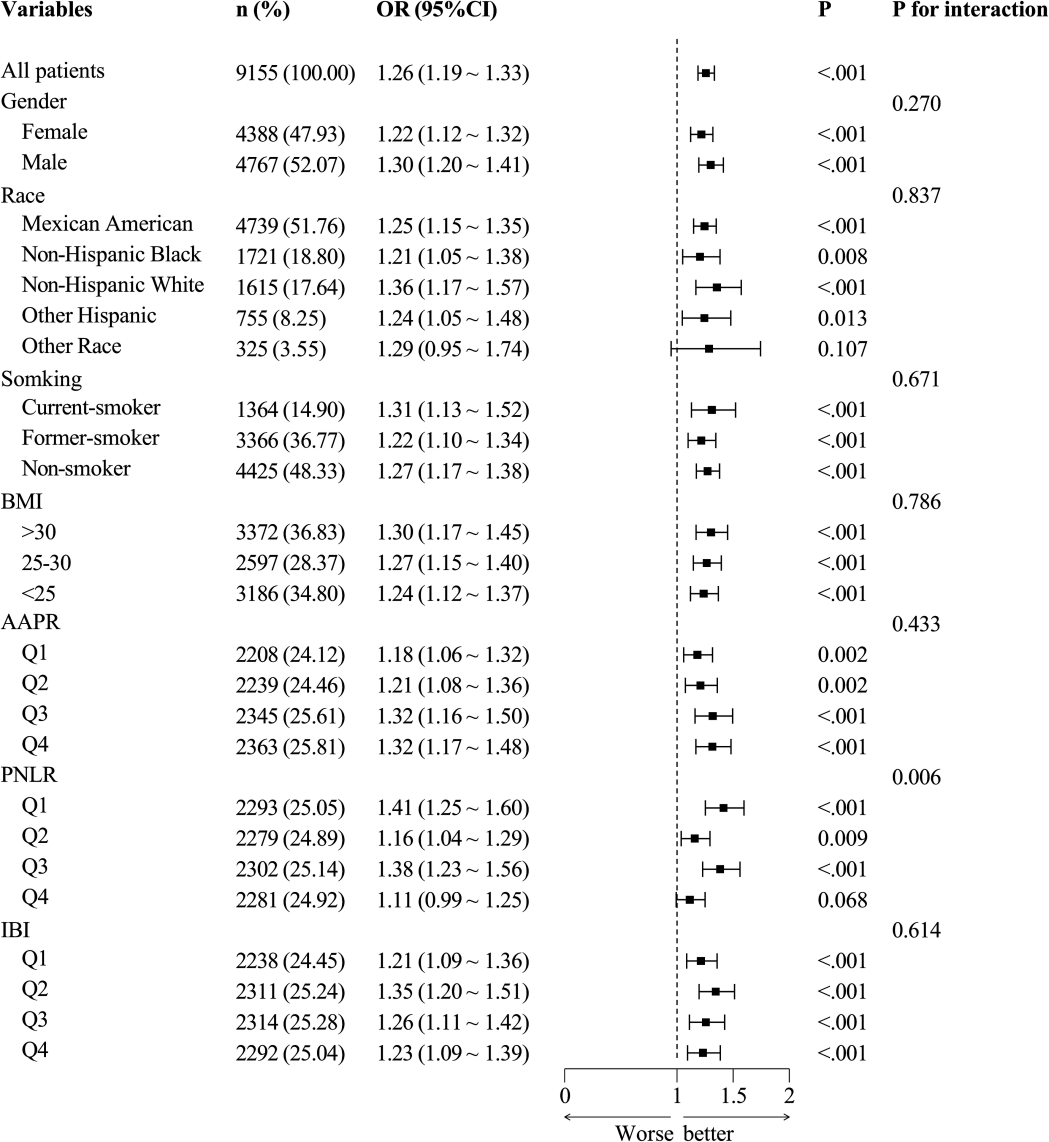
Forest plot of subgroup analysis for the constipation group.

**Figure 3:**
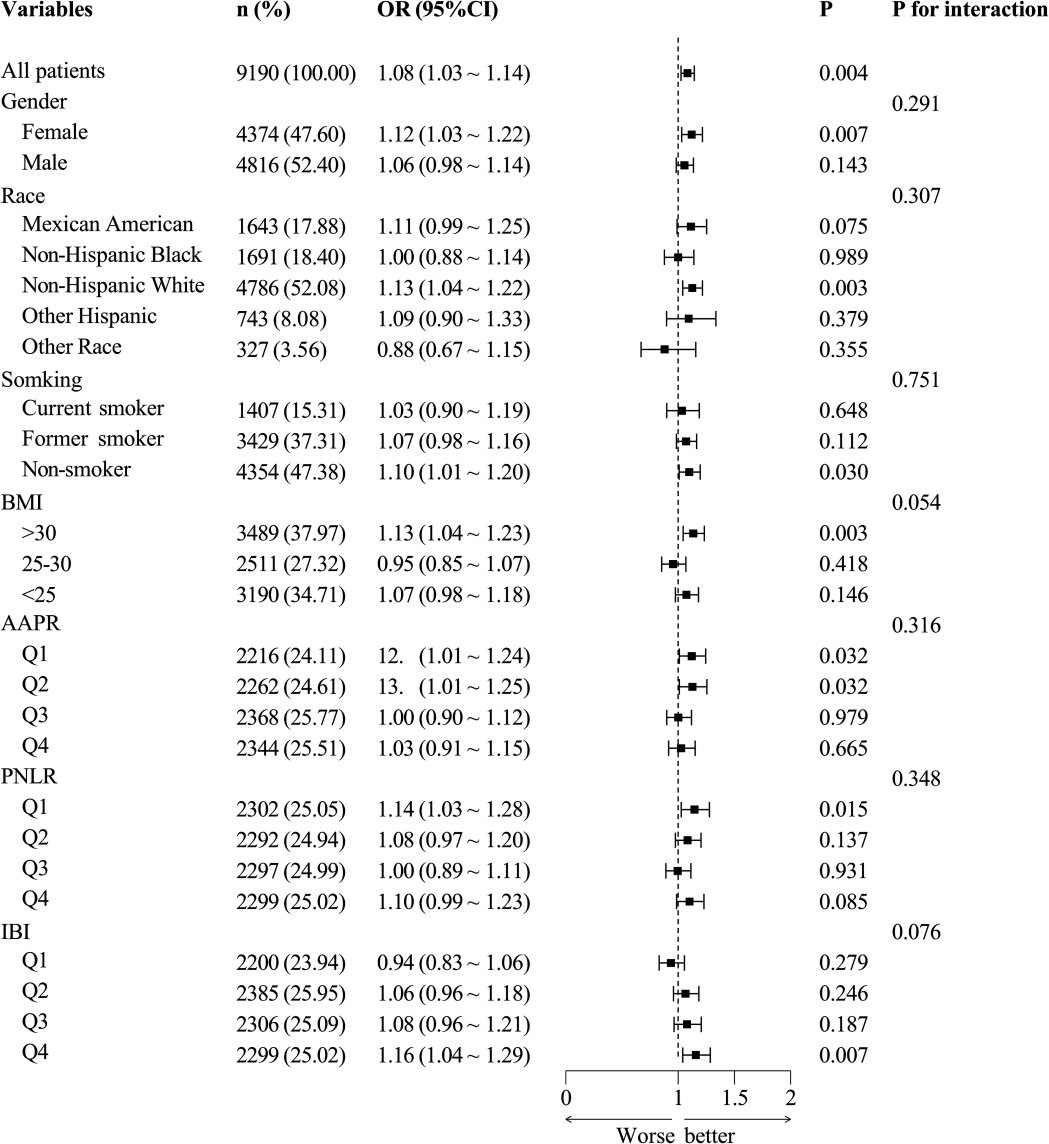
Forest plot for subgroup analysis of the diarrhea group.

Using the normal bowel movement population (*n* = 8,465) as the control, participants were categorized into a constipation group (*n* = 9,155, Figure 2) and a diarrhea group (*n* = 9,190, Figure 3). Subgroup analysis and interaction testing revealed no significant impact of any covariates on the association between DII and chronic diarrhea (interaction *p* > 0.05 for all covariates). In the constipation group, differences were observed among PNLR groups (*p* = 0.006), though the overall trend across groups remained consistent. Other covariates showed no significant influence on the relationship between DII and chronic constipation. These findings indicate that the association between DII and both chronic diarrhea and constipation was consistent across all seven subgroups, demonstrating stable and robust results.

### Smooth curve fitting

**Figure 4:**
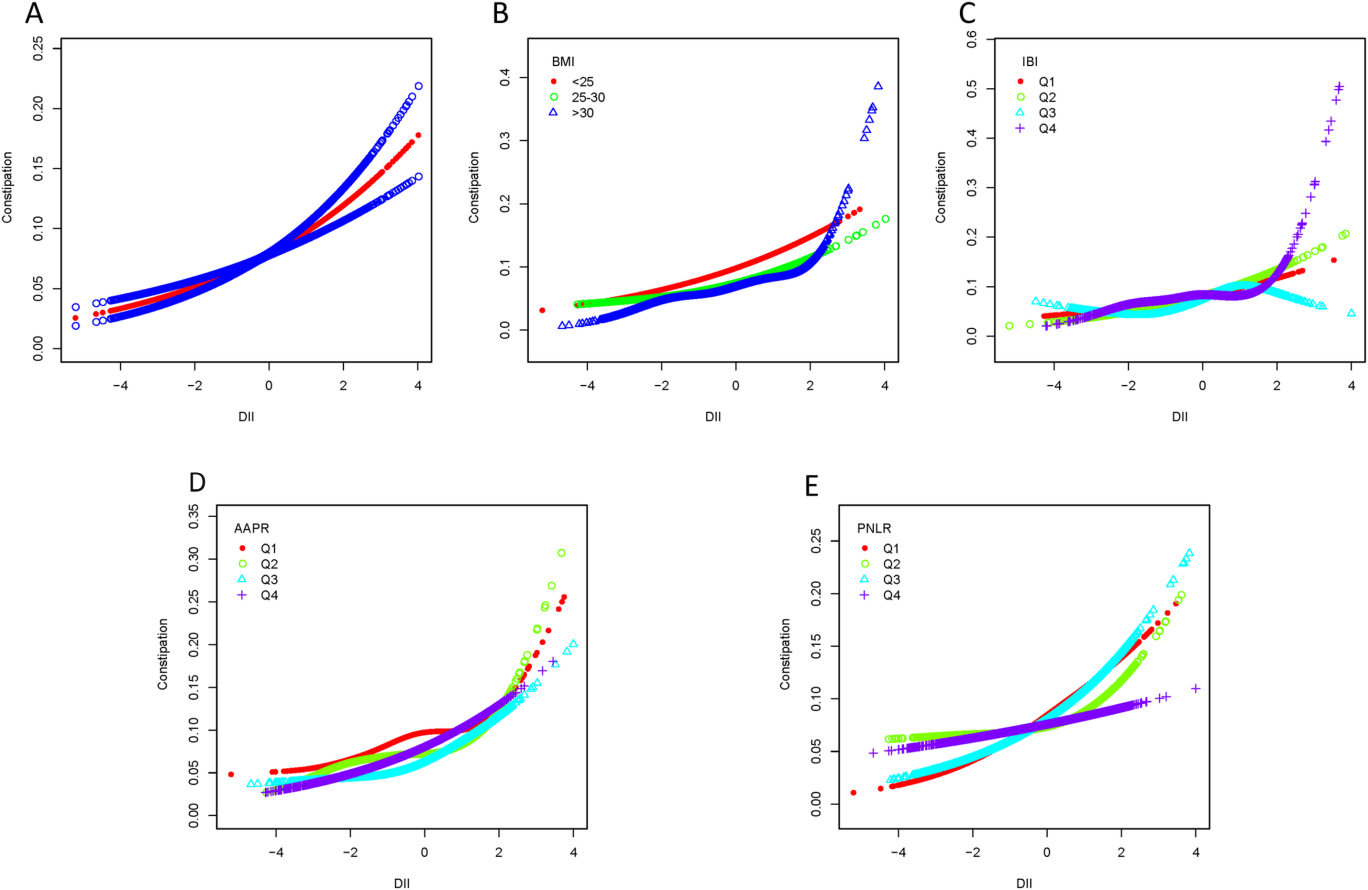
Smooth curve fitting of the constipation group. **(A)** Smooth curve fitting revealed a nonlinear association between DII scores and constipation. **(B)** Smooth curve fitting stratified by BMI. **(C)** Smooth curve fitting stratified by IBI. **(D)** Smooth curve fitting stratified by AAPR. **(E)** Smooth curve fitting stratified by PNLR.

**Figure 5:**
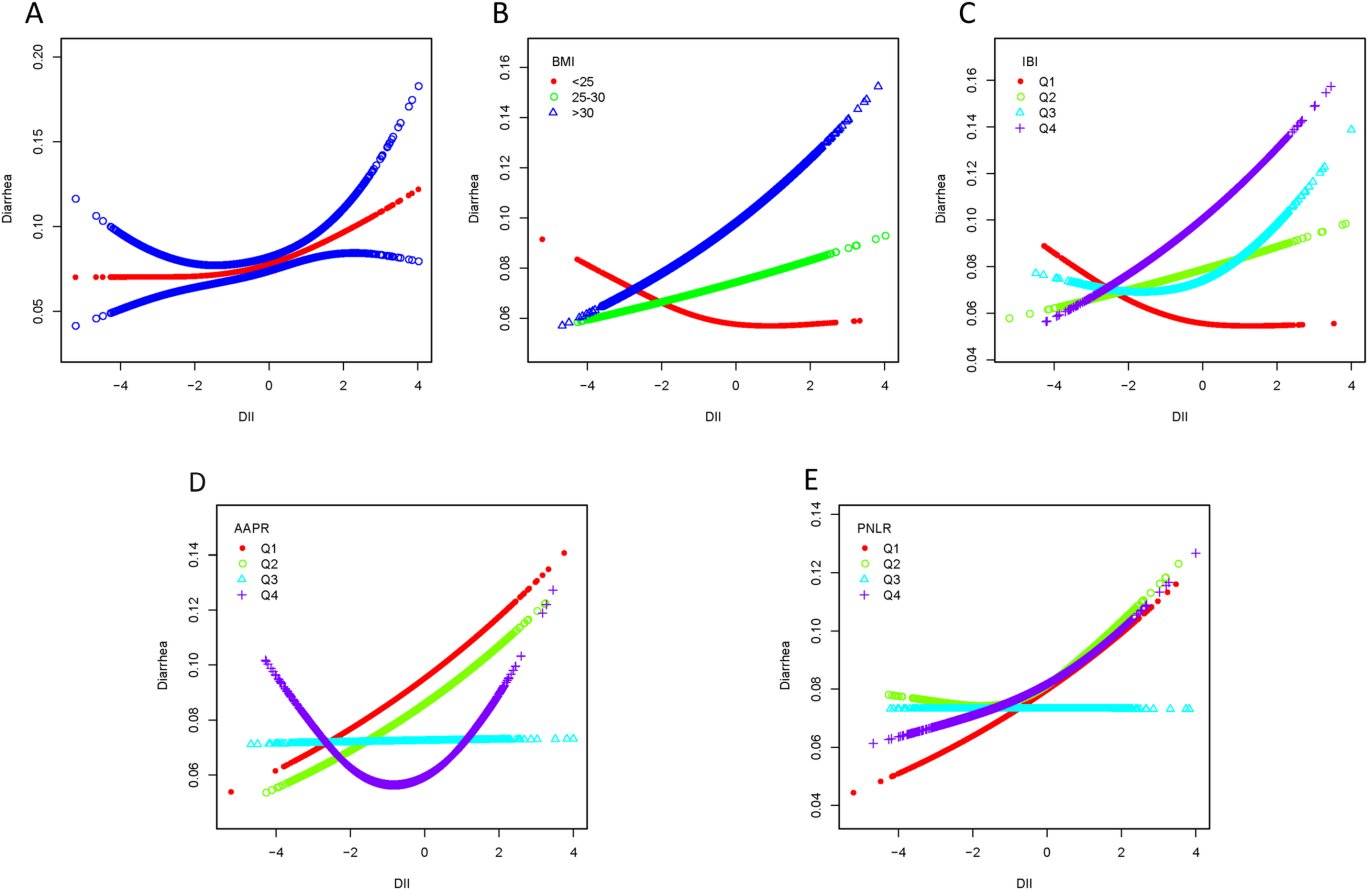
Smooth curve fitting of the diarrhea group. **(A)** Smooth curve fitting revealed a nonlinear association between DII scores and diarrhea. **(B)** Smooth curve fitting stratified by BMI. **(C)** Smooth curve fitting stratified by IBI. **(D)** Smooth curve fitting stratified by AAPR. **(E)** Smooth curve fitting stratified by PNLR.

Smooth curve fitting revealed a nonlinear association between DII scores and chronic constipation or diarrhea (Figures 4A, 5A). In the constipation group, the breakpoint (K) was determined to be 1.859. Below this point, each 1-unit increase in DII was associated with a 22.3% increase in the risk of chronic constipation, while above it, the risk increased by 125.9% (Table 3). Subsequently, we performed smooth curve fitting stratified by BMI, IBI, AAPR, and PNLR within the constipation cohort (Figures 4B–E). Smooth curve fitting stratified by BMI and IBI showed a J-shaped association between DII and chronic constipation (Figures 4B,C). Using a recursive algorithm, the breakpoint (K) was calculated as 1.89 for individuals with BMI >30. Below this point, a 1-unit increase in DII was linked to a 22.8% increase in constipation risk, while above it, the risk rose by 231.8% (Table 3). Among individuals in IBIQ4, the breakpoint (K) was 1.96. Below this point, a 1-unit increase in DII corresponded to a 14.5% increase in constipation risk, while above it, the risk increased by 479.4% (Table 3). In the diarrhea group, the breakpoint (K) was −0.118. Below this value, a 1-unit increase in DII reduced the risk of chronic diarrhea by 1.8%, though this was not statistically significant; above it, the risk increased by 19.8% (Table 4). Similarly, smooth curve fitting stratified by BMI, IBI, AAPR, and PNLR were constructed in the diarrhea cohort (Figures 5B–E). Notably, smooth curve fitting stratified by AAPR showed a U-shaped association between DII and chronic diarrhea (Figure 5D). Recursive calculations in the AAPRQ4 group identified a breakpoint (K) of −1.312. To the left of this point, a 1-unit increase in DII reduced the risk of chronic diarrhea by 34.3%, whereas to the right, the risk increased by 26.6% per unit increase (Table 4).

**Table 3:** Threshold effect analysis of DII and constipation using two-precise linear regression.

Constipation	Adjusted OR (95% CI)	*p*
Fitting by a standard linear model	1.250 (1.179, 1.325)	< 0.0001
Fitting by two precise linear model	1.859	
Inflection point		
<1.859	1.223 (1.150, 1.300)	<0.0001
>1.859	2.259 (1.317, 3.873)	0.0031
Log-likelihood ratio	0.040	
BMI > 30		
Fitting by a standard linear model	1.286 (1.154, 1.432)	<0.0001
Fitting by two precise linear model		
Inflection point	1.89	
<1.89	1.228 (1.097, 1.375)	0.0005
>1.89	3.318 (1.531, 7.191)	0.0014
Log-likelihood ratio	0.024	
IBI Q4		
Fitting by a standard linear model	1.228 (1.091, 1.383)	0.0007
Fitting by two precise linear model		
Inflection point	1.96	
<1.96	1.145 (1.013, 1.294)	0.0307
>1.96	5.794 (2.359, 14.228)	0.0001
Log-likelihood ratio	<0.001	

Adjust: Gender, year, race, caner, ALT, AST, cholesterol, GST, triglyceride, eosinophil, smoking.

**Table 4:** Threshold effect analysis of DII and diarrhea using two-precise linear regression.

Diarrhea	Adjusted OR (95% CI)	*p*
Fitting by a standard linear model	1.078 (1.020, 1.139)	0.0079
Fitting by two precise linear model		
Inflection point	−0.118	
<−0.118	0.982 (0.896, 1.098)	0.8739
> − 0.118	1.198 (1.060, 1.355)	0.0038
Log-likelihood ratio	0.060	
AAPR Q4		
Fitting by a standard linear model	1.033 (0.918, 1.164)	0.5873
Fitting by two precise linear model		
Inflection point	−1.312	
<−1.312	0.657 (0.478, 0.901)	0.0093
> − 1.312	1.266 (1.057, 1.518)	0.0106
Log-likelihood ratio	0.004	

Adjust: gender, year, race, caner, ALT, AST, cholesterol, GST, triglyceride, eosinophil, smoking.

## Discussion

This study demonstrated significant associations between higher Dietary Inflammatory Index (DII) scores, abnormal bowel habits, and systemic inflammatory markers under the fully adjusted model. Higher DII scores were independently associated with increased risks of both chronic constipation and diarrhea, with *β* coefficients of 0.11 (95% CI: 0.01–0.22) and 0.42 (95% CI: 0.32–0.53), respectively. Positive associations were also observed between DII and inflammatory markers, including PNLR (*β* = 0.01, 95% CI: 0.01–0.01) and IBI (*β* = 0.02, 95% CI: 0.01–0.02), while a negative association was found with AAPR (*β* = −0.33, 95% CI: −0.60 to −0.06). Additionally, nonlinear relationships were identified: a J-shaped curve was observed between DII and chronic constipation in the BMI >30 and IBI Q4 subgroups, with risk escalating sharply at breakpoints of 1.89 and 1.96, respectively. A U-shaped relationship was found between DII and chronic diarrhea in the AAPR Q4 group, with risk increasing significantly beyond the breakpoint of −1.312. These findings suggest that diet-induced inflammation plays a complex role in bowel function and inflammatory regulation, interacting with factors such as BMI and inflammatory status. Reducing dietary inflammation through targeted nutritional strategies may help mitigate these risks and improve gastrointestinal health.

Unhealthy diets contribute to inflammation; for instance, research indicates that a Western diet elevates inflammatory markers like CRP and IL-6 (20), while the Mediterranean diet is associated with reduced inflammatory factors (21). The Mediterranean diet prioritizes plant-based foods like vegetables, fruits, whole grains, legumes, nuts, and olive oil, with moderate consumption of fish and poultry. It restricts pro-inflammatory foods such as red meat, processed products, and sugar. High in unsaturated fats, polyphenols, and fiber, and low in harmful fats and sugars, it helps reduce systemic inflammation and supports metabolic and cardiovascular health. In contrast, the Western diet tends to have the opposite characteristics. Pro-inflammatory diets facilitate the accumulation of inflammatory markers, compromising gut health and increasing the likelihood of gastrointestinal issues. Specific dietary components play critical roles in gut health. Fiber, for example, supports the integrity of the colonic mucus barrier. In its absence, mucosal pathogens exploit host-secreted mucin glycoproteins, increasing their ability to penetrate the epithelium and raising the risk of diarrhea (22). Conversely, anti-inflammatory dietary components like ginger have been shown to alleviate constipation due to their inflammation-reducing properties (23, 24). Low-fiber diets, often rich in processed fried foods, sugary beverages, and red meat, slow digestion, hinder stool elimination, and contribute to constipation (25). Such diets also disrupt the gut microbiota and elevate inflammatory markers, promoting low-grade chronic inflammation in the gut, which predisposes individuals to both constipation and diarrhea. Chronic inflammation weakens the gut’s defenses, making it more susceptible to infections even from minimal bacterial exposure. The “food hypothesis” suggests that pro-inflammatory diets encourage the growth of pathogenic bacteria and facultative intestinal pathogens, thereby compromising the mucosal barrier, amplifying inflammation, and exacerbating diarrhea symptoms (26). Gut dysbiosis, an imbalance in the gut microbiota, is also strongly linked to constipation (27). The gut microbiota influence intestinal motility, digestion, and nutrient absorption via the gut-brain axis. When this axis becomes strained, abnormal motility can occur, leading to prolonged food retention in the intestines. This creates an environment conducive to harmful bacterial growth, further contributing to intestinal inflammation (28). Inflammation in the gut can damage the intestinal wall, disrupt the mucosal barrier and microbiome balance, and impair motility, ultimately compromising bowel function and leading to abnormal bowel habits such as constipation. These findings underscore the importance of diet in maintaining gut health and highlight the need for targeted (29).

We also found that the Dietary Inflammatory Index (DII) was associated with specific serum inflammatory markers, including IBI, AAPR and PNLR. IBI and PNLR showed positive correlations with DII scores, while AAPR exhibited a negative correlation. These findings align with the well-recognized role of inflammation in the progression and management of cancer. Hanaha et al. identified tumor-promoting inflammation as a hallmark of cancer, emphasizing its role in disrupting tissue homeostasis (30). Inflammation represents an indispensable innate immune response to disturbances in tissue homeostasis. Chronic inflammation occurs at all stages of tumor development and has been proposed as a potential therapeutic target (31). The inflammatory biomarkers IBI, AAPR, and PNLR have demonstrated clinical relevance, particularly in colorectal cancer (CRC) (32). We hypothesize that IBI and PNLR reflect systemic inflammation and immune status. A pro-inflammatory diet may elevate these markers by promoting inflammatory factors (e.g., CRP, IL-6, TNF-*α*), increasing neutrophils, decreasing lymphocytes, and activating platelets. Although not a classical marker, AAPR serves as an indicator of both inflammation and nutritional status. Its link to DII may involve systemic inflammation, liver dysfunction, malnutrition, and metabolic syndrome. High DII diets may lower albumin, increase alkaline phosphatase, and reduce AAPR, providing a basis for further research into the diet-inflammation-disease relationship. Meta-analyses have established a correlation between DII and CRC (33), showing that higher DII scores are independently associated with an increased risk of CRC. To explore these intrinsic relationships, we examined the links between DII scores, irregular bowel habits, and inflammation-related prognostic biomarkers. Consistent with previous findings, CRC patients with higher AAPR, lower IBI, and lower PNLR have been shown to have better survival rates. This aligns with our observation that higher DII scores were associated with lower AAPR and higher IBI and PNLR in our study population, suggesting a potential intrinsic connection between DII and CRC prognosis. However, several limitations should be noted. The study included a limited number of participants with colorectal malignancies, with the majority of the cohort being cancer-free, which restricted our ability to fully demonstrate intrinsic relationships. Additionally, as a cross-sectional study, we could not establish causality between DII, CRC development, and prognosis. Future longitudinal studies are necessary to further investigate these associations and validate our findings.

A recent cross-sectional study identified a potential association between the inflammatory potential of diet, measured by the Dietary Inflammatory Index (DII), and chronic constipation (34). Research has also demonstrated a notable nonlinear relationship between BMI and constipation, with a significantly increased risk when BMI exceeds 28 kg/m^2^ (35). Consistent with these findings, our study observed a significant nonlinear relationship between DII and constipation in individuals with BMI > 30 kg/m^2^, displaying a J-shaped curve. Specifically, when DII scores exceeded 1.89, the risk of chronic constipation markedly increased, providing insight into the intrinsic connection between high BMI and constipation risk. We hypothesize that changes in adipose-derived cytokines and gastrointestinal hormones (e.g., growth hormone-releasing peptide) may influence bowel habits. Studies show that constipation in obese patients prior to bariatric surgery ranges from 8 to 21.3%, while postoperative diarrhea is more common, affecting 22.8 to 40% of patients (36). Additionally, reduced secretion of gastrointestinal hormones, such as growth hormone-releasing peptide, post-surgery is associated with early improvements in blood glucose before significant weight loss (37). This blood glucose improvement, coupled with reduced intake, promotes adipocyte breakdown, releasing fatty acids that provide energy and may indirectly impact intestinal motility and bowel habits. Similarly, in the IBIQ4 group (IBI range: 1.00–129.41), a J-shaped nonlinear relationship between DII and constipation was observed. When DII exceeded 1.96, the risk of chronic constipation significantly rose. IBI, a comprehensive inflammatory index, provides a stable and accurate reflection of the body’s inflammatory status, distinguishing individuals with varying degrees of inflammation (38). These findings suggest that individuals with high IBI already exhibit elevated inflammatory states, and when combined with pro-inflammatory dietary habits, their risk of constipation increases substantially. In the diarrhea group, we found a U-shaped relationship between DII and diarrhea in individuals within the AAPR Q4 range (0.79–3.75). Specifically, when DII scores were below −1.312, the risk of diarrhea decreased, whereas scores above −1.312 were associated with an increased risk of diarrhea. A decrease in AAPR is linked to higher mortality and recurrence rates in cancer, as lower albumin levels reflect an elevated inflammatory state, which is generally associated with poor outcomes (39, 40). Our findings indicate that among populations with high AAPR, adhering to an anti-inflammatory diet can help reduce the risk of chronic diarrhea. However, as DII scores increase beyond −1.312, the risk of diarrhea also rises. This suggests that while individuals with high AAPR may have relatively good nutritional status, pro-inflammatory diets exert a stronger influence on their internal inflammatory state, exacerbating the risk of diarrhea.

This study has several limitations. First, as a cross-sectional study, it is limited to observing associations between variables rather than establishing causal relationships. Although the observed association between the Dietary Inflammatory Index (DII) and abnormal bowel habits is statistically significant, cross-sectional data cannot confirm temporal order or underlying mechanisms. Future research should adopt longitudinal or experimental designs to explore causal pathways and temporal relationships more effectively. Second, although the data were derived from the average of two dietary interviews in the NHANES database—offering greater rigor than general dietary recall—and the DII score used has standardized, generalizable properties, recall bias remains a concern. Specifically, the DII scores were based on self-reported data from food frequency questionnaires, which can introduce recall bias and lead to misclassification of dietary exposure. Future research should aim to reduce such bias by adopting more accurate and objective dietary data collection methods. Additionally, the DII scores were derived from self-reported data collected through food frequency questionnaires, potentially introducing recall bias (41) and leading to misclassification of dietary exposure. Moreover, the classification of abnormal gut health relied solely on weekly bowel movement frequency and did not incorporate clinical investigations of gastrointestinal diseases. Critical factors such as specific medications, dietary components, and related conditions—key contributors to constipation and diarrhea—were excluded from the study due to insufficient sample size, potentially introducing bias. For example, if a high DII population also has a higher prevalence of IBD, the association between DII and bowel habits could be partially mediated by IBD rather than diet alone (42). These unmeasured confounders are common in large public databases, where data must be analyzed within available constraints. Despite these limitations, our study shows an association between DII and inflammatory markers, providing a foundation for future research. The standardized design of the DII enables integration with other databases, such as electronic health records with clinical diagnoses, for further validation. Future studies incorporating more comprehensive data on diseases, medications, and other physiological factors will enhance our understanding of bowel habits and the independent relationship between DII and bowel function.

## Conclusion

Our research identified a potential link between the Dietary Inflammatory Index (DII) and bowel habits, demonstrating that higher DII scores are associated with an increased risk of chronic constipation and diarrhea. However, the relationship between DII and abnormal bowel habits is more complex than previously understood, involving intrinsic connections to systemic serum inflammatory markers and individual factors such as BMI. These findings offer valuable insights for public health initiatives and clinical strategies aimed at preventing and managing gut health issues.

## Data Availability

The original contributions presented in the study are included in the article/Supplementary material, further inquiries can be directed to the corresponding author.

## Footnotes

^1^ www.zstats.net

^2^ http://www.empowerstats.net/analysis
