# Supplementary material for: Association between the dietary inflammatory index, bowel habits, and systemic serum inflammatory markers: insights from NHANES (2005–2010)
Source: Front Nutr. 2025 Mar 26;12:1543715. doi: 10.3389/fnut.2025.1543715 (PMC11978653; doi:10.3389/fnut.2025.1543715)
Supplement: Supplementary file 1 [file Table_1.DOCX]

Supplementary Material

# Supplementary Figures and Tables

Table1.Threshold effect analysis of DII and Constipation using two-precise linear regression

| Outcome: | BOWEL |
| --- | --- |
| model I |  |
| one-line effect | 1.250 (1.179, 1.325) <0.0001 |
| model II |  |
| breaking point (K) | 1.859 |
| < K segmentation effect 1 | 1.223 (1.150, 1.300) <0.0001 |
| > K segmentation effect 2 | 2.259 (1.317, 3.873) 0.0031 |
| Difference in effect between 2 and 1 | 1.847 (1.057, 3.228) 0.0312 |
| Predicted value of the equation at the folding point | -2.127 (-2.264, -1.991) |
| log-likelihood ratio test | 0.040 |

Table2. Threshold effects of DII and constipation in BMI subgroups analyzed using double precision linear regression

| BMI | <25 | 25-30 | >30 | Total |
| --- | --- | --- | --- | --- |
| model I |  |  |  |  |
| one-line effect | 1.266 (1.147, 1.398) <0.0001 | 1.231 (1.113, 1.360) <0.0001 | 1.286 (1.154, 1.432) <0.0001 | 1.261 (1.189, 1.338) <0.0001 |
| model II |  |  |  |  |
| breaking point (K) | -2.847 | -1.857 | 1.89 | 1.859 |
| < K segmentation effect 1 | 12.230 (0.389, 384.848) 0.1548 | 0.722 (0.450, 1.157) 0.1760 | 1.228 (1.097, 1.375) 0.0004 | 1.234 (1.160, 1.313) <0.0001 |
| > K segmentation effect 2 | 1.230 (1.107, 1.367) 0.0001 | 1.328 (1.175, 1.500) <0.0001 | 3.318 (1.531, 7.191) 0.0024 | 2.270 (1.321, 3.903) 0.0030 |
| Difference in effect between 2 and 1 | 0.101 (0.003, 3.239) 0.1948 | 1.840 (1.072, 3.156) 0.0269 | 2.701 (1.202, 6.071) 0.0162 | 1.840 (1.050, 3.224) 0.0331 |
| Predicted value of the equation at the folding point | -2.840 (-3.174, -2.505) | -3.124 (-3.424, -2.825) | -2.283 (-2.517, -2.049) | -2.127 (-2.264, -1.991) |
| log-likelihood ratio test | 0.092 | 0.035 | 0.024 | 0.042 |

Table3. Threshold effects of DII and constipation in IBI subgroups analyzed using double precision linear regression

| IBI | Q1 | Q2 | Q3 | Q4 | Total |
| --- | --- | --- | --- | --- | --- |
| model I |  |  |  |  |  |
| one-line effect | 1.197 (1.069, 1.341) 0.0018 | 1.340 (1.190, 1.509) <0.0001 | 1.247 (1.100, 1.415) 0.0006 | 1.228 (1.091, 1.383) 0.0007 | 1.252 (1.180, 1.328) <0.0001 |
| model II |  |  |  |  |  |
| breaking point (K) | -0.124 | -0.544 | 1.113 | 1.96 | 1.859 |
| < K segmentation effect 1 | 1.066 (0.867, 1.311) 0.5432 | 1.529 (1.120, 2.086) 0.0075 | 1.383 (1.168, 1.638) 0.0002 | 1.145 (1.013, 1.294) 0.0307 | 1.225 (1.151, 1.304) <0.0001 |
| > K segmentation effect 2 | 1.396 (1.078, 1.808) 0.0114 | 1.243 (1.017, 1.519) 0.0337 | 0.687 (0.359, 1.316) 0.2578 | 5.794 (2.359, 14.228) 0.0001 | 2.255 (1.315, 3.867) 0.0031 |
| Difference in effect between 2 and 1 | 1.309 (0.868, 1.974) 0.1986 | 0.813 (0.522, 1.268) 0.3610 | 0.497 (0.238, 1.038) 0.0629 | 5.061 (1.982, 12.921) 0.0007 | 1.840 (1.053, 3.217) 0.0323 |
| Predicted value of the equation at the folding point | -2.654 (-2.952, -2.355) | -2.538 (-2.827, -2.248) | -2.160 (-2.428, -1.893) | -2.215 (-2.481, -1.950) | -2.127 (-2.264, -1.991) |
| log-likelihood ratio test | 0.201 | 0.355 | 0.052 | <0.001 | 0.041 |

Table4.Threshold effect analysis of DII and Diarrhea using two-precise linear regression

| Outcome: | BOWEL |
| --- | --- |
| model I |  |
| one-line effect | 1.078 (1.020, 1.139) 0.0079 |
| model II |  |
| breaking point (K) | -0.118 |
| < K segmentation effect 1 | 0.992 (0.896, 1.098) 0.8739 |
| > K segmentation effect 2 | 1.198 (1.060, 1.355) 0.0038 |
| Difference in effect between 2 and 1 | 1.208 (0.993, 1.470) 0.0588 |
| Predicted value of the equation at the folding point | -2.583 (-2.724, -2.442) |
| log-likelihood ratio test | 0.060 |

Table5. Threshold effects of DII and Diarrhea in AAPR subgroups analyzed using double precision linear regression

| AAPR | Q1 | Q2 | Q3 | Q4 | Total |
| --- | --- | --- | --- | --- | --- |
| model I |  |  |  |  |  |
| one-line effect | 1.125 (1.011, 1.252) 0.0314 | 1.130 (1.011, 1.262) 0.0307 | 0.992 (0.886, 1.111) 0.8922 | 1.033 (0.918, 1.164) 0.5873 | 1.073 (1.014, 1.134) 0.0136 |
| model II |  |  |  |  |  |
| breaking point (K) | -1.813 | -2.741 | -1.934 | -1.312 | -0.118 |
| < K segmentation effect 1 | 0.953 (0.522, 1.740) 0.8750 | 0.460 (0.146, 1.455) 0.1865 | 1.354 (0.729, 2.514) 0.3379 | 0.657 (0.478, 0.901) 0.0093 | 0.989 (0.894, 1.095) 0.8324 |
| > K segmentation effect 2 | 1.148 (1.008, 1.307) 0.0375 | 1.171 (1.038, 1.321) 0.0105 | 0.948 (0.822, 1.093) 0.4616 | 1.266 (1.057, 1.518) 0.0106 | 1.190 (1.052, 1.345) 0.0057 |
| Difference in effect between 2 and 1 | 1.205 (0.617, 2.350) 0.5853 | 2.543 (0.765, 8.448) 0.1277 | 0.700 (0.351, 1.396) 0.3115 | 1.928 (1.239, 3.001) 0.0036 | 1.203 (0.988, 1.464) 0.0656 |
| Predicted value of the equation at the folding point | -2.525 (-2.841, -2.209) | -2.820 (-3.189, -2.450) | -2.450 (-2.759, -2.141) | -3.036 (-3.369, -2.703) | -2.583 (-2.724, -2.442) |
| log-likelihood ratio test | 0.592 | 0.155 | 0.295 | 0.004 | 0.067 |

# Supplementary Data

**The relevant code for threshold effect analysis between DII and Constipation in EmpowerStats version 3.0 (http://www.empowerstats.net/analysis).**

Sys.setlocale("LC_TIME", "C")

library(doBy,lib.loc=R.LibLocation)

library(plotrix,lib.loc=R.LibLocation)

library(stringi,lib.loc=R.LibLocation)

library(stringr,lib.loc=R.LibLocation)

library(survival,lib.loc=R.LibLocation)

library(rms,lib.loc=R.LibLocation)

library(nnet,lib.loc=R.LibLocation)

library(car,lib.loc=R.LibLocation)

library(mgcv,lib.loc=R.LibLocation)

pdfwd<-6; pdfht<-6

setwd("C:/Users/79156/Desktop/nhances2005-2010/PROJ7_2_tbl")

load("C:/Users/79156/Desktop/nhances2005-2010/bianmi_SZ.Rdata")

if (length(which(ls()=="EmpowerStatsR"))==0) EmpowerStatsR<-get(ls()[1])

names(EmpowerStatsR)<-toupper(names(EmpowerStatsR))

#--#

vname<-c("_N_","_STAT_","_TOTAL_","SEQN","WEIGHT","GENDER","GENDER.1","GENDER.2")

vlabel<-c("样本量(%)","统计量","合计","SEQN","WEIGHT","GENDER"," 1"," 2")

vname<-c(vname,"YEAR","RACE","RACE.1","RACE.2","RACE.3","RACE.4","RACE.5")

vlabel<-c(vlabel,"YEAR","RACE"," 1"," 2"," 3"," 4"," 5")

vname<-c(vname,"BOWEL","BOWEL.0","BOWEL.1","CANCER","CANCER.0","CANCER.1")

vlabel<-c(vlabel,"BOWEL"," 0"," 1","CANCER"," 0"," 1")

vname<-c(vname,"ALBUMIN","ALT","AST","ALKALINE_PHOSPHATASE")

vlabel<-c(vlabel,"ALBUMIN","ALT","AST","ALKALINE_PHOSPHATASE")

vname<-c(vname,"CHOLESTEROL","GST","TOTAL.PROTEIN","TRIGLYCERIDE")

vlabel<-c(vlabel,"CHOLESTEROL","GST","TOTAL.PROTEIN","TRIGLYCERIDE")

vname<-c(vname,"GLOBULIN","WBC","LYMPHOCYTE","MONOCYTE","NEUTROPHIL")

vlabel<-c(vlabel,"GLOBULIN","WBC","LYMPHOCYTE","MONOCYTE","NEUTROPHIL")

vname<-c(vname,"EOSINOPHIL","RBC","HB","PLT","CRP","INFLAMMATORY")

vlabel<-c(vlabel,"EOSINOPHIL","RBC","HB","PLT","CRP","INFLAMMATORY")

vname<-c(vname,"SOMKING","SOMKING.0","SOMKING.1","SOMKING.2")

vlabel<-c(vlabel,"SOMKING"," 0"," 1"," 2")

vname<-c(vname,"DIET","DII","BMI","BMI.1","BMI.2","BMI.3")

vlabel<-c(vlabel,"DIET","DII","BMI"," 1"," 2"," 3")

vname<-c(vname,"ALBUMIN_QUANTILE","ALBUMIN_QUANTILE.1","ALBUMIN_QUANTILE.2","ALBUMIN_QUANTILE.3","ALBUMIN_QUANTILE.4")

vlabel<-c(vlabel,"ALBUMIN_QUANTILE"," 1"," 2"," 3"," 4")

vname<-c(vname,"ALT_QUANTILE","ALT_QUANTILE.1","ALT_QUANTILE.2","ALT_QUANTILE.3","ALT_QUANTILE.4")

vlabel<-c(vlabel,"ALT_QUANTILE"," 1"," 2"," 3"," 4")

vname<-c(vname,"ALKALINE_PHOSPHATASE_QUANTILE","ALKALINE_PHOSPHATASE_QUANTILE.1","ALKALINE_PHOSPHATASE_QUANTILE.2","ALKALINE_PHOSPHATASE_QUANTILE.3","ALKALINE_PHOSPHATASE_QUANTILE.4")

vlabel<-c(vlabel,"ALKALINE_PHOSPHATASE_QUANTILE"," 1"," 2"," 3"," 4")

vname<-c(vname,"GLOBULIN_QUANTILE","GLOBULIN_QUANTILE.1","GLOBULIN_QUANTILE.2","GLOBULIN_QUANTILE.3","GLOBULIN_QUANTILE.4")

vlabel<-c(vlabel,"GLOBULIN_QUANTILE"," 1"," 2"," 3"," 4")

vname<-c(vname,"WBC_QUANTILE","WBC_QUANTILE.1","WBC_QUANTILE.2","WBC_QUANTILE.3","WBC_QUANTILE.4")

vlabel<-c(vlabel,"WBC_QUANTILE"," 1"," 2"," 3"," 4")

vname<-c(vname,"NEUTROPHIL_QUANTILE","NEUTROPHIL_QUANTILE.1","NEUTROPHIL_QUANTILE.2","NEUTROPHIL_QUANTILE.3","NEUTROPHIL_QUANTILE.4")

vlabel<-c(vlabel,"NEUTROPHIL_QUANTILE"," 1"," 2"," 3"," 4")

vname<-c(vname,"RBC_QUANTILE","RBC_QUANTILE.1","RBC_QUANTILE.2","RBC_QUANTILE.3","RBC_QUANTILE.4")

vlabel<-c(vlabel,"RBC_QUANTILE"," 1"," 2"," 3"," 4")

vname<-c(vname,"HB_QUANTILE","HB_QUANTILE.1","HB_QUANTILE.2","HB_QUANTILE.3","HB_QUANTILE.4")

vlabel<-c(vlabel,"HB_QUANTILE"," 1"," 2"," 3"," 4")

vname<-c(vname,"PLT_QUANTILE","PLT_QUANTILE.1","PLT_QUANTILE.2","PLT_QUANTILE.3","PLT_QUANTILE.4")

vlabel<-c(vlabel,"PLT_QUANTILE"," 1"," 2"," 3"," 4")

vname<-c(vname,"CRP_QUANTILE","CRP_QUANTILE.1","CRP_QUANTILE.2","CRP_QUANTILE.3","CRP_QUANTILE.4")

vlabel<-c(vlabel,"CRP_QUANTILE"," 1"," 2"," 3"," 4")

vname<-c(vname,"AAPR","AAPR.1","AAPR.2","AAPR.3","AAPR.4")

vlabel<-c(vlabel,"AAPR"," 1"," 2"," 3"," 4")

vname<-c(vname,"NLR","NLR.1","NLR.2","NLR.3","NLR.4","PNLR","PNLR.1","PNLR.2","PNLR.3","PNLR.4")

vlabel<-c(vlabel,"NLR"," 1"," 2"," 3"," 4","PNLR"," 1"," 2"," 3"," 4")

vname<-c(vname,"LMR","LMR.1","LMR.2","LMR.3","LMR.4","LCR","LCR.1","LCR.2","LCR.3","LCR.4")

vlabel<-c(vlabel,"LMR"," 1"," 2"," 3"," 4","LCR"," 1"," 2"," 3"," 4")

vname<-c(vname,"PLR","PLR.1","PLR.2","PLR.3","PLR.4","IBI","IBI.1","IBI.2","IBI.3","IBI.4")

vlabel<-c(vlabel,"PLR"," 1"," 2"," 3"," 4","IBI"," 1"," 2"," 3"," 4")

slt.vname<-c()

library(MASS,lib.loc=R.LibLocation)

library(gdata,lib.loc=R.LibLocation)

library(geepack,lib.loc=R.LibLocation)

library(mgcv,lib.loc=R.LibLocation)

ofname<-"PROJ7_2_tbl";

WD<-EmpowerStatsR; wd.subset="";

svy.DSN.YN <- FALSE;

weights<-1;weights.var <- NA;

WD<-cbind(WD,weights); WD<-WD[!is.na(weights),];

title<-"阈值效应分析";

attach(WD)

subjvname<-NA;

yv<-cbind(BOWEL);

yvname<-c('BOWEL');

yvar<-c('BOWEL');

ydist<-c('binomial');

ylink<-c('logit');

ylv<-c(2);

xv<-cbind(DII);

xvname<-c('DII');

xvar<-c('DII');

xlv<-c(0);

sxf<-NA;

svname<-NA; sv<-NA; slv<-NA;

av<-NA; avname<-NA; avlbl<-NA; nadj<-0; alv<-NA;

timev<-NA; timevname<-NA;

bv<-NA; bvar<-NA;

colv<-NA; colvname<-NA;

v.start<-NA; vname.start<-NA;

v.stop<-NA; vname.stop<-NA;

par1<-"自动寻找最佳拐点";dec<-3;parm<-c(NA, NA, 1,NA, 0);

if (!exists("pdfwd")) pdfwd<-6;

if (!exists("pdfht")) pdfht<-6;

##R package## MASS gdata geepack mgcv ##R package##;

pvformat<-function(p,dec) {

pp <- sprintf(paste("%.",dec,"f",sep=""),as.numeric(p))

if (is.matrix(p)) {pp<-matrix(pp, nrow=nrow(p)); colnames(pp)<-colnames(p);rownames(pp)<-rownames(p);}

lw <- paste("<",substr("0.00000000000",1,dec+1),"1",sep="");

pp[as.numeric(p)<(1/10^dec)]<-lw

return(pp)

}

numfmt<-function(p,dec) {

if (is.list(p)) p<-as.matrix(p)

pp <- sprintf(paste("%.",dec,"f",sep=""),as.numeric(p))

if (is.matrix(p)) {pp<-matrix(pp, nrow=nrow(p));colnames(pp)<-colnames(p);rownames(pp)<-rownames(p);}

pp[as.numeric(p)>10000000]<- "inf."

pp[is.na(p) | gsub(" ","",p)==""]<- ""

pp[p=="-Inf"]<-"-Inf"

pp[p=="Inf"]<-"Inf"

return(pp)

}

mat2htmltable<-function(mat) {

t1<- apply(mat,1,function(z) paste(z,collapse="</td><td>"))

t2<- paste("<tr><td>",t1,"</td></tr>")

return(paste(t2,collapse=" "))

}

setgam<-function(fml,yi,wdtmp) {

if (ydist[yi]=="") ydist[yi]<-"gaussian"

if (ydist[yi]=="exact") ydist[yi]<-"binomial"

if (ydist[yi]=="breslow") ydist[yi]<-"binomial"

if (ydist[yi]=="gaussian") mdl<-try(gam(formula(fml),weights=wdtmp$weights,data=wdtmp, family=gaussian(link="identity")))

if (ydist[yi]=="binomial") mdl<-try(gam(formula(fml),weights=wdtmp$weights,data=wdtmp, family=binomial(link="logit")))

if (ydist[yi]=="poisson") mdl<-try(gam(formula(fml),weights=wdtmp$weights,data=wdtmp, family=poisson(link="log")))

if (ydist[yi]=="gamma") mdl<-try(gam(formula(fml),weights=wdtmp$weights,data=wdtmp, family=Gamma(link="inverse")))

if (ydist[yi]=="negbin") mdl<-try(gam(formula(fml),weights=wdtmp$weights,data=wdtmp, family=negbin(c(1,10), link="log")))

return(mdl)

}

setgee<-function(fml,yi, wdtmp) {

if (ydist[yi]=="") ydist[yi]<-"gaussian"

if (ydist[yi]=="exact") ydist[yi]<-"binomial"

if (ydist[yi]=="breslow") ydist[yi]<-"binomial"

if (ydist[yi]=="gaussian") md<-try(geeglm(formula(fml),id=wdtmp[,subjvname],corstr=gee.TYPE,family="gaussian",weights=wdtmp$weights,data=wdtmp))

if (ydist[yi]=="binomial") md<-try(geeglm(formula(fml),id=wdtmp[,subjvname],corstr=gee.TYPE,family="binomial",weights=wdtmp$weights,data=wdtmp))

if (ydist[yi]=="poisson") md<-try(geeglm(formula(fml),id=wdtmp[,subjvname],corstr=gee.TYPE,family="poisson",weights=wdtmp$weights,data=wdtmp))

if (ydist[yi]=="gamma") md<-try(geeglm(formula(fml),id=wdtmp[,subjvname],corstr=gee.TYPE,family="Gamma",weights=wdtmp$weights,data=wdtmp))

if (ydist[yi]=="negbin") md<-try(geeglm.nb(formula(fml),id=wdtmp[,subjvname],corstr=gee.TYPE,weights=wdtmp$weights,data=wdtmp))

return(md)

}

setglm<-function(fml,yi, wdtmp) {

if (ydist[yi]=="") ydist[yi]<-"gaussian"

if (ydist[yi]=="exact") ydist[yi]<-"binomial"

if (ydist[yi]=="breslow") ydist[yi]<-"binomial"

if (ydist[yi]=="gaussian") md<-try(glm(formula(fml),family="gaussian",weights=wdtmp$weights,data=wdtmp))

if (ydist[yi]=="binomial") md<-try(glm(formula(fml),family="binomial",weights=wdtmp$weights,data=wdtmp))

if (ydist[yi]=="poisson") md<-try(glm(formula(fml),family="poisson",weights=wdtmp$weights,data=wdtmp))

if (ydist[yi]=="gamma") md<-try(glm(formula(fml),family="Gamma",weights=wdtmp$weights,data=wdtmp))

if (ydist[yi]=="negbin") md<-try(glm.nb(formula(fml),weights=wdtmp$weights,data=wdtmp))

return(md)

}

mdl2oo<-function(mdl, xxname, opt) {

if (is.na(mdl[[1]][1])) return(rep(" ",times=length(xxname)))

if (substr(mdl[[1]][1],1,5)=="Error") return(rep(" ",times=length(xxname)))

decp<-dec+2; if (decp>4) decp<-4

gs<-summary(mdl); print(mdl$formula); print(gs)

if (opt=="gam") {gsparm <- gs$p.table; } else {gsparm <- gs$coefficients;}

gsp<-gsparm[match(xxname,rownames(gsparm)),]

if (length(xxname)==1) {beta<-gsp[1]; se<-gsp[2]; pv<-gsp[4];

} else {beta<-gsp[,1]; se<-gsp[,2]; pv<-gsp[,4]; }

ci1<- beta-1.96*se; ci2<- beta+1.96*se

pvx<-substr(rep("****",length(pv)),1,(pv<=0.05)+(pv<=0.01)+(pv<=0.001))

if (colprn==3) {pvv<-pvx;} else {pvv<-pvformat(pv,decp);}

if ((colprn!=2) & (gs$family[[2]]=="log" | gs$family[[2]]=="logit")) {

o1<-paste(numfmt(exp(beta),dec)," (",numfmt(exp(ci1),dec),", ",numfmt(exp(ci2),dec),")",sep="")

} else {

if (colprn<3) {o1<-paste(numfmt(beta,dec), " (",numfmt(ci1,dec),", ",numfmt(ci2,dec),")",sep="")

} else {o1<-paste(numfmt(beta,dec), "+",numfmt(se,dec),sep="");}

}

o1<-paste(o1,pvv); o1[is.na(beta)]<-NA

return(o1)

}

removeNA<-function(i,j,wdf) {

vvv<-c(yvname[i],xvname[j],avname,subjvname,bvar,vname.start,vname.stop,timevname);

vvv<-vvv[!is.na(vvv)]; vvv<-vvv[vvv>" "]

tmp<-is.na(wdf[,vvv]);

return(wdf[apply(tmp,1,sum)==0,])

}

get.tpval<-function(i,j,g,opt,wdtmp, tppmin=NA, tppmax=NA) {

if (is.na(wdtmp)) {

if (is.na(g)) {wdtmp<-removeNA(i,j,WD);

} else if (g<nblv) {wdtmp0<-WD[WD[,bvar]==blv[g],]; wdtmp<-removeNA(i,j,wdtmp0);

} else {wdtmp<-removeNA(i,j,WD); }

}

if (is.na(g)) {fmladj1<-fmladj;

} else if (g<nblv) {fmladj1<-fmladj;

} else {fmladj1<-paste(fmladj,"+factor(",bvar,")",sep="");}

xTMP <- wdtmp[,xvname[j]]

tmp.ss<-seq(0.05,0.95,0.05)

tp<-quantile(xTMP,probs=tmp.ss,na.rm=TRUE)

tmp.llk<-rep(NA,length(tmp.ss))

fml<-paste(yvname[i],"~",xvname[j],"+tmp.X",fmladj1)

if (!is.na(tppmin) & !is.na(tppmax)) {

tp2.min = tppmin; tp2.max = tppmax;

} else {

for (k in (1:length(tmp.ss))) {

tmp.X<-(xTMP > tp[k])*(xTMP-tp[k]); wdtmp1<-cbind(wdtmp,tmp.X)

if (opt=="glm" | opt=="gee") tmp.mdl<-setglm(fml, i, wdtmp1);

if (opt=="gam") tmp.mdl<-setgam(fml, i, wdtmp1);

tmp.llk[k]<-logLik(tmp.mdl)

rm(wdtmp1, tmp.X)

}

tp1<-tmp.ss[which.max(tmp.llk)]

tp2.min = tp1 - 0.04

tp2.max = tp1 + 0.04

if (tp2.min<0.05) {tp2.min=0.05}

if (tp2.max>0.95) {tp2.max=0.95}

}

tp.pctlrange<-quantile(xTMP,probs=c(tp2.min,tp2.max),na.rm=TRUE)

tp.range<-unique(xTMP[xTMP>tp.pctlrange[1] & xTMP<tp.pctlrange[2]])

while (length(tp.range)>5) {

tmp.pct3<-quantile(tp.range,probs=c(0,0.25,0.5,0.75,1),type=3)

tmp.llk3<-rep(NA,3)

for (k in (2:4)) {

tmp.X<-(xTMP>tmp.pct3[k])*(xTMP-tmp.pct3[k]); wdtmp1<-cbind(wdtmp,tmp.X)

if (opt=="glm" | opt=="gee") tmp.mdl<-setglm(fml, i, wdtmp1);

if (opt=="gam") tmp.mdl<-setgam(fml, i, wdtmp1);

tmp.llk3[k-1]<-logLik(tmp.mdl)

rm(wdtmp1, tmp.X)

}

tmp.min3<-which.max(tmp.llk3)

tp.range<-tp.range[tp.range>=tmp.pct3[tmp.min3] & tp.range<=tmp.pct3[tmp.min3+2]]

}

if (length(tp.range)>0) {

if (length(tp.range)==1) {tp.val=tp.range[1];} else {

tmp.llk<-rep(NA,length(tp.range))

for (k in (1:length(tp.range))) {

tmp.X<-(xTMP>tp.range[k])*(xTMP-tp.range[k]); wdtmp1<-cbind(wdtmp,tmp.X)

if (opt=="glm" | opt=="gee") tmp.mdl<-setglm(fml, i, wdtmp1);

if (opt=="gam") tmp.mdl<-setgam(fml, i, wdtmp1);

tmp.llk[k]<-logLik(tmp.mdl)

rm(wdtmp1, tmp.X)

}

tp.val<-tp.range[which.max(tmp.llk)]

}

} else { tp.val<-tp.pctlrange[1];}

return(round(tp.val,dec));

}

get2lines<-function(i,j,g,tp.value,opt) {

if (is.na(g)) {fmladj1<-fmladj;wdtmp<-removeNA(i,j,WD);

} else if (g<nblv) {fmladj1<-fmladj;wdtmp0<-WD[WD[,bvar]==blv[g],];wdtmp<-removeNA(i,j,wdtmp0);

} else {fmladj1<-paste(fmladj,"+factor(",bvar,")",sep="");wdtmp<-removeNA(i,j,WD);}

xTMP<-wdtmp[,xvname[j]]

tmp.X1<-(xTMP<=tp.value)*(xTMP-tp.value)

tmp.X2<-(xTMP> tp.value)*(xTMP-tp.value)

wdtmp1<-cbind(wdtmp,xTMP,tmp.X1,tmp.X2)

fml0<-paste(yvname[i],"~xTMP+tmp.X2",fmladj1)

fml1<-paste(yvname[i],"~tmp.X1+tmp.X2",fmladj1)

fml2<-paste(yvname[i],"~xTMP",fmladj1)

fmlp<-paste(yvname[i],"~xTMP+tmp.X2")

tmpn<-nrow(wdtmp)

if (opt=="glm") {

tmp.mdl0<-setglm(fml0,i,wdtmp1); tmp.mdl1<-setglm(fml1,i,wdtmp1)

tmp.mdl2<-setglm(fml2,i,wdtmp1); tmp.mdlp<-setglm(fmlp,i,wdtmp1)

}

if (opt=="gam") {

tmp.mdl0<-setgam(fml0,i,wdtmp1); tmp.mdl1<-setgam(fml1,i,wdtmp1)

tmp.mdl2<-setgam(fml2,i,wdtmp1); tmp.mdlp<-setgam(fmlp,i,wdtmp1)

}

if (opt=="gee") {

tmp.mdl0<-setgee(fml0,i,wdtmp1); tmp.mdl1<-setgee(fml1,i,wdtmp1)

tmp.mdl2<-setgee(fml2,i,wdtmp1); tmp.mdlp<-setglm(fmlp,i,wdtmp1)

}

pd<-predict(tmp.mdlp,data.frame(xTMP=tp.value,tmp.X2=0), se.fit=TRUE)

prd<-paste(numfmt(pd$fit,dec)," (",numfmt(pd$fit-1.96*pd$se.fit,dec),", ", numfmt(pd$fit+1.96*pd$se.fit, dec),")",sep="")

m2<-mdl2oo(tmp.mdl2,"xTMP",opt)

m1<-mdl2oo(tmp.mdl1,c("tmp.X1","tmp.X2"),opt)

m0<-mdl2oo(tmp.mdl0,"tmp.X2",opt)

if (opt=="gee") {

plrt<-try(anova(tmp.mdl0,tmp.mdl2)$"P(>|Chi|)",TRUE)

plrt<-ifelse((plrt<="9" && plrt>="0"), pvformat(plrt,3),"-")

} else {plrt<- pvformat(1-pchisq(2*(logLik(tmp.mdl0)[1]-logLik(tmp.mdl2)[1]),1),3);}

oo<-list(c("",m2,"",tp.value,m1,m0,prd,plrt),tmpn)

return(oo)

}

get3lines<-function(i,j,g,tp.value,opt) {

if (is.na(g)) {fmladj1<-fmladj;wdtmp<-removeNA(i,j,WD);

} else if (g<nblv) {fmladj1<-fmladj;wdtmp0<-WD[WD[,bvar]==blv[g],];wdtmp<-removeNA(i,j,wdtmp0);

} else {fmladj1<-paste(fmladj,"+factor(",bvar,")",sep="");wdtmp<-removeNA(i,j,WD);}

xTMP<-wdtmp[,xvname[j]]; tp1<-tp.value[1]; tp2<-tp.value[2]

tmp.X1<- (xTMP< tp1)*(xTMP-tp1)

tmp.X2<-((xTMP>=tp1) & (xTMP<=tp2))*(xTMP-tp1)

tmp.X3<- (xTMP> tp2)*(xTMP-tp2)

tmp.B1<- (xTMP>tp2)

wdtmp1<-cbind(wdtmp,xTMP,tmp.X1,tmp.X2,tmp.X3,tmp.B1)

fml0<-paste(yvname[i],"~xTMP+tmp.X1+tmp.X3+tmp.B1",fmladj1)

fml1<-paste(yvname[i],"~tmp.X1+tmp.X2+tmp.X3+tmp.B1",fmladj1)

fml2<-paste(yvname[i],"~xTMP",fmladj1)

tmpn<-nrow(wdtmp)

if (opt=="glm") {

tmp.mdl0<-setglm(fml0,i,wdtmp1); tmp.mdl1<-setglm(fml1,i,wdtmp1); tmp.mdl2<-setglm(fml2,i,wdtmp1);

}

if (opt=="gam") {

tmp.mdl0<-setgam(fml0,i,wdtmp1); tmp.mdl1<-setgam(fml1,i,wdtmp1); tmp.mdl2<-setgam(fml2,i,wdtmp1);

}

if (opt=="gee") {

tmp.mdl0<-setgee(fml0,i,wdtmp1); tmp.mdl1<-setgee(fml1,i,wdtmp1); tmp.mdl2<-setgee(fml2,i,wdtmp1);

}

m2<-mdl2oo(tmp.mdl2,"xTMP",opt)

m1<-mdl2oo(tmp.mdl1,c("tmp.X1","tmp.X2","tmp.X3"),opt)

m0<-mdl2oo(tmp.mdl0,c("tmp.X1","tmp.X3"),opt)

if (opt=="gee") {

plrt<-try(anova(tmp.mdl0,tmp.mdl2)$"P(>|Chi|)",TRUE)

plrt<-ifelse((plrt<="9" && plrt>="0"), pvformat(plrt,3),"-")

} else {plrt<- pvformat(1-pchisq(2*(logLik(tmp.mdl0)[1]-logLik(tmp.mdl2)[1]),1),3);}

oo<-list(c("",m2,"",paste(tp.value,collapse=", "),m1,m0,plrt),tmpn)

return(oo)

}

getci4tp<-function(i,j,g,opt,tp0=NA) {

set.seed(123456)

if (is.na(g)) {wdt<-removeNA(i,j,WD);

} else if (g<nblv) {wdt<-WD[WD[,bvar]==blv[g],];wdt<-removeNA(i,j,wdt);

} else {wdt<-removeNA(i,j,WD);}

nnwd<-nrow(wdt); tp.vv<-rep(NA,1000)

if (!is.na(tp0)) {

tpp0 = sum(wdt[,xvname[j]] < tp0)/length(wdt[,xvname[j]])

tppmin = max(tpp0 - 0.09, 0.05)

tppmax = min(tpp0 + 0.09, 0.95)

} else {

tppmin = NA; tppmax = NA

}

for (s in (1:1000)) {

WDi<-wdt[sample(1:nnwd,nnwd,replace=T),]

tp.vv[s]<-get.tpval(i, j, NA, opt, WDi, tppmin, tppmax); rm(WDi)

}

tpci<-paste(quantile(tp.vv,probs=c(0.025,0.975)),collapse=", ")

return(tpci);

}

vlabelN<-(substr(vlabel,1,1)==" ");

vlabelZ<-vlabel[vlabelN];vlabelV<-vlabel[!vlabelN]

vnameV<-vname[!vlabelN];vnameZ<-vname[vlabelN];

allvname<-c(yvname,xvname,bvar,avname,subjvname,vname.start,vname.stop,timevname,"weights");

allvname<-allvname[!is.na(allvname)]

WD<-WD[,allvname];

w<-c("<html><head>","<meta http-equiv=\"Content-Type\" content=\"text/html\" charset=\"gb2312\" /></head><body>")

if (!is.na(avname[1])) {

if (sum((saf=="s" | saf=="S") & alv>0)>0) w<-c(w,"</br>Spline smoothing only applies for continuous variables")

if (!is.na(subjvname)) saf<-rep(0,length(saf))

}

if (sum(xlv>0)>0) w<-c(w,"Categorical exposure variables were ignored")

xvname<-xvname[xlv==0];

if (!is.na(subjvname)) WD<-WD[order(WD[,subjvname]),];

fmladj<-""; avb=""; smoothav<-0;

if (!is.na(avname[1])) {

avb<-vlabelV[match(avname,vnameV)];

avname_ <- avname

smoothavi<-((saf=="s" | saf=="S") & alv==0)

smoothav<-sum(smoothavi)

avname_[smoothavi]<-paste("s(",avname[smoothavi],")",sep="")

avb1<-avb

avb1[smoothavi]<-paste(avb[smoothavi],"(Smooth)",sep="")

avname_[alv>0]<-paste("factor(",avname[alv>0],")",sep="")

fmladj<-paste("+",paste(avname_,collapse="+"))

}

if (is.na(bvar)) {

blvb<-"N"; blvb_<-"N"; nblv<-1; blbl<-"";

} else {

blbl<-vlabelV[match(bvar,vnameV)]; if (is.na(blbl)) blbl<-bvar;

blv<-levels(factor(WD[,bvar])); nblv<-length(blv)+1

blvb_<-vlabelZ[match(paste(bvar,".",blv,sep=""),vnameZ)];

blvb_[is.na(blvb_)]<-blv[is.na(blvb_)];

blvb<-c(paste(blbl,blvb_,sep="="),"Total");

blvb_<-c(blvb_,"Total")

WD<-WD[!is.na(WD[,bvar]),]

}

ny=length(yvname); nx=length(xvname);

xb<-vlabelV[match(xvname,vnameV)]; xb[is.na(xb)]<-xvname[is.na(xb)]

yb<-vlabelV[match(yvname,vnameV)]; yb[is.na(yb)]<-yvname[is.na(yb)]

opt<-ifelse(!is.na(subjvname), "gee", ifelse(smoothav>0, "gam", "glm")) ;

colprn<-parm[3]

if (is.na(par1)) par1<-"";

if (is.numeric(par1)) {tp.vv<-par1;

} else {

tmp<-as.numeric(strsplit(par1," ")[[1]]); tp.vv<-c(tmp[!is.na(tmp)],NA)

}

prn<-ifelse(!is.na(bvar), "S", ifelse(nx>1 & ny==1, "X", "Y"));

if (length(tp.vv)>2) tp.vv<-tp.vv[1:2]

ntp<-length(tp.vv);

getci<-FALSE

prnopt<-c("β (95%CI) Pvalue / OR (95%CI) Pvalue", "β (95%CI) Pvalue", "β+se / OR (95%CI) *P<0.05 **P<0.01 ***P<0.001")

if (ntp==1) {

cc0<-c("模型 I","&nbsp&nbsp一条直线效应");

cc0<-c(cc0,"模型 II","&nbsp&nbsp折点(K)","&nbsp&nbsp &lt K 段效应 1","&nbsp&nbsp &gt K 段效应 2","&nbsp&nbsp 2与1的效应差")

cc0<-c(cc0,"&nbsp&nbsp折点处方程预测值")

if (is.na(tp.vv[1]) & !is.na(parm[1])) getci<-TRUE;

} else {

cc0<-c("模型 I","&nbsp&nbsp一条直线效应");

cc0<-c(cc0,"模型 II","&nbsp&nbsp折点(K1,K2)","&nbsp&nbsp &lt K1 段效应 1","&nbsp&nbsp K1-K2 段效应 2","&nbsp&nbsp &gt K2 段效应 3")

cc0<-c(cc0,"&nbsp&nbsp 1与2的效应差","&nbsp&nbsp 3与2的效应差")

}

if (opt=="gee") {cc0<-c(cc0,"ANOVA 两模型比较");} else {cc0<-c(cc0,"对数似然比检验");}

if (getci) cc0<-c(cc0,"折点的95可信区间");

sink(paste(ofname,".lst",sep=""))

w<-c(w,paste("<h2>", title, "</h2>"))

nn<-c("Outcome","Exposure",blvb);

if (prn=="Y") {

for (j in 1:nx) {

tt<-cc0;

for (i in 1:ny) {

if (is.na(tp.vv[1])) {tp.v<-get.tpval(i,j,NA,opt,NA);} else {tp.v<-tp.vv;}

if (ntp==1) tmpij<-get2lines(i,j,NA,tp.v,opt);

if (ntp==2) tmpij<-get3lines(i,j,NA,tp.v,opt);

if (getci) {tt<-cbind(tt,c(tmpij[[1]],getci4tp(i,j,NA,opt,tp.v)));} else {tt<-cbind(tt,tmpij[[1]]);}

nn<-rbind(nn,c(yb[i],xb[j],tmpij[[2]]))

}

tt<-rbind(c("Outcome: ",yb),tt)

w<-c(w,paste("</br>For exposure:",xb[j]))

w<-c(w,"</br><table border=3>", mat2htmltable(tt), "</table>")

}

}

if (prn=="X") {

for (i in 1:ny) {

tt<-cc0;

for (j in 1:nx) {

if (is.na(tp.vv[1])) {tp.v<-get.tpval(i,j,NA,opt,NA);} else {tp.v<-tp.vv;}

if (ntp==1) tmpij<-get2lines(i,j,NA,tp.v,opt);

if (ntp==2) tmpij<-get3lines(i,j,NA,tp.v,opt);

if (getci) {tt<-cbind(tt,c(tmpij[[1]],getci4tp(i,j,NA,opt,tp.v)));} else {tt<-cbind(tt,tmpij[[1]]);}

nn<-rbind(nn,c(yb[i],xb[j],tmpij[[2]]))

}

tt<-rbind(c("Exposure: ",xb),tt)

w<-c(w,paste("</br>For outcome:",yb[i]))

w<-c(w,"</br><table border=3>", mat2htmltable(tt), "</table>")

}

}

if (prn=="S") {

for (i in 1:ny) {

tt<-cc0;

for (j in 1:nx) {

nnij<-c(yb[i],xb[j])

for (g in 1:nblv) {

if (is.na(tp.vv[1])) {tp.v<-get.tpval(i,j,g,opt,NA);} else {tp.v<-tp.vv;}

if (ntp==1) tmpij<-get2lines(i,j,g,tp.v,opt);

if (ntp==2) tmpij<-get3lines(i,j,g,tp.v,opt);

if (getci) {tt<-cbind(tt,c(tmpij[[1]],getci4tp(i,j,g,opt,tp.v)));} else {tt<-cbind(tt,tmpij[[1]]);}

nnij<-c(nnij,tmpij[[2]])

}

nn<-rbind(nn,nnij)

}

tt<-rbind(c(blbl,blvb_),tt)

w<-c(w,paste("</br>For outcome:",yb[i]))

w<-c(w,paste("</br>For Exposure:",xb[j]))

w<-c(w,"</br><table border=3>", mat2htmltable(tt), "</table>")

}

}

**The relevant code for threshold effect analysis between DII and Constipation in BMI subgroups using EmpowerStats version 3.0 (http://www.empowerstats.net/analysis).**

Sys.setlocale("LC_TIME", "C")

library(doBy,lib.loc=R.LibLocation)

library(plotrix,lib.loc=R.LibLocation)

library(stringi,lib.loc=R.LibLocation)

library(stringr,lib.loc=R.LibLocation)

library(survival,lib.loc=R.LibLocation)

library(rms,lib.loc=R.LibLocation)

library(nnet,lib.loc=R.LibLocation)

library(car,lib.loc=R.LibLocation)

library(mgcv,lib.loc=R.LibLocation)

pdfwd<-6; pdfht<-6

setwd("C:/Users/79156/Desktop/nhances2005-2010/PROJ7_1_tbl")

load("C:/Users/79156/Desktop/nhances2005-2010/bianmi_SZ.Rdata")

if (length(which(ls()=="EmpowerStatsR"))==0) EmpowerStatsR<-get(ls()[1])

names(EmpowerStatsR)<-toupper(names(EmpowerStatsR))

#--#

vname<-c("_N_","_STAT_","_TOTAL_","SEQN","WEIGHT","GENDER","GENDER.1","GENDER.2")

vlabel<-c("样本量(%)","统计量","合计","SEQN","WEIGHT","GENDER"," 1"," 2")

vname<-c(vname,"YEAR","RACE","RACE.1","RACE.2","RACE.3","RACE.4","RACE.5")

vlabel<-c(vlabel,"YEAR","RACE"," 1"," 2"," 3"," 4"," 5")

vname<-c(vname,"BOWEL","BOWEL.0","BOWEL.1","CANCER","CANCER.0","CANCER.1")

vlabel<-c(vlabel,"BOWEL"," 0"," 1","CANCER"," 0"," 1")

vname<-c(vname,"ALBUMIN","ALT","AST","ALKALINE_PHOSPHATASE")

vlabel<-c(vlabel,"ALBUMIN","ALT","AST","ALKALINE_PHOSPHATASE")

vname<-c(vname,"CHOLESTEROL","GST","TOTAL.PROTEIN","TRIGLYCERIDE")

vlabel<-c(vlabel,"CHOLESTEROL","GST","TOTAL.PROTEIN","TRIGLYCERIDE")

vname<-c(vname,"GLOBULIN","WBC","LYMPHOCYTE","MONOCYTE","NEUTROPHIL")

vlabel<-c(vlabel,"GLOBULIN","WBC","LYMPHOCYTE","MONOCYTE","NEUTROPHIL")

vname<-c(vname,"EOSINOPHIL","RBC","HB","PLT","CRP","INFLAMMATORY")

vlabel<-c(vlabel,"EOSINOPHIL","RBC","HB","PLT","CRP","INFLAMMATORY")

vname<-c(vname,"SOMKING","SOMKING.0","SOMKING.1","SOMKING.2")

vlabel<-c(vlabel,"SOMKING"," 0"," 1"," 2")

vname<-c(vname,"DIET","DII","BMI","BMI.1","BMI.2","BMI.3")

vlabel<-c(vlabel,"DIET","DII","BMI"," 1"," 2"," 3")

vname<-c(vname,"ALBUMIN_QUANTILE","ALBUMIN_QUANTILE.1","ALBUMIN_QUANTILE.2","ALBUMIN_QUANTILE.3","ALBUMIN_QUANTILE.4")

vlabel<-c(vlabel,"ALBUMIN_QUANTILE"," 1"," 2"," 3"," 4")

vname<-c(vname,"ALT_QUANTILE","ALT_QUANTILE.1","ALT_QUANTILE.2","ALT_QUANTILE.3","ALT_QUANTILE.4")

vlabel<-c(vlabel,"ALT_QUANTILE"," 1"," 2"," 3"," 4")

vname<-c(vname,"ALKALINE_PHOSPHATASE_QUANTILE","ALKALINE_PHOSPHATASE_QUANTILE.1","ALKALINE_PHOSPHATASE_QUANTILE.2","ALKALINE_PHOSPHATASE_QUANTILE.3","ALKALINE_PHOSPHATASE_QUANTILE.4")

vlabel<-c(vlabel,"ALKALINE_PHOSPHATASE_QUANTILE"," 1"," 2"," 3"," 4")

vname<-c(vname,"GLOBULIN_QUANTILE","GLOBULIN_QUANTILE.1","GLOBULIN_QUANTILE.2","GLOBULIN_QUANTILE.3","GLOBULIN_QUANTILE.4")

vlabel<-c(vlabel,"GLOBULIN_QUANTILE"," 1"," 2"," 3"," 4")

vname<-c(vname,"WBC_QUANTILE","WBC_QUANTILE.1","WBC_QUANTILE.2","WBC_QUANTILE.3","WBC_QUANTILE.4")

vlabel<-c(vlabel,"WBC_QUANTILE"," 1"," 2"," 3"," 4")

vname<-c(vname,"NEUTROPHIL_QUANTILE","NEUTROPHIL_QUANTILE.1","NEUTROPHIL_QUANTILE.2","NEUTROPHIL_QUANTILE.3","NEUTROPHIL_QUANTILE.4")

vlabel<-c(vlabel,"NEUTROPHIL_QUANTILE"," 1"," 2"," 3"," 4")

vname<-c(vname,"RBC_QUANTILE","RBC_QUANTILE.1","RBC_QUANTILE.2","RBC_QUANTILE.3","RBC_QUANTILE.4")

vlabel<-c(vlabel,"RBC_QUANTILE"," 1"," 2"," 3"," 4")

vname<-c(vname,"HB_QUANTILE","HB_QUANTILE.1","HB_QUANTILE.2","HB_QUANTILE.3","HB_QUANTILE.4")

vlabel<-c(vlabel,"HB_QUANTILE"," 1"," 2"," 3"," 4")

vname<-c(vname,"PLT_QUANTILE","PLT_QUANTILE.1","PLT_QUANTILE.2","PLT_QUANTILE.3","PLT_QUANTILE.4")

vlabel<-c(vlabel,"PLT_QUANTILE"," 1"," 2"," 3"," 4")

vname<-c(vname,"CRP_QUANTILE","CRP_QUANTILE.1","CRP_QUANTILE.2","CRP_QUANTILE.3","CRP_QUANTILE.4")

vlabel<-c(vlabel,"CRP_QUANTILE"," 1"," 2"," 3"," 4")

vname<-c(vname,"AAPR","AAPR.1","AAPR.2","AAPR.3","AAPR.4")

vlabel<-c(vlabel,"AAPR"," 1"," 2"," 3"," 4")

vname<-c(vname,"NLR","NLR.1","NLR.2","NLR.3","NLR.4","PNLR","PNLR.1","PNLR.2","PNLR.3","PNLR.4")

vlabel<-c(vlabel,"NLR"," 1"," 2"," 3"," 4","PNLR"," 1"," 2"," 3"," 4")

vname<-c(vname,"LMR","LMR.1","LMR.2","LMR.3","LMR.4","LCR","LCR.1","LCR.2","LCR.3","LCR.4")

vlabel<-c(vlabel,"LMR"," 1"," 2"," 3"," 4","LCR"," 1"," 2"," 3"," 4")

vname<-c(vname,"PLR","PLR.1","PLR.2","PLR.3","PLR.4","IBI","IBI.1","IBI.2","IBI.3","IBI.4")

vlabel<-c(vlabel,"PLR"," 1"," 2"," 3"," 4","IBI"," 1"," 2"," 3"," 4")

slt.vname<-c()

library(MASS,lib.loc=R.LibLocation)

library(gdata,lib.loc=R.LibLocation)

library(geepack,lib.loc=R.LibLocation)

library(mgcv,lib.loc=R.LibLocation)

ofname<-"PROJ7_1_tbl";

WD<-EmpowerStatsR; wd.subset="";

svy.DSN.YN <- FALSE;

weights<-1;weights.var <- NA;

WD<-cbind(WD,weights); WD<-WD[!is.na(weights),];

title<-"阈值效应分析";

attach(WD)

subjvname<-NA;

yv<-cbind(BOWEL);

yvname<-c('BOWEL');

yvar<-c('BOWEL');

ydist<-c('binomial');

ylink<-c('logit');

ylv<-c(2);

xv<-cbind(DII);

xvname<-c('DII');

xvar<-c('DII');

xlv<-c(0);

sxf<-NA;

svname<-NA; sv<-NA; slv<-NA;

av<-cbind(GENDER,YEAR,RACE,SOMKING,CANCER,ALT,AST,CHOLESTEROL,GST,TRIGLYCERIDE,EOSINOPHIL);

avname<-c('GENDER','YEAR','RACE','SOMKING','CANCER','ALT','AST','CHOLESTEROL','GST','TRIGLYCERIDE','EOSINOPHIL');

if (!is.na(avname[1])) avlbl<-vlabel[match(avname, vname)];

nadj<-length(avname);alv<-c(2,0,5,3,2,0,0,0,0,0,0);

saf<-c(NA,0,0,0,0,0,0,0,0,0,0,0)[-1];

timev<-NA; timevname<-NA;

bv<-BMI;bvar<-"BMI";bvname<-"BMI";

colv<-NA; colvname<-NA;

v.start<-NA; vname.start<-NA;

v.stop<-NA; vname.stop<-NA;

par1<-"自动寻找最佳拐点";dec<-3;parm<-c(NA, NA, 1,NA, 0);

if (!exists("pdfwd")) pdfwd<-6;

if (!exists("pdfht")) pdfht<-6;

##R package## MASS gdata geepack mgcv ##R package##;

pvformat<-function(p,dec) {

pp <- sprintf(paste("%.",dec,"f",sep=""),as.numeric(p))

if (is.matrix(p)) {pp<-matrix(pp, nrow=nrow(p)); colnames(pp)<-colnames(p);rownames(pp)<-rownames(p);}

lw <- paste("<",substr("0.00000000000",1,dec+1),"1",sep="");

pp[as.numeric(p)<(1/10^dec)]<-lw

return(pp)

}

numfmt<-function(p,dec) {

if (is.list(p)) p<-as.matrix(p)

pp <- sprintf(paste("%.",dec,"f",sep=""),as.numeric(p))

if (is.matrix(p)) {pp<-matrix(pp, nrow=nrow(p));colnames(pp)<-colnames(p);rownames(pp)<-rownames(p);}

pp[as.numeric(p)>10000000]<- "inf."

pp[is.na(p) | gsub(" ","",p)==""]<- ""

pp[p=="-Inf"]<-"-Inf"

pp[p=="Inf"]<-"Inf"

return(pp)

}

mat2htmltable<-function(mat) {

t1<- apply(mat,1,function(z) paste(z,collapse="</td><td>"))

t2<- paste("<tr><td>",t1,"</td></tr>")

return(paste(t2,collapse=" "))

}

setgam<-function(fml,yi,wdtmp) {

if (ydist[yi]=="") ydist[yi]<-"gaussian"

if (ydist[yi]=="exact") ydist[yi]<-"binomial"

if (ydist[yi]=="breslow") ydist[yi]<-"binomial"

if (ydist[yi]=="gaussian") mdl<-try(gam(formula(fml),weights=wdtmp$weights,data=wdtmp, family=gaussian(link="identity")))

if (ydist[yi]=="binomial") mdl<-try(gam(formula(fml),weights=wdtmp$weights,data=wdtmp, family=binomial(link="logit")))

if (ydist[yi]=="poisson") mdl<-try(gam(formula(fml),weights=wdtmp$weights,data=wdtmp, family=poisson(link="log")))

if (ydist[yi]=="gamma") mdl<-try(gam(formula(fml),weights=wdtmp$weights,data=wdtmp, family=Gamma(link="inverse")))

if (ydist[yi]=="negbin") mdl<-try(gam(formula(fml),weights=wdtmp$weights,data=wdtmp, family=negbin(c(1,10), link="log")))

return(mdl)

}

setgee<-function(fml,yi, wdtmp) {

if (ydist[yi]=="") ydist[yi]<-"gaussian"

if (ydist[yi]=="exact") ydist[yi]<-"binomial"

if (ydist[yi]=="breslow") ydist[yi]<-"binomial"

if (ydist[yi]=="gaussian") md<-try(geeglm(formula(fml),id=wdtmp[,subjvname],corstr=gee.TYPE,family="gaussian",weights=wdtmp$weights,data=wdtmp))

if (ydist[yi]=="binomial") md<-try(geeglm(formula(fml),id=wdtmp[,subjvname],corstr=gee.TYPE,family="binomial",weights=wdtmp$weights,data=wdtmp))

if (ydist[yi]=="poisson") md<-try(geeglm(formula(fml),id=wdtmp[,subjvname],corstr=gee.TYPE,family="poisson",weights=wdtmp$weights,data=wdtmp))

if (ydist[yi]=="gamma") md<-try(geeglm(formula(fml),id=wdtmp[,subjvname],corstr=gee.TYPE,family="Gamma",weights=wdtmp$weights,data=wdtmp))

if (ydist[yi]=="negbin") md<-try(geeglm.nb(formula(fml),id=wdtmp[,subjvname],corstr=gee.TYPE,weights=wdtmp$weights,data=wdtmp))

return(md)

}

setglm<-function(fml,yi, wdtmp) {

if (ydist[yi]=="") ydist[yi]<-"gaussian"

if (ydist[yi]=="exact") ydist[yi]<-"binomial"

if (ydist[yi]=="breslow") ydist[yi]<-"binomial"

if (ydist[yi]=="gaussian") md<-try(glm(formula(fml),family="gaussian",weights=wdtmp$weights,data=wdtmp))

if (ydist[yi]=="binomial") md<-try(glm(formula(fml),family="binomial",weights=wdtmp$weights,data=wdtmp))

if (ydist[yi]=="poisson") md<-try(glm(formula(fml),family="poisson",weights=wdtmp$weights,data=wdtmp))

if (ydist[yi]=="gamma") md<-try(glm(formula(fml),family="Gamma",weights=wdtmp$weights,data=wdtmp))

if (ydist[yi]=="negbin") md<-try(glm.nb(formula(fml),weights=wdtmp$weights,data=wdtmp))

return(md)

}

mdl2oo<-function(mdl, xxname, opt) {

if (is.na(mdl[[1]][1])) return(rep(" ",times=length(xxname)))

if (substr(mdl[[1]][1],1,5)=="Error") return(rep(" ",times=length(xxname)))

decp<-dec+2; if (decp>4) decp<-4

gs<-summary(mdl); print(mdl$formula); print(gs)

if (opt=="gam") {gsparm <- gs$p.table; } else {gsparm <- gs$coefficients;}

gsp<-gsparm[match(xxname,rownames(gsparm)),]

if (length(xxname)==1) {beta<-gsp[1]; se<-gsp[2]; pv<-gsp[4];

} else {beta<-gsp[,1]; se<-gsp[,2]; pv<-gsp[,4]; }

ci1<- beta-1.96*se; ci2<- beta+1.96*se

pvx<-substr(rep("****",length(pv)),1,(pv<=0.05)+(pv<=0.01)+(pv<=0.001))

if (colprn==3) {pvv<-pvx;} else {pvv<-pvformat(pv,decp);}

if ((colprn!=2) & (gs$family[[2]]=="log" | gs$family[[2]]=="logit")) {

o1<-paste(numfmt(exp(beta),dec)," (",numfmt(exp(ci1),dec),", ",numfmt(exp(ci2),dec),")",sep="")

} else {

if (colprn<3) {o1<-paste(numfmt(beta,dec), " (",numfmt(ci1,dec),", ",numfmt(ci2,dec),")",sep="")

} else {o1<-paste(numfmt(beta,dec), "+",numfmt(se,dec),sep="");}

}

o1<-paste(o1,pvv); o1[is.na(beta)]<-NA

return(o1)

}

removeNA<-function(i,j,wdf) {

vvv<-c(yvname[i],xvname[j],avname,subjvname,bvar,vname.start,vname.stop,timevname);

vvv<-vvv[!is.na(vvv)]; vvv<-vvv[vvv>" "]

tmp<-is.na(wdf[,vvv]);

return(wdf[apply(tmp,1,sum)==0,])

}

get.tpval<-function(i,j,g,opt,wdtmp, tppmin=NA, tppmax=NA) {

if (is.na(wdtmp)) {

if (is.na(g)) {wdtmp<-removeNA(i,j,WD);

} else if (g<nblv) {wdtmp0<-WD[WD[,bvar]==blv[g],]; wdtmp<-removeNA(i,j,wdtmp0);

} else {wdtmp<-removeNA(i,j,WD); }

}

if (is.na(g)) {fmladj1<-fmladj;

} else if (g<nblv) {fmladj1<-fmladj;

} else {fmladj1<-paste(fmladj,"+factor(",bvar,")",sep="");}

xTMP <- wdtmp[,xvname[j]]

tmp.ss<-seq(0.05,0.95,0.05)

tp<-quantile(xTMP,probs=tmp.ss,na.rm=TRUE)

tmp.llk<-rep(NA,length(tmp.ss))

fml<-paste(yvname[i],"~",xvname[j],"+tmp.X",fmladj1)

if (!is.na(tppmin) & !is.na(tppmax)) {

tp2.min = tppmin; tp2.max = tppmax;

} else {

for (k in (1:length(tmp.ss))) {

tmp.X<-(xTMP > tp[k])*(xTMP-tp[k]); wdtmp1<-cbind(wdtmp,tmp.X)

if (opt=="glm" | opt=="gee") tmp.mdl<-setglm(fml, i, wdtmp1);

if (opt=="gam") tmp.mdl<-setgam(fml, i, wdtmp1);

tmp.llk[k]<-logLik(tmp.mdl)

rm(wdtmp1, tmp.X)

}

tp1<-tmp.ss[which.max(tmp.llk)]

tp2.min = tp1 - 0.04

tp2.max = tp1 + 0.04

if (tp2.min<0.05) {tp2.min=0.05}

if (tp2.max>0.95) {tp2.max=0.95}

}

tp.pctlrange<-quantile(xTMP,probs=c(tp2.min,tp2.max),na.rm=TRUE)

tp.range<-unique(xTMP[xTMP>tp.pctlrange[1] & xTMP<tp.pctlrange[2]])

while (length(tp.range)>5) {

tmp.pct3<-quantile(tp.range,probs=c(0,0.25,0.5,0.75,1),type=3)

tmp.llk3<-rep(NA,3)

for (k in (2:4)) {

tmp.X<-(xTMP>tmp.pct3[k])*(xTMP-tmp.pct3[k]); wdtmp1<-cbind(wdtmp,tmp.X)

if (opt=="glm" | opt=="gee") tmp.mdl<-setglm(fml, i, wdtmp1);

if (opt=="gam") tmp.mdl<-setgam(fml, i, wdtmp1);

tmp.llk3[k-1]<-logLik(tmp.mdl)

rm(wdtmp1, tmp.X)

}

tmp.min3<-which.max(tmp.llk3)

tp.range<-tp.range[tp.range>=tmp.pct3[tmp.min3] & tp.range<=tmp.pct3[tmp.min3+2]]

}

if (length(tp.range)>0) {

if (length(tp.range)==1) {tp.val=tp.range[1];} else {

tmp.llk<-rep(NA,length(tp.range))

for (k in (1:length(tp.range))) {

tmp.X<-(xTMP>tp.range[k])*(xTMP-tp.range[k]); wdtmp1<-cbind(wdtmp,tmp.X)

if (opt=="glm" | opt=="gee") tmp.mdl<-setglm(fml, i, wdtmp1);

if (opt=="gam") tmp.mdl<-setgam(fml, i, wdtmp1);

tmp.llk[k]<-logLik(tmp.mdl)

rm(wdtmp1, tmp.X)

}

tp.val<-tp.range[which.max(tmp.llk)]

}

} else { tp.val<-tp.pctlrange[1];}

return(round(tp.val,dec));

}

get2lines<-function(i,j,g,tp.value,opt) {

if (is.na(g)) {fmladj1<-fmladj;wdtmp<-removeNA(i,j,WD);

} else if (g<nblv) {fmladj1<-fmladj;wdtmp0<-WD[WD[,bvar]==blv[g],];wdtmp<-removeNA(i,j,wdtmp0);

} else {fmladj1<-paste(fmladj,"+factor(",bvar,")",sep="");wdtmp<-removeNA(i,j,WD);}

xTMP<-wdtmp[,xvname[j]]

tmp.X1<-(xTMP<=tp.value)*(xTMP-tp.value)

tmp.X2<-(xTMP> tp.value)*(xTMP-tp.value)

wdtmp1<-cbind(wdtmp,xTMP,tmp.X1,tmp.X2)

fml0<-paste(yvname[i],"~xTMP+tmp.X2",fmladj1)

fml1<-paste(yvname[i],"~tmp.X1+tmp.X2",fmladj1)

fml2<-paste(yvname[i],"~xTMP",fmladj1)

fmlp<-paste(yvname[i],"~xTMP+tmp.X2")

tmpn<-nrow(wdtmp)

if (opt=="glm") {

tmp.mdl0<-setglm(fml0,i,wdtmp1); tmp.mdl1<-setglm(fml1,i,wdtmp1)

tmp.mdl2<-setglm(fml2,i,wdtmp1); tmp.mdlp<-setglm(fmlp,i,wdtmp1)

}

if (opt=="gam") {

tmp.mdl0<-setgam(fml0,i,wdtmp1); tmp.mdl1<-setgam(fml1,i,wdtmp1)

tmp.mdl2<-setgam(fml2,i,wdtmp1); tmp.mdlp<-setgam(fmlp,i,wdtmp1)

}

if (opt=="gee") {

tmp.mdl0<-setgee(fml0,i,wdtmp1); tmp.mdl1<-setgee(fml1,i,wdtmp1)

tmp.mdl2<-setgee(fml2,i,wdtmp1); tmp.mdlp<-setglm(fmlp,i,wdtmp1)

}

pd<-predict(tmp.mdlp,data.frame(xTMP=tp.value,tmp.X2=0), se.fit=TRUE)

prd<-paste(numfmt(pd$fit,dec)," (",numfmt(pd$fit-1.96*pd$se.fit,dec),", ", numfmt(pd$fit+1.96*pd$se.fit, dec),")",sep="")

m2<-mdl2oo(tmp.mdl2,"xTMP",opt)

m1<-mdl2oo(tmp.mdl1,c("tmp.X1","tmp.X2"),opt)

m0<-mdl2oo(tmp.mdl0,"tmp.X2",opt)

if (opt=="gee") {

plrt<-try(anova(tmp.mdl0,tmp.mdl2)$"P(>|Chi|)",TRUE)

plrt<-ifelse((plrt<="9" && plrt>="0"), pvformat(plrt,3),"-")

} else {plrt<- pvformat(1-pchisq(2*(logLik(tmp.mdl0)[1]-logLik(tmp.mdl2)[1]),1),3);}

oo<-list(c("",m2,"",tp.value,m1,m0,prd,plrt),tmpn)

return(oo)

}

get3lines<-function(i,j,g,tp.value,opt) {

if (is.na(g)) {fmladj1<-fmladj;wdtmp<-removeNA(i,j,WD);

} else if (g<nblv) {fmladj1<-fmladj;wdtmp0<-WD[WD[,bvar]==blv[g],];wdtmp<-removeNA(i,j,wdtmp0);

} else {fmladj1<-paste(fmladj,"+factor(",bvar,")",sep="");wdtmp<-removeNA(i,j,WD);}

xTMP<-wdtmp[,xvname[j]]; tp1<-tp.value[1]; tp2<-tp.value[2]

tmp.X1<- (xTMP< tp1)*(xTMP-tp1)

tmp.X2<-((xTMP>=tp1) & (xTMP<=tp2))*(xTMP-tp1)

tmp.X3<- (xTMP> tp2)*(xTMP-tp2)

tmp.B1<- (xTMP>tp2)

wdtmp1<-cbind(wdtmp,xTMP,tmp.X1,tmp.X2,tmp.X3,tmp.B1)

fml0<-paste(yvname[i],"~xTMP+tmp.X1+tmp.X3+tmp.B1",fmladj1)

fml1<-paste(yvname[i],"~tmp.X1+tmp.X2+tmp.X3+tmp.B1",fmladj1)

fml2<-paste(yvname[i],"~xTMP",fmladj1)

tmpn<-nrow(wdtmp)

if (opt=="glm") {

tmp.mdl0<-setglm(fml0,i,wdtmp1); tmp.mdl1<-setglm(fml1,i,wdtmp1); tmp.mdl2<-setglm(fml2,i,wdtmp1);

}

if (opt=="gam") {

tmp.mdl0<-setgam(fml0,i,wdtmp1); tmp.mdl1<-setgam(fml1,i,wdtmp1); tmp.mdl2<-setgam(fml2,i,wdtmp1);

}

if (opt=="gee") {

tmp.mdl0<-setgee(fml0,i,wdtmp1); tmp.mdl1<-setgee(fml1,i,wdtmp1); tmp.mdl2<-setgee(fml2,i,wdtmp1);

}

m2<-mdl2oo(tmp.mdl2,"xTMP",opt)

m1<-mdl2oo(tmp.mdl1,c("tmp.X1","tmp.X2","tmp.X3"),opt)

m0<-mdl2oo(tmp.mdl0,c("tmp.X1","tmp.X3"),opt)

if (opt=="gee") {

plrt<-try(anova(tmp.mdl0,tmp.mdl2)$"P(>|Chi|)",TRUE)

plrt<-ifelse((plrt<="9" && plrt>="0"), pvformat(plrt,3),"-")

} else {plrt<- pvformat(1-pchisq(2*(logLik(tmp.mdl0)[1]-logLik(tmp.mdl2)[1]),1),3);}

oo<-list(c("",m2,"",paste(tp.value,collapse=", "),m1,m0,plrt),tmpn)

return(oo)

}

getci4tp<-function(i,j,g,opt,tp0=NA) {

set.seed(123456)

if (is.na(g)) {wdt<-removeNA(i,j,WD);

} else if (g<nblv) {wdt<-WD[WD[,bvar]==blv[g],];wdt<-removeNA(i,j,wdt);

} else {wdt<-removeNA(i,j,WD);}

nnwd<-nrow(wdt); tp.vv<-rep(NA,1000)

if (!is.na(tp0)) {

tpp0 = sum(wdt[,xvname[j]] < tp0)/length(wdt[,xvname[j]])

tppmin = max(tpp0 - 0.09, 0.05)

tppmax = min(tpp0 + 0.09, 0.95)

} else {

tppmin = NA; tppmax = NA

}

for (s in (1:1000)) {

WDi<-wdt[sample(1:nnwd,nnwd,replace=T),]

tp.vv[s]<-get.tpval(i, j, NA, opt, WDi, tppmin, tppmax); rm(WDi)

}

tpci<-paste(quantile(tp.vv,probs=c(0.025,0.975)),collapse=", ")

return(tpci);

}

vlabelN<-(substr(vlabel,1,1)==" ");

vlabelZ<-vlabel[vlabelN];vlabelV<-vlabel[!vlabelN]

vnameV<-vname[!vlabelN];vnameZ<-vname[vlabelN];

allvname<-c(yvname,xvname,bvar,avname,subjvname,vname.start,vname.stop,timevname,"weights");

allvname<-allvname[!is.na(allvname)]

WD<-WD[,allvname];

w<-c("<html><head>","<meta http-equiv=\"Content-Type\" content=\"text/html\" charset=\"gb2312\" /></head><body>")

if (!is.na(avname[1])) {

if (sum((saf=="s" | saf=="S") & alv>0)>0) w<-c(w,"</br>Spline smoothing only applies for continuous variables")

if (!is.na(subjvname)) saf<-rep(0,length(saf))

}

if (sum(xlv>0)>0) w<-c(w,"Categorical exposure variables were ignored")

xvname<-xvname[xlv==0];

if (!is.na(subjvname)) WD<-WD[order(WD[,subjvname]),];

fmladj<-""; avb=""; smoothav<-0;

if (!is.na(avname[1])) {

avb<-vlabelV[match(avname,vnameV)];

avname_ <- avname

smoothavi<-((saf=="s" | saf=="S") & alv==0)

smoothav<-sum(smoothavi)

avname_[smoothavi]<-paste("s(",avname[smoothavi],")",sep="")

avb1<-avb

avb1[smoothavi]<-paste(avb[smoothavi],"(Smooth)",sep="")

avname_[alv>0]<-paste("factor(",avname[alv>0],")",sep="")

fmladj<-paste("+",paste(avname_,collapse="+"))

}

if (is.na(bvar)) {

blvb<-"N"; blvb_<-"N"; nblv<-1; blbl<-"";

} else {

blbl<-vlabelV[match(bvar,vnameV)]; if (is.na(blbl)) blbl<-bvar;

blv<-levels(factor(WD[,bvar])); nblv<-length(blv)+1

blvb_<-vlabelZ[match(paste(bvar,".",blv,sep=""),vnameZ)];

blvb_[is.na(blvb_)]<-blv[is.na(blvb_)];

blvb<-c(paste(blbl,blvb_,sep="="),"Total");

blvb_<-c(blvb_,"Total")

WD<-WD[!is.na(WD[,bvar]),]

}

ny=length(yvname); nx=length(xvname);

xb<-vlabelV[match(xvname,vnameV)]; xb[is.na(xb)]<-xvname[is.na(xb)]

yb<-vlabelV[match(yvname,vnameV)]; yb[is.na(yb)]<-yvname[is.na(yb)]

opt<-ifelse(!is.na(subjvname), "gee", ifelse(smoothav>0, "gam", "glm")) ;

colprn<-parm[3]

if (is.na(par1)) par1<-"";

if (is.numeric(par1)) {tp.vv<-par1;

} else {

tmp<-as.numeric(strsplit(par1," ")[[1]]); tp.vv<-c(tmp[!is.na(tmp)],NA)

}

prn<-ifelse(!is.na(bvar), "S", ifelse(nx>1 & ny==1, "X", "Y"));

if (length(tp.vv)>2) tp.vv<-tp.vv[1:2]

ntp<-length(tp.vv);

getci<-FALSE

prnopt<-c("β (95%CI) Pvalue / OR (95%CI) Pvalue", "β (95%CI) Pvalue", "β+se / OR (95%CI) *P<0.05 **P<0.01 ***P<0.001")

if (ntp==1) {

cc0<-c("模型 I","&nbsp&nbsp一条直线效应");

cc0<-c(cc0,"模型 II","&nbsp&nbsp折点(K)","&nbsp&nbsp &lt K 段效应 1","&nbsp&nbsp &gt K 段效应 2","&nbsp&nbsp 2与1的效应差")

cc0<-c(cc0,"&nbsp&nbsp折点处方程预测值")

if (is.na(tp.vv[1]) & !is.na(parm[1])) getci<-TRUE;

} else {

cc0<-c("模型 I","&nbsp&nbsp一条直线效应");

cc0<-c(cc0,"模型 II","&nbsp&nbsp折点(K1,K2)","&nbsp&nbsp &lt K1 段效应 1","&nbsp&nbsp K1-K2 段效应 2","&nbsp&nbsp &gt K2 段效应 3")

cc0<-c(cc0,"&nbsp&nbsp 1与2的效应差","&nbsp&nbsp 3与2的效应差")

}

if (opt=="gee") {cc0<-c(cc0,"ANOVA 两模型比较");} else {cc0<-c(cc0,"对数似然比检验");}

if (getci) cc0<-c(cc0,"折点的95可信区间");

sink(paste(ofname,".lst",sep=""))

w<-c(w,paste("<h2>", title, "</h2>"))

nn<-c("Outcome","Exposure",blvb);

if (prn=="Y") {

for (j in 1:nx) {

tt<-cc0;

for (i in 1:ny) {

if (is.na(tp.vv[1])) {tp.v<-get.tpval(i,j,NA,opt,NA);} else {tp.v<-tp.vv;}

if (ntp==1) tmpij<-get2lines(i,j,NA,tp.v,opt);

if (ntp==2) tmpij<-get3lines(i,j,NA,tp.v,opt);

if (getci) {tt<-cbind(tt,c(tmpij[[1]],getci4tp(i,j,NA,opt,tp.v)));} else {tt<-cbind(tt,tmpij[[1]]);}

nn<-rbind(nn,c(yb[i],xb[j],tmpij[[2]]))

}

tt<-rbind(c("Outcome: ",yb),tt)

w<-c(w,paste("</br>For exposure:",xb[j]))

w<-c(w,"</br><table border=3>", mat2htmltable(tt), "</table>")

}

}

if (prn=="X") {

for (i in 1:ny) {

tt<-cc0;

for (j in 1:nx) {

if (is.na(tp.vv[1])) {tp.v<-get.tpval(i,j,NA,opt,NA);} else {tp.v<-tp.vv;}

if (ntp==1) tmpij<-get2lines(i,j,NA,tp.v,opt);

if (ntp==2) tmpij<-get3lines(i,j,NA,tp.v,opt);

if (getci) {tt<-cbind(tt,c(tmpij[[1]],getci4tp(i,j,NA,opt,tp.v)));} else {tt<-cbind(tt,tmpij[[1]]);}

nn<-rbind(nn,c(yb[i],xb[j],tmpij[[2]]))

}

tt<-rbind(c("Exposure: ",xb),tt)

w<-c(w,paste("</br>For outcome:",yb[i]))

w<-c(w,"</br><table border=3>", mat2htmltable(tt), "</table>")

}

}

if (prn=="S") {

for (i in 1:ny) {

tt<-cc0;

for (j in 1:nx) {

nnij<-c(yb[i],xb[j])

for (g in 1:nblv) {

if (is.na(tp.vv[1])) {tp.v<-get.tpval(i,j,g,opt,NA);} else {tp.v<-tp.vv;}

if (ntp==1) tmpij<-get2lines(i,j,g,tp.v,opt);

if (ntp==2) tmpij<-get3lines(i,j,g,tp.v,opt);

if (getci) {tt<-cbind(tt,c(tmpij[[1]],getci4tp(i,j,g,opt,tp.v)));} else {tt<-cbind(tt,tmpij[[1]]);}

nnij<-c(nnij,tmpij[[2]])

}

nn<-rbind(nn,nnij)

}

tt<-rbind(c(blbl,blvb_),tt)

w<-c(w,paste("</br>For outcome:",yb[i]))

w<-c(w,paste("</br>For Exposure:",xb[j]))

w<-c(w,"</br><table border=3>", mat2htmltable(tt), "</table>")

}

}

**The relevant code for threshold effect analysis between DII and Constipation in IBI subgroups using EmpowerStats version 3.0 (http://www.empowerstats.net/analysis).**

Sys.setlocale("LC_TIME", "C")

library(doBy,lib.loc=R.LibLocation)

library(plotrix,lib.loc=R.LibLocation)

library(stringi,lib.loc=R.LibLocation)

library(stringr,lib.loc=R.LibLocation)

library(survival,lib.loc=R.LibLocation)

library(rms,lib.loc=R.LibLocation)

library(nnet,lib.loc=R.LibLocation)

library(car,lib.loc=R.LibLocation)

library(mgcv,lib.loc=R.LibLocation)

pdfwd<-6; pdfht<-6

setwd("C:/Users/79156/Desktop/nhances2005-2010/PROJ7_1_tbl")

load("C:/Users/79156/Desktop/nhances2005-2010/bianmi_SZ.Rdata")

if (length(which(ls()=="EmpowerStatsR"))==0) EmpowerStatsR<-get(ls()[1])

names(EmpowerStatsR)<-toupper(names(EmpowerStatsR))

#--#

vname<-c("_N_","_STAT_","_TOTAL_","SEQN","WEIGHT","GENDER","GENDER.1","GENDER.2")

vlabel<-c("样本量(%)","统计量","合计","SEQN","WEIGHT","GENDER"," 1"," 2")

vname<-c(vname,"YEAR","RACE","RACE.1","RACE.2","RACE.3","RACE.4","RACE.5")

vlabel<-c(vlabel,"YEAR","RACE"," 1"," 2"," 3"," 4"," 5")

vname<-c(vname,"BOWEL","BOWEL.0","BOWEL.1","CANCER","CANCER.0","CANCER.1")

vlabel<-c(vlabel,"BOWEL"," 0"," 1","CANCER"," 0"," 1")

vname<-c(vname,"ALBUMIN","ALT","AST","ALKALINE_PHOSPHATASE")

vlabel<-c(vlabel,"ALBUMIN","ALT","AST","ALKALINE_PHOSPHATASE")

vname<-c(vname,"CHOLESTEROL","GST","TOTAL.PROTEIN","TRIGLYCERIDE")

vlabel<-c(vlabel,"CHOLESTEROL","GST","TOTAL.PROTEIN","TRIGLYCERIDE")

vname<-c(vname,"GLOBULIN","WBC","LYMPHOCYTE","MONOCYTE","NEUTROPHIL")

vlabel<-c(vlabel,"GLOBULIN","WBC","LYMPHOCYTE","MONOCYTE","NEUTROPHIL")

vname<-c(vname,"EOSINOPHIL","RBC","HB","PLT","CRP","INFLAMMATORY")

vlabel<-c(vlabel,"EOSINOPHIL","RBC","HB","PLT","CRP","INFLAMMATORY")

vname<-c(vname,"SOMKING","SOMKING.0","SOMKING.1","SOMKING.2")

vlabel<-c(vlabel,"SOMKING"," 0"," 1"," 2")

vname<-c(vname,"DIET","DII","BMI","BMI.1","BMI.2","BMI.3")

vlabel<-c(vlabel,"DIET","DII","BMI"," 1"," 2"," 3")

vname<-c(vname,"ALBUMIN_QUANTILE","ALBUMIN_QUANTILE.1","ALBUMIN_QUANTILE.2","ALBUMIN_QUANTILE.3","ALBUMIN_QUANTILE.4")

vlabel<-c(vlabel,"ALBUMIN_QUANTILE"," 1"," 2"," 3"," 4")

vname<-c(vname,"ALT_QUANTILE","ALT_QUANTILE.1","ALT_QUANTILE.2","ALT_QUANTILE.3","ALT_QUANTILE.4")

vlabel<-c(vlabel,"ALT_QUANTILE"," 1"," 2"," 3"," 4")

vname<-c(vname,"ALKALINE_PHOSPHATASE_QUANTILE","ALKALINE_PHOSPHATASE_QUANTILE.1","ALKALINE_PHOSPHATASE_QUANTILE.2","ALKALINE_PHOSPHATASE_QUANTILE.3","ALKALINE_PHOSPHATASE_QUANTILE.4")

vlabel<-c(vlabel,"ALKALINE_PHOSPHATASE_QUANTILE"," 1"," 2"," 3"," 4")

vname<-c(vname,"GLOBULIN_QUANTILE","GLOBULIN_QUANTILE.1","GLOBULIN_QUANTILE.2","GLOBULIN_QUANTILE.3","GLOBULIN_QUANTILE.4")

vlabel<-c(vlabel,"GLOBULIN_QUANTILE"," 1"," 2"," 3"," 4")

vname<-c(vname,"WBC_QUANTILE","WBC_QUANTILE.1","WBC_QUANTILE.2","WBC_QUANTILE.3","WBC_QUANTILE.4")

vlabel<-c(vlabel,"WBC_QUANTILE"," 1"," 2"," 3"," 4")

vname<-c(vname,"NEUTROPHIL_QUANTILE","NEUTROPHIL_QUANTILE.1","NEUTROPHIL_QUANTILE.2","NEUTROPHIL_QUANTILE.3","NEUTROPHIL_QUANTILE.4")

vlabel<-c(vlabel,"NEUTROPHIL_QUANTILE"," 1"," 2"," 3"," 4")

vname<-c(vname,"RBC_QUANTILE","RBC_QUANTILE.1","RBC_QUANTILE.2","RBC_QUANTILE.3","RBC_QUANTILE.4")

vlabel<-c(vlabel,"RBC_QUANTILE"," 1"," 2"," 3"," 4")

vname<-c(vname,"HB_QUANTILE","HB_QUANTILE.1","HB_QUANTILE.2","HB_QUANTILE.3","HB_QUANTILE.4")

vlabel<-c(vlabel,"HB_QUANTILE"," 1"," 2"," 3"," 4")

vname<-c(vname,"PLT_QUANTILE","PLT_QUANTILE.1","PLT_QUANTILE.2","PLT_QUANTILE.3","PLT_QUANTILE.4")

vlabel<-c(vlabel,"PLT_QUANTILE"," 1"," 2"," 3"," 4")

vname<-c(vname,"CRP_QUANTILE","CRP_QUANTILE.1","CRP_QUANTILE.2","CRP_QUANTILE.3","CRP_QUANTILE.4")

vlabel<-c(vlabel,"CRP_QUANTILE"," 1"," 2"," 3"," 4")

vname<-c(vname,"AAPR","AAPR.1","AAPR.2","AAPR.3","AAPR.4")

vlabel<-c(vlabel,"AAPR"," 1"," 2"," 3"," 4")

vname<-c(vname,"NLR","NLR.1","NLR.2","NLR.3","NLR.4","PNLR","PNLR.1","PNLR.2","PNLR.3","PNLR.4")

vlabel<-c(vlabel,"NLR"," 1"," 2"," 3"," 4","PNLR"," 1"," 2"," 3"," 4")

vname<-c(vname,"LMR","LMR.1","LMR.2","LMR.3","LMR.4","LCR","LCR.1","LCR.2","LCR.3","LCR.4")

vlabel<-c(vlabel,"LMR"," 1"," 2"," 3"," 4","LCR"," 1"," 2"," 3"," 4")

vname<-c(vname,"PLR","PLR.1","PLR.2","PLR.3","PLR.4","IBI","IBI.1","IBI.2","IBI.3","IBI.4")

vlabel<-c(vlabel,"PLR"," 1"," 2"," 3"," 4","IBI"," 1"," 2"," 3"," 4")

slt.vname<-c()

library(MASS,lib.loc=R.LibLocation)

library(gdata,lib.loc=R.LibLocation)

library(geepack,lib.loc=R.LibLocation)

library(mgcv,lib.loc=R.LibLocation)

ofname<-"PROJ7_1_tbl";

WD<-EmpowerStatsR; wd.subset="";

svy.DSN.YN <- FALSE;

weights<-1;weights.var <- NA;

WD<-cbind(WD,weights); WD<-WD[!is.na(weights),];

title<-"阈值效应分析";

attach(WD)

subjvname<-NA;

yv<-cbind(BOWEL);

yvname<-c('BOWEL');

yvar<-c('BOWEL');

ydist<-c('binomial');

ylink<-c('logit');

ylv<-c(2);

xv<-cbind(DII);

xvname<-c('DII');

xvar<-c('DII');

xlv<-c(0);

sxf<-NA;

svname<-NA; sv<-NA; slv<-NA;

av<-cbind(GENDER,YEAR,RACE,CANCER,ALT,AST,CHOLESTEROL,GST,TRIGLYCERIDE,EOSINOPHIL,SOMKING);

avname<-c('GENDER','YEAR','RACE','CANCER','ALT','AST','CHOLESTEROL','GST','TRIGLYCERIDE','EOSINOPHIL','SOMKING');

if (!is.na(avname[1])) avlbl<-vlabel[match(avname, vname)];

nadj<-length(avname);alv<-c(2,0,5,2,0,0,0,0,0,0,3);

saf<-c(NA,0,0,0,0,0,0,0,0,0,0,0)[-1];

timev<-NA; timevname<-NA;

bv<-IBI;bvar<-"IBI";bvname<-"IBI";

colv<-NA; colvname<-NA;

v.start<-NA; vname.start<-NA;

v.stop<-NA; vname.stop<-NA;

par1<-"自动寻找最佳拐点";dec<-3;parm<-c(NA, NA, 1,NA, 0);

if (!exists("pdfwd")) pdfwd<-6;

if (!exists("pdfht")) pdfht<-6;

##R package## MASS gdata geepack mgcv ##R package##;

pvformat<-function(p,dec) {

pp <- sprintf(paste("%.",dec,"f",sep=""),as.numeric(p))

if (is.matrix(p)) {pp<-matrix(pp, nrow=nrow(p)); colnames(pp)<-colnames(p);rownames(pp)<-rownames(p);}

lw <- paste("<",substr("0.00000000000",1,dec+1),"1",sep="");

pp[as.numeric(p)<(1/10^dec)]<-lw

return(pp)

}

numfmt<-function(p,dec) {

if (is.list(p)) p<-as.matrix(p)

pp <- sprintf(paste("%.",dec,"f",sep=""),as.numeric(p))

if (is.matrix(p)) {pp<-matrix(pp, nrow=nrow(p));colnames(pp)<-colnames(p);rownames(pp)<-rownames(p);}

pp[as.numeric(p)>10000000]<- "inf."

pp[is.na(p) | gsub(" ","",p)==""]<- ""

pp[p=="-Inf"]<-"-Inf"

pp[p=="Inf"]<-"Inf"

return(pp)

}

mat2htmltable<-function(mat) {

t1<- apply(mat,1,function(z) paste(z,collapse="</td><td>"))

t2<- paste("<tr><td>",t1,"</td></tr>")

return(paste(t2,collapse=" "))

}

setgam<-function(fml,yi,wdtmp) {

if (ydist[yi]=="") ydist[yi]<-"gaussian"

if (ydist[yi]=="exact") ydist[yi]<-"binomial"

if (ydist[yi]=="breslow") ydist[yi]<-"binomial"

if (ydist[yi]=="gaussian") mdl<-try(gam(formula(fml),weights=wdtmp$weights,data=wdtmp, family=gaussian(link="identity")))

if (ydist[yi]=="binomial") mdl<-try(gam(formula(fml),weights=wdtmp$weights,data=wdtmp, family=binomial(link="logit")))

if (ydist[yi]=="poisson") mdl<-try(gam(formula(fml),weights=wdtmp$weights,data=wdtmp, family=poisson(link="log")))

if (ydist[yi]=="gamma") mdl<-try(gam(formula(fml),weights=wdtmp$weights,data=wdtmp, family=Gamma(link="inverse")))

if (ydist[yi]=="negbin") mdl<-try(gam(formula(fml),weights=wdtmp$weights,data=wdtmp, family=negbin(c(1,10), link="log")))

return(mdl)

}

setgee<-function(fml,yi, wdtmp) {

if (ydist[yi]=="") ydist[yi]<-"gaussian"

if (ydist[yi]=="exact") ydist[yi]<-"binomial"

if (ydist[yi]=="breslow") ydist[yi]<-"binomial"

if (ydist[yi]=="gaussian") md<-try(geeglm(formula(fml),id=wdtmp[,subjvname],corstr=gee.TYPE,family="gaussian",weights=wdtmp$weights,data=wdtmp))

if (ydist[yi]=="binomial") md<-try(geeglm(formula(fml),id=wdtmp[,subjvname],corstr=gee.TYPE,family="binomial",weights=wdtmp$weights,data=wdtmp))

if (ydist[yi]=="poisson") md<-try(geeglm(formula(fml),id=wdtmp[,subjvname],corstr=gee.TYPE,family="poisson",weights=wdtmp$weights,data=wdtmp))

if (ydist[yi]=="gamma") md<-try(geeglm(formula(fml),id=wdtmp[,subjvname],corstr=gee.TYPE,family="Gamma",weights=wdtmp$weights,data=wdtmp))

if (ydist[yi]=="negbin") md<-try(geeglm.nb(formula(fml),id=wdtmp[,subjvname],corstr=gee.TYPE,weights=wdtmp$weights,data=wdtmp))

return(md)

}

setglm<-function(fml,yi, wdtmp) {

if (ydist[yi]=="") ydist[yi]<-"gaussian"

if (ydist[yi]=="exact") ydist[yi]<-"binomial"

if (ydist[yi]=="breslow") ydist[yi]<-"binomial"

if (ydist[yi]=="gaussian") md<-try(glm(formula(fml),family="gaussian",weights=wdtmp$weights,data=wdtmp))

if (ydist[yi]=="binomial") md<-try(glm(formula(fml),family="binomial",weights=wdtmp$weights,data=wdtmp))

if (ydist[yi]=="poisson") md<-try(glm(formula(fml),family="poisson",weights=wdtmp$weights,data=wdtmp))

if (ydist[yi]=="gamma") md<-try(glm(formula(fml),family="Gamma",weights=wdtmp$weights,data=wdtmp))

if (ydist[yi]=="negbin") md<-try(glm.nb(formula(fml),weights=wdtmp$weights,data=wdtmp))

return(md)

}

mdl2oo<-function(mdl, xxname, opt) {

if (is.na(mdl[[1]][1])) return(rep(" ",times=length(xxname)))

if (substr(mdl[[1]][1],1,5)=="Error") return(rep(" ",times=length(xxname)))

decp<-dec+2; if (decp>4) decp<-4

gs<-summary(mdl); print(mdl$formula); print(gs)

if (opt=="gam") {gsparm <- gs$p.table; } else {gsparm <- gs$coefficients;}

gsp<-gsparm[match(xxname,rownames(gsparm)),]

if (length(xxname)==1) {beta<-gsp[1]; se<-gsp[2]; pv<-gsp[4];

} else {beta<-gsp[,1]; se<-gsp[,2]; pv<-gsp[,4]; }

ci1<- beta-1.96*se; ci2<- beta+1.96*se

pvx<-substr(rep("****",length(pv)),1,(pv<=0.05)+(pv<=0.01)+(pv<=0.001))

if (colprn==3) {pvv<-pvx;} else {pvv<-pvformat(pv,decp);}

if ((colprn!=2) & (gs$family[[2]]=="log" | gs$family[[2]]=="logit")) {

o1<-paste(numfmt(exp(beta),dec)," (",numfmt(exp(ci1),dec),", ",numfmt(exp(ci2),dec),")",sep="")

} else {

if (colprn<3) {o1<-paste(numfmt(beta,dec), " (",numfmt(ci1,dec),", ",numfmt(ci2,dec),")",sep="")

} else {o1<-paste(numfmt(beta,dec), "+",numfmt(se,dec),sep="");}

}

o1<-paste(o1,pvv); o1[is.na(beta)]<-NA

return(o1)

}

removeNA<-function(i,j,wdf) {

vvv<-c(yvname[i],xvname[j],avname,subjvname,bvar,vname.start,vname.stop,timevname);

vvv<-vvv[!is.na(vvv)]; vvv<-vvv[vvv>" "]

tmp<-is.na(wdf[,vvv]);

return(wdf[apply(tmp,1,sum)==0,])

}

get.tpval<-function(i,j,g,opt,wdtmp, tppmin=NA, tppmax=NA) {

if (is.na(wdtmp)) {

if (is.na(g)) {wdtmp<-removeNA(i,j,WD);

} else if (g<nblv) {wdtmp0<-WD[WD[,bvar]==blv[g],]; wdtmp<-removeNA(i,j,wdtmp0);

} else {wdtmp<-removeNA(i,j,WD); }

}

if (is.na(g)) {fmladj1<-fmladj;

} else if (g<nblv) {fmladj1<-fmladj;

} else {fmladj1<-paste(fmladj,"+factor(",bvar,")",sep="");}

xTMP <- wdtmp[,xvname[j]]

tmp.ss<-seq(0.05,0.95,0.05)

tp<-quantile(xTMP,probs=tmp.ss,na.rm=TRUE)

tmp.llk<-rep(NA,length(tmp.ss))

fml<-paste(yvname[i],"~",xvname[j],"+tmp.X",fmladj1)

if (!is.na(tppmin) & !is.na(tppmax)) {

tp2.min = tppmin; tp2.max = tppmax;

} else {

for (k in (1:length(tmp.ss))) {

tmp.X<-(xTMP > tp[k])*(xTMP-tp[k]); wdtmp1<-cbind(wdtmp,tmp.X)

if (opt=="glm" | opt=="gee") tmp.mdl<-setglm(fml, i, wdtmp1);

if (opt=="gam") tmp.mdl<-setgam(fml, i, wdtmp1);

tmp.llk[k]<-logLik(tmp.mdl)

rm(wdtmp1, tmp.X)

}

tp1<-tmp.ss[which.max(tmp.llk)]

tp2.min = tp1 - 0.04

tp2.max = tp1 + 0.04

if (tp2.min<0.05) {tp2.min=0.05}

if (tp2.max>0.95) {tp2.max=0.95}

}

tp.pctlrange<-quantile(xTMP,probs=c(tp2.min,tp2.max),na.rm=TRUE)

tp.range<-unique(xTMP[xTMP>tp.pctlrange[1] & xTMP<tp.pctlrange[2]])

while (length(tp.range)>5) {

tmp.pct3<-quantile(tp.range,probs=c(0,0.25,0.5,0.75,1),type=3)

tmp.llk3<-rep(NA,3)

for (k in (2:4)) {

tmp.X<-(xTMP>tmp.pct3[k])*(xTMP-tmp.pct3[k]); wdtmp1<-cbind(wdtmp,tmp.X)

if (opt=="glm" | opt=="gee") tmp.mdl<-setglm(fml, i, wdtmp1);

if (opt=="gam") tmp.mdl<-setgam(fml, i, wdtmp1);

tmp.llk3[k-1]<-logLik(tmp.mdl)

rm(wdtmp1, tmp.X)

}

tmp.min3<-which.max(tmp.llk3)

tp.range<-tp.range[tp.range>=tmp.pct3[tmp.min3] & tp.range<=tmp.pct3[tmp.min3+2]]

}

if (length(tp.range)>0) {

if (length(tp.range)==1) {tp.val=tp.range[1];} else {

tmp.llk<-rep(NA,length(tp.range))

for (k in (1:length(tp.range))) {

tmp.X<-(xTMP>tp.range[k])*(xTMP-tp.range[k]); wdtmp1<-cbind(wdtmp,tmp.X)

if (opt=="glm" | opt=="gee") tmp.mdl<-setglm(fml, i, wdtmp1);

if (opt=="gam") tmp.mdl<-setgam(fml, i, wdtmp1);

tmp.llk[k]<-logLik(tmp.mdl)

rm(wdtmp1, tmp.X)

}

tp.val<-tp.range[which.max(tmp.llk)]

}

} else { tp.val<-tp.pctlrange[1];}

return(round(tp.val,dec));

}

get2lines<-function(i,j,g,tp.value,opt) {

if (is.na(g)) {fmladj1<-fmladj;wdtmp<-removeNA(i,j,WD);

} else if (g<nblv) {fmladj1<-fmladj;wdtmp0<-WD[WD[,bvar]==blv[g],];wdtmp<-removeNA(i,j,wdtmp0);

} else {fmladj1<-paste(fmladj,"+factor(",bvar,")",sep="");wdtmp<-removeNA(i,j,WD);}

xTMP<-wdtmp[,xvname[j]]

tmp.X1<-(xTMP<=tp.value)*(xTMP-tp.value)

tmp.X2<-(xTMP> tp.value)*(xTMP-tp.value)

wdtmp1<-cbind(wdtmp,xTMP,tmp.X1,tmp.X2)

fml0<-paste(yvname[i],"~xTMP+tmp.X2",fmladj1)

fml1<-paste(yvname[i],"~tmp.X1+tmp.X2",fmladj1)

fml2<-paste(yvname[i],"~xTMP",fmladj1)

fmlp<-paste(yvname[i],"~xTMP+tmp.X2")

tmpn<-nrow(wdtmp)

if (opt=="glm") {

tmp.mdl0<-setglm(fml0,i,wdtmp1); tmp.mdl1<-setglm(fml1,i,wdtmp1)

tmp.mdl2<-setglm(fml2,i,wdtmp1); tmp.mdlp<-setglm(fmlp,i,wdtmp1)

}

if (opt=="gam") {

tmp.mdl0<-setgam(fml0,i,wdtmp1); tmp.mdl1<-setgam(fml1,i,wdtmp1)

tmp.mdl2<-setgam(fml2,i,wdtmp1); tmp.mdlp<-setgam(fmlp,i,wdtmp1)

}

if (opt=="gee") {

tmp.mdl0<-setgee(fml0,i,wdtmp1); tmp.mdl1<-setgee(fml1,i,wdtmp1)

tmp.mdl2<-setgee(fml2,i,wdtmp1); tmp.mdlp<-setglm(fmlp,i,wdtmp1)

}

pd<-predict(tmp.mdlp,data.frame(xTMP=tp.value,tmp.X2=0), se.fit=TRUE)

prd<-paste(numfmt(pd$fit,dec)," (",numfmt(pd$fit-1.96*pd$se.fit,dec),", ", numfmt(pd$fit+1.96*pd$se.fit, dec),")",sep="")

m2<-mdl2oo(tmp.mdl2,"xTMP",opt)

m1<-mdl2oo(tmp.mdl1,c("tmp.X1","tmp.X2"),opt)

m0<-mdl2oo(tmp.mdl0,"tmp.X2",opt)

if (opt=="gee") {

plrt<-try(anova(tmp.mdl0,tmp.mdl2)$"P(>|Chi|)",TRUE)

plrt<-ifelse((plrt<="9" && plrt>="0"), pvformat(plrt,3),"-")

} else {plrt<- pvformat(1-pchisq(2*(logLik(tmp.mdl0)[1]-logLik(tmp.mdl2)[1]),1),3);}

oo<-list(c("",m2,"",tp.value,m1,m0,prd,plrt),tmpn)

return(oo)

}

get3lines<-function(i,j,g,tp.value,opt) {

if (is.na(g)) {fmladj1<-fmladj;wdtmp<-removeNA(i,j,WD);

} else if (g<nblv) {fmladj1<-fmladj;wdtmp0<-WD[WD[,bvar]==blv[g],];wdtmp<-removeNA(i,j,wdtmp0);

} else {fmladj1<-paste(fmladj,"+factor(",bvar,")",sep="");wdtmp<-removeNA(i,j,WD);}

xTMP<-wdtmp[,xvname[j]]; tp1<-tp.value[1]; tp2<-tp.value[2]

tmp.X1<- (xTMP< tp1)*(xTMP-tp1)

tmp.X2<-((xTMP>=tp1) & (xTMP<=tp2))*(xTMP-tp1)

tmp.X3<- (xTMP> tp2)*(xTMP-tp2)

tmp.B1<- (xTMP>tp2)

wdtmp1<-cbind(wdtmp,xTMP,tmp.X1,tmp.X2,tmp.X3,tmp.B1)

fml0<-paste(yvname[i],"~xTMP+tmp.X1+tmp.X3+tmp.B1",fmladj1)

fml1<-paste(yvname[i],"~tmp.X1+tmp.X2+tmp.X3+tmp.B1",fmladj1)

fml2<-paste(yvname[i],"~xTMP",fmladj1)

tmpn<-nrow(wdtmp)

if (opt=="glm") {

tmp.mdl0<-setglm(fml0,i,wdtmp1); tmp.mdl1<-setglm(fml1,i,wdtmp1); tmp.mdl2<-setglm(fml2,i,wdtmp1);

}

if (opt=="gam") {

tmp.mdl0<-setgam(fml0,i,wdtmp1); tmp.mdl1<-setgam(fml1,i,wdtmp1); tmp.mdl2<-setgam(fml2,i,wdtmp1);

}

if (opt=="gee") {

tmp.mdl0<-setgee(fml0,i,wdtmp1); tmp.mdl1<-setgee(fml1,i,wdtmp1); tmp.mdl2<-setgee(fml2,i,wdtmp1);

}

m2<-mdl2oo(tmp.mdl2,"xTMP",opt)

m1<-mdl2oo(tmp.mdl1,c("tmp.X1","tmp.X2","tmp.X3"),opt)

m0<-mdl2oo(tmp.mdl0,c("tmp.X1","tmp.X3"),opt)

if (opt=="gee") {

plrt<-try(anova(tmp.mdl0,tmp.mdl2)$"P(>|Chi|)",TRUE)

plrt<-ifelse((plrt<="9" && plrt>="0"), pvformat(plrt,3),"-")

} else {plrt<- pvformat(1-pchisq(2*(logLik(tmp.mdl0)[1]-logLik(tmp.mdl2)[1]),1),3);}

oo<-list(c("",m2,"",paste(tp.value,collapse=", "),m1,m0,plrt),tmpn)

return(oo)

}

getci4tp<-function(i,j,g,opt,tp0=NA) {

set.seed(123456)

if (is.na(g)) {wdt<-removeNA(i,j,WD);

} else if (g<nblv) {wdt<-WD[WD[,bvar]==blv[g],];wdt<-removeNA(i,j,wdt);

} else {wdt<-removeNA(i,j,WD);}

nnwd<-nrow(wdt); tp.vv<-rep(NA,1000)

if (!is.na(tp0)) {

tpp0 = sum(wdt[,xvname[j]] < tp0)/length(wdt[,xvname[j]])

tppmin = max(tpp0 - 0.09, 0.05)

tppmax = min(tpp0 + 0.09, 0.95)

} else {

tppmin = NA; tppmax = NA

}

for (s in (1:1000)) {

WDi<-wdt[sample(1:nnwd,nnwd,replace=T),]

tp.vv[s]<-get.tpval(i, j, NA, opt, WDi, tppmin, tppmax); rm(WDi)

}

tpci<-paste(quantile(tp.vv,probs=c(0.025,0.975)),collapse=", ")

return(tpci);

}

vlabelN<-(substr(vlabel,1,1)==" ");

vlabelZ<-vlabel[vlabelN];vlabelV<-vlabel[!vlabelN]

vnameV<-vname[!vlabelN];vnameZ<-vname[vlabelN];

allvname<-c(yvname,xvname,bvar,avname,subjvname,vname.start,vname.stop,timevname,"weights");

allvname<-allvname[!is.na(allvname)]

WD<-WD[,allvname];

w<-c("<html><head>","<meta http-equiv=\"Content-Type\" content=\"text/html\" charset=\"gb2312\" /></head><body>")

if (!is.na(avname[1])) {

if (sum((saf=="s" | saf=="S") & alv>0)>0) w<-c(w,"</br>Spline smoothing only applies for continuous variables")

if (!is.na(subjvname)) saf<-rep(0,length(saf))

}

if (sum(xlv>0)>0) w<-c(w,"Categorical exposure variables were ignored")

xvname<-xvname[xlv==0];

if (!is.na(subjvname)) WD<-WD[order(WD[,subjvname]),];

fmladj<-""; avb=""; smoothav<-0;

if (!is.na(avname[1])) {

avb<-vlabelV[match(avname,vnameV)];

avname_ <- avname

smoothavi<-((saf=="s" | saf=="S") & alv==0)

smoothav<-sum(smoothavi)

avname_[smoothavi]<-paste("s(",avname[smoothavi],")",sep="")

avb1<-avb

avb1[smoothavi]<-paste(avb[smoothavi],"(Smooth)",sep="")

avname_[alv>0]<-paste("factor(",avname[alv>0],")",sep="")

fmladj<-paste("+",paste(avname_,collapse="+"))

}

if (is.na(bvar)) {

blvb<-"N"; blvb_<-"N"; nblv<-1; blbl<-"";

} else {

blbl<-vlabelV[match(bvar,vnameV)]; if (is.na(blbl)) blbl<-bvar;

blv<-levels(factor(WD[,bvar])); nblv<-length(blv)+1

blvb_<-vlabelZ[match(paste(bvar,".",blv,sep=""),vnameZ)];

blvb_[is.na(blvb_)]<-blv[is.na(blvb_)];

blvb<-c(paste(blbl,blvb_,sep="="),"Total");

blvb_<-c(blvb_,"Total")

WD<-WD[!is.na(WD[,bvar]),]

}

ny=length(yvname); nx=length(xvname);

xb<-vlabelV[match(xvname,vnameV)]; xb[is.na(xb)]<-xvname[is.na(xb)]

yb<-vlabelV[match(yvname,vnameV)]; yb[is.na(yb)]<-yvname[is.na(yb)]

opt<-ifelse(!is.na(subjvname), "gee", ifelse(smoothav>0, "gam", "glm")) ;

colprn<-parm[3]

if (is.na(par1)) par1<-"";

if (is.numeric(par1)) {tp.vv<-par1;

} else {

tmp<-as.numeric(strsplit(par1," ")[[1]]); tp.vv<-c(tmp[!is.na(tmp)],NA)

}

prn<-ifelse(!is.na(bvar), "S", ifelse(nx>1 & ny==1, "X", "Y"));

if (length(tp.vv)>2) tp.vv<-tp.vv[1:2]

ntp<-length(tp.vv);

getci<-FALSE

prnopt<-c("β (95%CI) Pvalue / OR (95%CI) Pvalue", "β (95%CI) Pvalue", "β+se / OR (95%CI) *P<0.05 **P<0.01 ***P<0.001")

if (ntp==1) {

cc0<-c("模型 I","&nbsp&nbsp一条直线效应");

cc0<-c(cc0,"模型 II","&nbsp&nbsp折点(K)","&nbsp&nbsp &lt K 段效应 1","&nbsp&nbsp &gt K 段效应 2","&nbsp&nbsp 2与1的效应差")

cc0<-c(cc0,"&nbsp&nbsp折点处方程预测值")

if (is.na(tp.vv[1]) & !is.na(parm[1])) getci<-TRUE;

} else {

cc0<-c("模型 I","&nbsp&nbsp一条直线效应");

cc0<-c(cc0,"模型 II","&nbsp&nbsp折点(K1,K2)","&nbsp&nbsp &lt K1 段效应 1","&nbsp&nbsp K1-K2 段效应 2","&nbsp&nbsp &gt K2 段效应 3")

cc0<-c(cc0,"&nbsp&nbsp 1与2的效应差","&nbsp&nbsp 3与2的效应差")

}

if (opt=="gee") {cc0<-c(cc0,"ANOVA 两模型比较");} else {cc0<-c(cc0,"对数似然比检验");}

if (getci) cc0<-c(cc0,"折点的95可信区间");

sink(paste(ofname,".lst",sep=""))

w<-c(w,paste("<h2>", title, "</h2>"))

nn<-c("Outcome","Exposure",blvb);

if (prn=="Y") {

for (j in 1:nx) {

tt<-cc0;

for (i in 1:ny) {

if (is.na(tp.vv[1])) {tp.v<-get.tpval(i,j,NA,opt,NA);} else {tp.v<-tp.vv;}

if (ntp==1) tmpij<-get2lines(i,j,NA,tp.v,opt);

if (ntp==2) tmpij<-get3lines(i,j,NA,tp.v,opt);

if (getci) {tt<-cbind(tt,c(tmpij[[1]],getci4tp(i,j,NA,opt,tp.v)));} else {tt<-cbind(tt,tmpij[[1]]);}

nn<-rbind(nn,c(yb[i],xb[j],tmpij[[2]]))

}

tt<-rbind(c("Outcome: ",yb),tt)

w<-c(w,paste("</br>For exposure:",xb[j]))

w<-c(w,"</br><table border=3>", mat2htmltable(tt), "</table>")

}

}

if (prn=="X") {

for (i in 1:ny) {

tt<-cc0;

for (j in 1:nx) {

if (is.na(tp.vv[1])) {tp.v<-get.tpval(i,j,NA,opt,NA);} else {tp.v<-tp.vv;}

if (ntp==1) tmpij<-get2lines(i,j,NA,tp.v,opt);

if (ntp==2) tmpij<-get3lines(i,j,NA,tp.v,opt);

if (getci) {tt<-cbind(tt,c(tmpij[[1]],getci4tp(i,j,NA,opt,tp.v)));} else {tt<-cbind(tt,tmpij[[1]]);}

nn<-rbind(nn,c(yb[i],xb[j],tmpij[[2]]))

}

tt<-rbind(c("Exposure: ",xb),tt)

w<-c(w,paste("</br>For outcome:",yb[i]))

w<-c(w,"</br><table border=3>", mat2htmltable(tt), "</table>")

}

}

if (prn=="S") {

for (i in 1:ny) {

tt<-cc0;

for (j in 1:nx) {

nnij<-c(yb[i],xb[j])

for (g in 1:nblv) {

if (is.na(tp.vv[1])) {tp.v<-get.tpval(i,j,g,opt,NA);} else {tp.v<-tp.vv;}

if (ntp==1) tmpij<-get2lines(i,j,g,tp.v,opt);

if (ntp==2) tmpij<-get3lines(i,j,g,tp.v,opt);

if (getci) {tt<-cbind(tt,c(tmpij[[1]],getci4tp(i,j,g,opt,tp.v)));} else {tt<-cbind(tt,tmpij[[1]]);}

nnij<-c(nnij,tmpij[[2]]

}

nn<-rbind(nn,nnij)

}

tt<-rbind(c(blbl,blvb_),tt)

w<-c(w,paste("</br>For outcome:",yb[i]))

w<-c(w,paste("</br>For Exposure:",xb[j]))

w<-c(w,"</br><table border=3>", mat2htmltable(tt), "</table>")

}

}

**The relevant code for threshold effect analysis between DII and Diarrhea in EmpowerStats version 3.0 (http://www.empowerstats.net/analysis).**

Sys.setlocale("LC_TIME", "C")

library(doBy,lib.loc=R.LibLocation)

library(plotrix,lib.loc=R.LibLocation)

library(stringi,lib.loc=R.LibLocation)

library(stringr,lib.loc=R.LibLocation)

library(survival,lib.loc=R.LibLocation)

library(rms,lib.loc=R.LibLocation)

library(nnet,lib.loc=R.LibLocation)

library(car,lib.loc=R.LibLocation)

library(mgcv,lib.loc=R.LibLocation)

pdfwd<-6; pdfht<-6

setwd("C:/Users/79156/Desktop/nhances2005-2010/_1_tbl")

load("C:/Users/79156/Desktop/nhances2005-2010/FUXIE_sz.Rdata")

if (length(which(ls()=="EmpowerStatsR"))==0) EmpowerStatsR<-get(ls()[1])

names(EmpowerStatsR)<-toupper(names(EmpowerStatsR))

#--#

vname<-c("_N_","_STAT_","_TOTAL_","SEQN","WEIGHT","GENDER","GENDER.0","GENDER.1")

vlabel<-c("样本量(%)","统计量","合计","SEQN","WEIGHT","GENDER"," 0"," 1")

vname<-c(vname,"YEAR","RACE","RACE.1","RACE.2","RACE.3","RACE.4","RACE.5")

vlabel<-c(vlabel,"YEAR","RACE"," 1"," 2"," 3"," 4"," 5")

vname<-c(vname,"BOWEL","BOWEL.0","BOWEL.1","CANER","CANER.0","CANER.1")

vlabel<-c(vlabel,"BOWEL"," 0"," 1","CANER"," 0"," 1")

vname<-c(vname,"ALBUMIN","ALT","AST","ALKALINE_PHOSPHATASE")

vlabel<-c(vlabel,"ALBUMIN","ALT","AST","ALKALINE_PHOSPHATASE")

vname<-c(vname,"CHOLESTEROL","GST","TOTAL.PROTEIN","TRIGLYCERIDE")

vlabel<-c(vlabel,"CHOLESTEROL","GST","TOTAL.PROTEIN","TRIGLYCERIDE")

vname<-c(vname,"GLOBULIN","WBC","LYMPHOCYTE","MONOCYTE","NEUTROPHIL")

vlabel<-c(vlabel,"GLOBULIN","WBC","LYMPHOCYTE","MONOCYTE","NEUTROPHIL")

vname<-c(vname,"EOSINOPHIL","RBC","HB","PLT","CRP","INFLAMMATORY")

vlabel<-c(vlabel,"EOSINOPHIL","RBC","HB","PLT","CRP","INFLAMMATORY")

vname<-c(vname,"SOMKING","SOMKING.0","SOMKING.1","SOMKING.2")

vlabel<-c(vlabel,"SOMKING"," 0"," 1"," 2")

vname<-c(vname,"DIET","DII","BMI","BMI.1","BMI.2","BMI.3")

vlabel<-c(vlabel,"DIET","DII","BMI"," 1"," 2"," 3")

vname<-c(vname,"ALBUMIN_QUANTILE","ALBUMIN_QUANTILE.1","ALBUMIN_QUANTILE.2","ALBUMIN_QUANTILE.3","ALBUMIN_QUANTILE.4")

vlabel<-c(vlabel,"ALBUMIN_QUANTILE"," 1"," 2"," 3"," 4")

vname<-c(vname,"ALT_QUANTILE","ALT_QUANTILE.1","ALT_QUANTILE.2","ALT_QUANTILE.3","ALT_QUANTILE.4")

vlabel<-c(vlabel,"ALT_QUANTILE"," 1"," 2"," 3"," 4")

vname<-c(vname,"ALKALINE_PHOSPHATASE_QUANTILE","ALKALINE_PHOSPHATASE_QUANTILE.1","ALKALINE_PHOSPHATASE_QUANTILE.2","ALKALINE_PHOSPHATASE_QUANTILE.3","ALKALINE_PHOSPHATASE_QUANTILE.4")

vlabel<-c(vlabel,"ALKALINE_PHOSPHATASE_QUANTILE"," 1"," 2"," 3"," 4")

vname<-c(vname,"GLOBULIN_QUANTILE","GLOBULIN_QUANTILE.1","GLOBULIN_QUANTILE.2","GLOBULIN_QUANTILE.3","GLOBULIN_QUANTILE.4")

vlabel<-c(vlabel,"GLOBULIN_QUANTILE"," 1"," 2"," 3"," 4")

vname<-c(vname,"WBC_QUANTILE","WBC_QUANTILE.1","WBC_QUANTILE.2","WBC_QUANTILE.3","WBC_QUANTILE.4")

vlabel<-c(vlabel,"WBC_QUANTILE"," 1"," 2"," 3"," 4")

vname<-c(vname,"NEUTROPHIL_QUANTILE","NEUTROPHIL_QUANTILE.1","NEUTROPHIL_QUANTILE.2","NEUTROPHIL_QUANTILE.3","NEUTROPHIL_QUANTILE.4")

vlabel<-c(vlabel,"NEUTROPHIL_QUANTILE"," 1"," 2"," 3"," 4")

vname<-c(vname,"RBC_QUANTILE","RBC_QUANTILE.1","RBC_QUANTILE.2","RBC_QUANTILE.3","RBC_QUANTILE.4")

vlabel<-c(vlabel,"RBC_QUANTILE"," 1"," 2"," 3"," 4")

vname<-c(vname,"HB_QUANTILE","HB_QUANTILE.1","HB_QUANTILE.2","HB_QUANTILE.3","HB_QUANTILE.4")

vlabel<-c(vlabel,"HB_QUANTILE"," 1"," 2"," 3"," 4")

vname<-c(vname,"PLT_QUANTILE","PLT_QUANTILE.1","PLT_QUANTILE.2","PLT_QUANTILE.3","PLT_QUANTILE.4")

vlabel<-c(vlabel,"PLT_QUANTILE"," 1"," 2"," 3"," 4")

vname<-c(vname,"CRP_QUANTILE","CRP_QUANTILE.1","CRP_QUANTILE.2","CRP_QUANTILE.3","CRP_QUANTILE.4")

vlabel<-c(vlabel,"CRP_QUANTILE"," 1"," 2"," 3"," 4")

vname<-c(vname,"AAPR","AAPR.1","AAPR.2","AAPR.3","AAPR.4")

vlabel<-c(vlabel,"AAPR"," 1"," 2"," 3"," 4")

vname<-c(vname,"NLR","NLR.1","NLR.2","NLR.3","NLR.4","PNLR","PNLR.1","PNLR.2","PNLR.3","PNLR.4")

vlabel<-c(vlabel,"NLR"," 1"," 2"," 3"," 4","PNLR"," 1"," 2"," 3"," 4")

vname<-c(vname,"LMR","LMR.1","LMR.2","LMR.3","LMR.4","LCR","LCR.1","LCR.2","LCR.3","LCR.4")

vlabel<-c(vlabel,"LMR"," 1"," 2"," 3"," 4","LCR"," 1"," 2"," 3"," 4")

vname<-c(vname,"PLR","PLR.1","PLR.2","PLR.3","PLR.4","IBI","IBI.1","IBI.2","IBI.3","IBI.4")

vlabel<-c(vlabel,"PLR"," 1"," 2"," 3"," 4","IBI"," 1"," 2"," 3"," 4")

slt.vname<-c()

library(MASS,lib.loc=R.LibLocation)

library(gdata,lib.loc=R.LibLocation)

library(geepack,lib.loc=R.LibLocation)

library(mgcv,lib.loc=R.LibLocation)

ofname<-"_1_tbl";

WD<-EmpowerStatsR; wd.subset="";

svy.DSN.YN <- FALSE;

weights<-1;weights.var <- NA;

WD<-cbind(WD,weights); WD<-WD[!is.na(weights),];

title<-"阈值效应分析";

attach(WD)

subjvname<-NA;

yv<-cbind(BOWEL);

yvname<-c('BOWEL');

yvar<-c('BOWEL');

ydist<-c('binomial');

ylink<-c('logit');

ylv<-c(2);

xv<-cbind(DII);

xvname<-c('DII');

xvar<-c('DII');

xlv<-c(0);

sxf<-NA;

svname<-NA; sv<-NA; slv<-NA;

av<-cbind(GENDER,YEAR,RACE,CANER,ALT,AST,CHOLESTEROL,GST,TRIGLYCERIDE,EOSINOPHIL,SOMKING);

avname<-c('GENDER','YEAR','RACE','CANER','ALT','AST','CHOLESTEROL','GST','TRIGLYCERIDE','EOSINOPHIL','SOMKING');

if (!is.na(avname[1])) avlbl<-vlabel[match(avname, vname)];

nadj<-length(avname);alv<-c(2,0,5,2,0,0,0,0,0,0,3);

saf<-c(NA,0,0,0,0,0,0,0,0,0,0,0)[-1];

timev<-NA; timevname<-NA;

bv<-NA; bvar<-NA;

colv<-NA; colvname<-NA;

v.start<-NA; vname.start<-NA;

v.stop<-NA; vname.stop<-NA;

par1<-"自动寻找最佳拐点";dec<-3;parm<-c(NA, NA, 1,NA, 0);

if (!exists("pdfwd")) pdfwd<-6;

if (!exists("pdfht")) pdfht<-6;

##R package## MASS gdata geepack mgcv ##R package##;

pvformat<-function(p,dec) {

pp <- sprintf(paste("%.",dec,"f",sep=""),as.numeric(p))

if (is.matrix(p)) {pp<-matrix(pp, nrow=nrow(p)); colnames(pp)<-colnames(p);rownames(pp)<-rownames(p);}

lw <- paste("<",substr("0.00000000000",1,dec+1),"1",sep="");

pp[as.numeric(p)<(1/10^dec)]<-lw

return(pp)

}

numfmt<-function(p,dec) {

if (is.list(p)) p<-as.matrix(p)

pp <- sprintf(paste("%.",dec,"f",sep=""),as.numeric(p))

if (is.matrix(p)) {pp<-matrix(pp, nrow=nrow(p));colnames(pp)<-colnames(p);rownames(pp)<-rownames(p);}

pp[as.numeric(p)>10000000]<- "inf."

pp[is.na(p) | gsub(" ","",p)==""]<- ""

pp[p=="-Inf"]<-"-Inf"

pp[p=="Inf"]<-"Inf"

return(pp)

}

mat2htmltable<-function(mat) {

t1<- apply(mat,1,function(z) paste(z,collapse="</td><td>"))

t2<- paste("<tr><td>",t1,"</td></tr>")

return(paste(t2,collapse=" "))

}

setgam<-function(fml,yi,wdtmp) {

if (ydist[yi]=="") ydist[yi]<-"gaussian"

if (ydist[yi]=="exact") ydist[yi]<-"binomial"

if (ydist[yi]=="breslow") ydist[yi]<-"binomial"

if (ydist[yi]=="gaussian") mdl<-try(gam(formula(fml),weights=wdtmp$weights,data=wdtmp, family=gaussian(link="identity")))

if (ydist[yi]=="binomial") mdl<-try(gam(formula(fml),weights=wdtmp$weights,data=wdtmp, family=binomial(link="logit")))

if (ydist[yi]=="poisson") mdl<-try(gam(formula(fml),weights=wdtmp$weights,data=wdtmp, family=poisson(link="log")))

if (ydist[yi]=="gamma") mdl<-try(gam(formula(fml),weights=wdtmp$weights,data=wdtmp, family=Gamma(link="inverse")))

if (ydist[yi]=="negbin") mdl<-try(gam(formula(fml),weights=wdtmp$weights,data=wdtmp, family=negbin(c(1,10), link="log")))

return(mdl)

}

setgee<-function(fml,yi, wdtmp) {

if (ydist[yi]=="") ydist[yi]<-"gaussian"

if (ydist[yi]=="exact") ydist[yi]<-"binomial"

if (ydist[yi]=="breslow") ydist[yi]<-"binomial"

if (ydist[yi]=="gaussian") md<-try(geeglm(formula(fml),id=wdtmp[,subjvname],corstr=gee.TYPE,family="gaussian",weights=wdtmp$weights,data=wdtmp))

if (ydist[yi]=="binomial") md<-try(geeglm(formula(fml),id=wdtmp[,subjvname],corstr=gee.TYPE,family="binomial",weights=wdtmp$weights,data=wdtmp))

if (ydist[yi]=="poisson") md<-try(geeglm(formula(fml),id=wdtmp[,subjvname],corstr=gee.TYPE,family="poisson",weights=wdtmp$weights,data=wdtmp))

if (ydist[yi]=="gamma") md<-try(geeglm(formula(fml),id=wdtmp[,subjvname],corstr=gee.TYPE,family="Gamma",weights=wdtmp$weights,data=wdtmp))

if (ydist[yi]=="negbin") md<-try(geeglm.nb(formula(fml),id=wdtmp[,subjvname],corstr=gee.TYPE,weights=wdtmp$weights,data=wdtmp))

return(md)

}

setglm<-function(fml,yi, wdtmp) {

if (ydist[yi]=="") ydist[yi]<-"gaussian"

if (ydist[yi]=="exact") ydist[yi]<-"binomial"

if (ydist[yi]=="breslow") ydist[yi]<-"binomial"

if (ydist[yi]=="gaussian") md<-try(glm(formula(fml),family="gaussian",weights=wdtmp$weights,data=wdtmp))

if (ydist[yi]=="binomial") md<-try(glm(formula(fml),family="binomial",weights=wdtmp$weights,data=wdtmp))

if (ydist[yi]=="poisson") md<-try(glm(formula(fml),family="poisson",weights=wdtmp$weights,data=wdtmp))

if (ydist[yi]=="gamma") md<-try(glm(formula(fml),family="Gamma",weights=wdtmp$weights,data=wdtmp))

if (ydist[yi]=="negbin") md<-try(glm.nb(formula(fml),weights=wdtmp$weights,data=wdtmp))

return(md)

}

mdl2oo<-function(mdl, xxname, opt) {

if (is.na(mdl[[1]][1])) return(rep(" ",times=length(xxname)))

if (substr(mdl[[1]][1],1,5)=="Error") return(rep(" ",times=length(xxname)))

decp<-dec+2; if (decp>4) decp<-4

gs<-summary(mdl); print(mdl$formula); print(gs)

if (opt=="gam") {gsparm <- gs$p.table; } else {gsparm <- gs$coefficients;}

gsp<-gsparm[match(xxname,rownames(gsparm)),]

if (length(xxname)==1) {beta<-gsp[1]; se<-gsp[2]; pv<-gsp[4];

} else {beta<-gsp[,1]; se<-gsp[,2]; pv<-gsp[,4]; }

ci1<- beta-1.96*se; ci2<- beta+1.96*se

pvx<-substr(rep("****",length(pv)),1,(pv<=0.05)+(pv<=0.01)+(pv<=0.001))

if (colprn==3) {pvv<-pvx;} else {pvv<-pvformat(pv,decp);}

if ((colprn!=2) & (gs$family[[2]]=="log" | gs$family[[2]]=="logit")) {

o1<-paste(numfmt(exp(beta),dec)," (",numfmt(exp(ci1),dec),", ",numfmt(exp(ci2),dec),")",sep="")

} else {

if (colprn<3) {o1<-paste(numfmt(beta,dec), " (",numfmt(ci1,dec),", ",numfmt(ci2,dec),")",sep="")

} else {o1<-paste(numfmt(beta,dec), "+",numfmt(se,dec),sep="");}

}

o1<-paste(o1,pvv); o1[is.na(beta)]<-NA

return(o1)

}

removeNA<-function(i,j,wdf) {

vvv<-c(yvname[i],xvname[j],avname,subjvname,bvar,vname.start,vname.stop,timevname);

vvv<-vvv[!is.na(vvv)]; vvv<-vvv[vvv>" "]

tmp<-is.na(wdf[,vvv]);

return(wdf[apply(tmp,1,sum)==0,])

}

get.tpval<-function(i,j,g,opt,wdtmp, tppmin=NA, tppmax=NA) {

if (is.na(wdtmp)) {

if (is.na(g)) {wdtmp<-removeNA(i,j,WD);

} else if (g<nblv) {wdtmp0<-WD[WD[,bvar]==blv[g],]; wdtmp<-removeNA(i,j,wdtmp0);

} else {wdtmp<-removeNA(i,j,WD); }

}

if (is.na(g)) {fmladj1<-fmladj;

} else if (g<nblv) {fmladj1<-fmladj;

} else {fmladj1<-paste(fmladj,"+factor(",bvar,")",sep="");}

xTMP <- wdtmp[,xvname[j]]

tmp.ss<-seq(0.05,0.95,0.05)

tp<-quantile(xTMP,probs=tmp.ss,na.rm=TRUE)

tmp.llk<-rep(NA,length(tmp.ss))

fml<-paste(yvname[i],"~",xvname[j],"+tmp.X",fmladj1)

if (!is.na(tppmin) & !is.na(tppmax)) {

tp2.min = tppmin; tp2.max = tppmax;

} else {

for (k in (1:length(tmp.ss))) {

tmp.X<-(xTMP > tp[k])*(xTMP-tp[k]); wdtmp1<-cbind(wdtmp,tmp.X)

if (opt=="glm" | opt=="gee") tmp.mdl<-setglm(fml, i, wdtmp1);

if (opt=="gam") tmp.mdl<-setgam(fml, i, wdtmp1);

tmp.llk[k]<-logLik(tmp.mdl)

rm(wdtmp1, tmp.X)

}

tp1<-tmp.ss[which.max(tmp.llk)]

tp2.min = tp1 - 0.04

tp2.max = tp1 + 0.04

if (tp2.min<0.05) {tp2.min=0.05}

if (tp2.max>0.95) {tp2.max=0.95}

}

tp.pctlrange<-quantile(xTMP,probs=c(tp2.min,tp2.max),na.rm=TRUE)

tp.range<-unique(xTMP[xTMP>tp.pctlrange[1] & xTMP<tp.pctlrange[2]])

while (length(tp.range)>5) {

tmp.pct3<-quantile(tp.range,probs=c(0,0.25,0.5,0.75,1),type=3)

tmp.llk3<-rep(NA,3)

for (k in (2:4)) {

tmp.X<-(xTMP>tmp.pct3[k])*(xTMP-tmp.pct3[k]); wdtmp1<-cbind(wdtmp,tmp.X)

if (opt=="glm" | opt=="gee") tmp.mdl<-setglm(fml, i, wdtmp1);

if (opt=="gam") tmp.mdl<-setgam(fml, i, wdtmp1);

tmp.llk3[k-1]<-logLik(tmp.mdl)

rm(wdtmp1, tmp.X)

}

tmp.min3<-which.max(tmp.llk3)

tp.range<-tp.range[tp.range>=tmp.pct3[tmp.min3] & tp.range<=tmp.pct3[tmp.min3+2]]

}

if (length(tp.range)>0) {

if (length(tp.range)==1) {tp.val=tp.range[1];} else {

tmp.llk<-rep(NA,length(tp.range))

for (k in (1:length(tp.range))) {

tmp.X<-(xTMP>tp.range[k])*(xTMP-tp.range[k]); wdtmp1<-cbind(wdtmp,tmp.X)

if (opt=="glm" | opt=="gee") tmp.mdl<-setglm(fml, i, wdtmp1);

if (opt=="gam") tmp.mdl<-setgam(fml, i, wdtmp1);

tmp.llk[k]<-logLik(tmp.mdl)

rm(wdtmp1, tmp.X)

}

tp.val<-tp.range[which.max(tmp.llk)]

}

} else { tp.val<-tp.pctlrange[1];}

return(round(tp.val,dec));

}

get2lines<-function(i,j,g,tp.value,opt) {

if (is.na(g)) {fmladj1<-fmladj;wdtmp<-removeNA(i,j,WD);

} else if (g<nblv) {fmladj1<-fmladj;wdtmp0<-WD[WD[,bvar]==blv[g],];wdtmp<-removeNA(i,j,wdtmp0);

} else {fmladj1<-paste(fmladj,"+factor(",bvar,")",sep="");wdtmp<-removeNA(i,j,WD);}

xTMP<-wdtmp[,xvname[j]]

tmp.X1<-(xTMP<=tp.value)*(xTMP-tp.value)

tmp.X2<-(xTMP> tp.value)*(xTMP-tp.value)

wdtmp1<-cbind(wdtmp,xTMP,tmp.X1,tmp.X2)

fml0<-paste(yvname[i],"~xTMP+tmp.X2",fmladj1)

fml1<-paste(yvname[i],"~tmp.X1+tmp.X2",fmladj1)

fml2<-paste(yvname[i],"~xTMP",fmladj1)

fmlp<-paste(yvname[i],"~xTMP+tmp.X2")

tmpn<-nrow(wdtmp)

if (opt=="glm") {

tmp.mdl0<-setglm(fml0,i,wdtmp1); tmp.mdl1<-setglm(fml1,i,wdtmp1)

tmp.mdl2<-setglm(fml2,i,wdtmp1); tmp.mdlp<-setglm(fmlp,i,wdtmp1)

}

if (opt=="gam") {

tmp.mdl0<-setgam(fml0,i,wdtmp1); tmp.mdl1<-setgam(fml1,i,wdtmp1)

tmp.mdl2<-setgam(fml2,i,wdtmp1); tmp.mdlp<-setgam(fmlp,i,wdtmp1)

}

if (opt=="gee") {

tmp.mdl0<-setgee(fml0,i,wdtmp1); tmp.mdl1<-setgee(fml1,i,wdtmp1)

tmp.mdl2<-setgee(fml2,i,wdtmp1); tmp.mdlp<-setglm(fmlp,i,wdtmp1)

}

pd<-predict(tmp.mdlp,data.frame(xTMP=tp.value,tmp.X2=0), se.fit=TRUE)

prd<-paste(numfmt(pd$fit,dec)," (",numfmt(pd$fit-1.96*pd$se.fit,dec),", ", numfmt(pd$fit+1.96*pd$se.fit, dec),")",sep="")

m2<-mdl2oo(tmp.mdl2,"xTMP",opt)

m1<-mdl2oo(tmp.mdl1,c("tmp.X1","tmp.X2"),opt)

m0<-mdl2oo(tmp.mdl0,"tmp.X2",opt)

if (opt=="gee") {

plrt<-try(anova(tmp.mdl0,tmp.mdl2)$"P(>|Chi|)",TRUE)

plrt<-ifelse((plrt<="9" && plrt>="0"), pvformat(plrt,3),"-")

} else {plrt<- pvformat(1-pchisq(2*(logLik(tmp.mdl0)[1]-logLik(tmp.mdl2)[1]),1),3);}

oo<-list(c("",m2,"",tp.value,m1,m0,prd,plrt),tmpn)

return(oo)

}

get3lines<-function(i,j,g,tp.value,opt) {

if (is.na(g)) {fmladj1<-fmladj;wdtmp<-removeNA(i,j,WD);

} else if (g<nblv) {fmladj1<-fmladj;wdtmp0<-WD[WD[,bvar]==blv[g],];wdtmp<-removeNA(i,j,wdtmp0);

} else {fmladj1<-paste(fmladj,"+factor(",bvar,")",sep="");wdtmp<-removeNA(i,j,WD);}

xTMP<-wdtmp[,xvname[j]]; tp1<-tp.value[1]; tp2<-tp.value[2]

tmp.X1<- (xTMP< tp1)*(xTMP-tp1)

tmp.X2<-((xTMP>=tp1) & (xTMP<=tp2))*(xTMP-tp1)

tmp.X3<- (xTMP> tp2)*(xTMP-tp2)

tmp.B1<- (xTMP>tp2)

wdtmp1<-cbind(wdtmp,xTMP,tmp.X1,tmp.X2,tmp.X3,tmp.B1)

fml0<-paste(yvname[i],"~xTMP+tmp.X1+tmp.X3+tmp.B1",fmladj1)

fml1<-paste(yvname[i],"~tmp.X1+tmp.X2+tmp.X3+tmp.B1",fmladj1)

fml2<-paste(yvname[i],"~xTMP",fmladj1)

tmpn<-nrow(wdtmp)

if (opt=="glm") {

tmp.mdl0<-setglm(fml0,i,wdtmp1); tmp.mdl1<-setglm(fml1,i,wdtmp1); tmp.mdl2<-setglm(fml2,i,wdtmp1);

}

if (opt=="gam") {

tmp.mdl0<-setgam(fml0,i,wdtmp1); tmp.mdl1<-setgam(fml1,i,wdtmp1); tmp.mdl2<-setgam(fml2,i,wdtmp1);

}

if (opt=="gee") {

tmp.mdl0<-setgee(fml0,i,wdtmp1); tmp.mdl1<-setgee(fml1,i,wdtmp1); tmp.mdl2<-setgee(fml2,i,wdtmp1);

}

m2<-mdl2oo(tmp.mdl2,"xTMP",opt)

m1<-mdl2oo(tmp.mdl1,c("tmp.X1","tmp.X2","tmp.X3"),opt)

m0<-mdl2oo(tmp.mdl0,c("tmp.X1","tmp.X3"),opt)

if (opt=="gee") {

plrt<-try(anova(tmp.mdl0,tmp.mdl2)$"P(>|Chi|)",TRUE)

plrt<-ifelse((plrt<="9" && plrt>="0"), pvformat(plrt,3),"-")

} else {plrt<- pvformat(1-pchisq(2*(logLik(tmp.mdl0)[1]-logLik(tmp.mdl2)[1]),1),3);}

oo<-list(c("",m2,"",paste(tp.value,collapse=", "),m1,m0,plrt),tmpn)

return(oo)

}

getci4tp<-function(i,j,g,opt,tp0=NA) {

set.seed(123456)

if (is.na(g)) {wdt<-removeNA(i,j,WD);

} else if (g<nblv) {wdt<-WD[WD[,bvar]==blv[g],];wdt<-removeNA(i,j,wdt);

} else {wdt<-removeNA(i,j,WD);}

nnwd<-nrow(wdt); tp.vv<-rep(NA,1000)

if (!is.na(tp0)) {

tpp0 = sum(wdt[,xvname[j]] < tp0)/length(wdt[,xvname[j]])

tppmin = max(tpp0 - 0.09, 0.05)

tppmax = min(tpp0 + 0.09, 0.95)

} else {

tppmin = NA; tppmax = NA

}

for (s in (1:1000)) {

WDi<-wdt[sample(1:nnwd,nnwd,replace=T),]

tp.vv[s]<-get.tpval(i, j, NA, opt, WDi, tppmin, tppmax); rm(WDi)

}

tpci<-paste(quantile(tp.vv,probs=c(0.025,0.975)),collapse=", ")

return(tpci);

}

vlabelN<-(substr(vlabel,1,1)==" ");

vlabelZ<-vlabel[vlabelN];vlabelV<-vlabel[!vlabelN]

vnameV<-vname[!vlabelN];vnameZ<-vname[vlabelN];

allvname<-c(yvname,xvname,bvar,avname,subjvname,vname.start,vname.stop,timevname,"weights");

allvname<-allvname[!is.na(allvname)]

WD<-WD[,allvname];

w<-c("<html><head>","<meta http-equiv=\"Content-Type\" content=\"text/html\" charset=\"gb2312\" /></head><body>")

if (!is.na(avname[1])) {

if (sum((saf=="s" | saf=="S") & alv>0)>0) w<-c(w,"</br>Spline smoothing only applies for continuous variables")

if (!is.na(subjvname)) saf<-rep(0,length(saf))

}

if (sum(xlv>0)>0) w<-c(w,"Categorical exposure variables were ignored")

xvname<-xvname[xlv==0];

if (!is.na(subjvname)) WD<-WD[order(WD[,subjvname]),];

fmladj<-""; avb=""; smoothav<-0;

if (!is.na(avname[1])) {

avb<-vlabelV[match(avname,vnameV)];

avname_ <- avname

smoothavi<-((saf=="s" | saf=="S") & alv==0)

smoothav<-sum(smoothavi)

avname_[smoothavi]<-paste("s(",avname[smoothavi],")",sep="")

avb1<-avb

avb1[smoothavi]<-paste(avb[smoothavi],"(Smooth)",sep="")

avname_[alv>0]<-paste("factor(",avname[alv>0],")",sep="")

fmladj<-paste("+",paste(avname_,collapse="+"))

}

if (is.na(bvar)) {

blvb<-"N"; blvb_<-"N"; nblv<-1; blbl<-"";

} else {

blbl<-vlabelV[match(bvar,vnameV)]; if (is.na(blbl)) blbl<-bvar;

blv<-levels(factor(WD[,bvar])); nblv<-length(blv)+1

blvb_<-vlabelZ[match(paste(bvar,".",blv,sep=""),vnameZ)];

blvb_[is.na(blvb_)]<-blv[is.na(blvb_)];

blvb<-c(paste(blbl,blvb_,sep="="),"Total");

blvb_<-c(blvb_,"Total")

WD<-WD[!is.na(WD[,bvar]),]

}

ny=length(yvname); nx=length(xvname);

xb<-vlabelV[match(xvname,vnameV)]; xb[is.na(xb)]<-xvname[is.na(xb)]

yb<-vlabelV[match(yvname,vnameV)]; yb[is.na(yb)]<-yvname[is.na(yb)]

opt<-ifelse(!is.na(subjvname), "gee", ifelse(smoothav>0, "gam", "glm")) ;

colprn<-parm[3]

if (is.na(par1)) par1<-"";

if (is.numeric(par1)) {tp.vv<-par1;

} else {

tmp<-as.numeric(strsplit(par1," ")[[1]]); tp.vv<-c(tmp[!is.na(tmp)],NA)

}

prn<-ifelse(!is.na(bvar), "S", ifelse(nx>1 & ny==1, "X", "Y"));

if (length(tp.vv)>2) tp.vv<-tp.vv[1:2]

ntp<-length(tp.vv);

getci<-FALSE

prnopt<-c("β (95%CI) Pvalue / OR (95%CI) Pvalue", "β (95%CI) Pvalue", "β+se / OR (95%CI) *P<0.05 **P<0.01 ***P<0.001")

if (ntp==1) {

cc0<-c("模型 I","&nbsp&nbsp一条直线效应");

cc0<-c(cc0,"模型 II","&nbsp&nbsp折点(K)","&nbsp&nbsp &lt K 段效应 1","&nbsp&nbsp &gt K 段效应 2","&nbsp&nbsp 2与1的效应差")

cc0<-c(cc0,"&nbsp&nbsp折点处方程预测值")

if (is.na(tp.vv[1]) & !is.na(parm[1])) getci<-TRUE;

} else {

cc0<-c("模型 I","&nbsp&nbsp一条直线效应");

cc0<-c(cc0,"模型 II","&nbsp&nbsp折点(K1,K2)","&nbsp&nbsp &lt K1 段效应 1","&nbsp&nbsp K1-K2 段效应 2","&nbsp&nbsp &gt K2 段效应 3")

cc0<-c(cc0,"&nbsp&nbsp 1与2的效应差","&nbsp&nbsp 3与2的效应差")

}

if (opt=="gee") {cc0<-c(cc0,"ANOVA 两模型比较");} else {cc0<-c(cc0,"对数似然比检验");}

if (getci) cc0<-c(cc0,"折点的95可信区间");

sink(paste(ofname,".lst",sep=""))

w<-c(w,paste("<h2>", title, "</h2>"))

nn<-c("Outcome","Exposure",blvb);

if (prn=="Y") {

for (j in 1:nx) {

tt<-cc0;

for (i in 1:ny) {

if (is.na(tp.vv[1])) {tp.v<-get.tpval(i,j,NA,opt,NA);} else {tp.v<-tp.vv;}

if (ntp==1) tmpij<-get2lines(i,j,NA,tp.v,opt);

if (ntp==2) tmpij<-get3lines(i,j,NA,tp.v,opt);

if (getci) {tt<-cbind(tt,c(tmpij[[1]],getci4tp(i,j,NA,opt,tp.v)));} else {tt<-cbind(tt,tmpij[[1]]);}

nn<-rbind(nn,c(yb[i],xb[j],tmpij[[2]]))

}

tt<-rbind(c("Outcome: ",yb),tt)

w<-c(w,paste("</br>For exposure:",xb[j]))

w<-c(w,"</br><table border=3>", mat2htmltable(tt), "</table>")

}

}

if (prn=="X") {

for (i in 1:ny) {

tt<-cc0;

for (j in 1:nx) {

if (is.na(tp.vv[1])) {tp.v<-get.tpval(i,j,NA,opt,NA);} else {tp.v<-tp.vv;}

if (ntp==1) tmpij<-get2lines(i,j,NA,tp.v,opt);

if (ntp==2) tmpij<-get3lines(i,j,NA,tp.v,opt);

if (getci) {tt<-cbind(tt,c(tmpij[[1]],getci4tp(i,j,NA,opt,tp.v)));} else {tt<-cbind(tt,tmpij[[1]]);}

nn<-rbind(nn,c(yb[i],xb[j],tmpij[[2]]))

}

tt<-rbind(c("Exposure: ",xb),tt)

w<-c(w,paste("</br>For outcome:",yb[i]))

w<-c(w,"</br><table border=3>", mat2htmltable(tt), "</table>")

}

}

if (prn=="S") {

for (i in 1:ny) {

tt<-cc0;

for (j in 1:nx) {

nnij<-c(yb[i],xb[j])

for (g in 1:nblv) {

if (is.na(tp.vv[1])) {tp.v<-get.tpval(i,j,g,opt,NA);} else {tp.v<-tp.vv;}

if (ntp==1) tmpij<-get2lines(i,j,g,tp.v,opt);

if (ntp==2) tmpij<-get3lines(i,j,g,tp.v,opt);

if (getci) {tt<-cbind(tt,c(tmpij[[1]],getci4tp(i,j,g,opt,tp.v)));} else {tt<-cbind(tt,tmpij[[1]]);}

nnij<-c(nnij,tmpij[[2]])

}

nn<-rbind(nn,nnij

}

tt<-rbind(c(blbl,blvb_),tt)

w<-c(w,paste("</br>For outcome:",yb[i]))

w<-c(w,paste("</br>For Exposure:",xb[j]))

w<-c(w,"</br><table border=3>", mat2htmltable(tt), "</table>")

}

}

**The relevant code for threshold effect analysis between DII and Diarrhea in AAPR subgroups using EmpowerStats version 3.0 (http://www.empowerstats.net/analysis).**

Sys.setlocale("LC_TIME", "C")

library(doBy,lib.loc=R.LibLocation)

library(plotrix,lib.loc=R.LibLocation)

library(stringi,lib.loc=R.LibLocation)

library(stringr,lib.loc=R.LibLocation)

library(survival,lib.loc=R.LibLocation)

library(rms,lib.loc=R.LibLocation)

library(nnet,lib.loc=R.LibLocation)

library(car,lib.loc=R.LibLocation)

library(mgcv,lib.loc=R.LibLocation)

pdfwd<-6; pdfht<-6

setwd("C:/Users/79156/Desktop/nhances2005-2010/_2_tbl")

load("C:/Users/79156/Desktop/nhances2005-2010/FUXIE_sz.Rdata")

if (length(which(ls()=="EmpowerStatsR"))==0) EmpowerStatsR<-get(ls()[1])

names(EmpowerStatsR)<-toupper(names(EmpowerStatsR))

#--#

vname<-c("_N_","_STAT_","_TOTAL_","SEQN","WEIGHT","GENDER","GENDER.0","GENDER.1")

vlabel<-c("样本量(%)","统计量","合计","SEQN","WEIGHT","GENDER"," 0"," 1")

vname<-c(vname,"YEAR","RACE","RACE.1","RACE.2","RACE.3","RACE.4","RACE.5")

vlabel<-c(vlabel,"YEAR","RACE"," 1"," 2"," 3"," 4"," 5")

vname<-c(vname,"BOWEL","BOWEL.0","BOWEL.1","CANER","CANER.0","CANER.1")

vlabel<-c(vlabel,"BOWEL"," 0"," 1","CANER"," 0"," 1")

vname<-c(vname,"ALBUMIN","ALT","AST","ALKALINE_PHOSPHATASE")

vlabel<-c(vlabel,"ALBUMIN","ALT","AST","ALKALINE_PHOSPHATASE")

vname<-c(vname,"CHOLESTEROL","GST","TOTAL.PROTEIN","TRIGLYCERIDE")

vlabel<-c(vlabel,"CHOLESTEROL","GST","TOTAL.PROTEIN","TRIGLYCERIDE")

vname<-c(vname,"GLOBULIN","WBC","LYMPHOCYTE","MONOCYTE","NEUTROPHIL")

vlabel<-c(vlabel,"GLOBULIN","WBC","LYMPHOCYTE","MONOCYTE","NEUTROPHIL")

vname<-c(vname,"EOSINOPHIL","RBC","HB","PLT","CRP","INFLAMMATORY")

vlabel<-c(vlabel,"EOSINOPHIL","RBC","HB","PLT","CRP","INFLAMMATORY")

vname<-c(vname,"SOMKING","SOMKING.0","SOMKING.1","SOMKING.2")

vlabel<-c(vlabel,"SOMKING"," 0"," 1"," 2")

vname<-c(vname,"DIET","DII","BMI","BMI.1","BMI.2","BMI.3")

vlabel<-c(vlabel,"DIET","DII","BMI"," 1"," 2"," 3")

vname<-c(vname,"ALBUMIN_QUANTILE","ALBUMIN_QUANTILE.1","ALBUMIN_QUANTILE.2","ALBUMIN_QUANTILE.3","ALBUMIN_QUANTILE.4")

vlabel<-c(vlabel,"ALBUMIN_QUANTILE"," 1"," 2"," 3"," 4")

vname<-c(vname,"ALT_QUANTILE","ALT_QUANTILE.1","ALT_QUANTILE.2","ALT_QUANTILE.3","ALT_QUANTILE.4")

vlabel<-c(vlabel,"ALT_QUANTILE"," 1"," 2"," 3"," 4")

vname<-c(vname,"ALKALINE_PHOSPHATASE_QUANTILE","ALKALINE_PHOSPHATASE_QUANTILE.1","ALKALINE_PHOSPHATASE_QUANTILE.2","ALKALINE_PHOSPHATASE_QUANTILE.3","ALKALINE_PHOSPHATASE_QUANTILE.4")

vlabel<-c(vlabel,"ALKALINE_PHOSPHATASE_QUANTILE"," 1"," 2"," 3"," 4")

vname<-c(vname,"GLOBULIN_QUANTILE","GLOBULIN_QUANTILE.1","GLOBULIN_QUANTILE.2","GLOBULIN_QUANTILE.3","GLOBULIN_QUANTILE.4")

vlabel<-c(vlabel,"GLOBULIN_QUANTILE"," 1"," 2"," 3"," 4")

vname<-c(vname,"WBC_QUANTILE","WBC_QUANTILE.1","WBC_QUANTILE.2","WBC_QUANTILE.3","WBC_QUANTILE.4")

vlabel<-c(vlabel,"WBC_QUANTILE"," 1"," 2"," 3"," 4")

vname<-c(vname,"NEUTROPHIL_QUANTILE","NEUTROPHIL_QUANTILE.1","NEUTROPHIL_QUANTILE.2","NEUTROPHIL_QUANTILE.3","NEUTROPHIL_QUANTILE.4")

vlabel<-c(vlabel,"NEUTROPHIL_QUANTILE"," 1"," 2"," 3"," 4")

vname<-c(vname,"RBC_QUANTILE","RBC_QUANTILE.1","RBC_QUANTILE.2","RBC_QUANTILE.3","RBC_QUANTILE.4")

vlabel<-c(vlabel,"RBC_QUANTILE"," 1"," 2"," 3"," 4")

vname<-c(vname,"HB_QUANTILE","HB_QUANTILE.1","HB_QUANTILE.2","HB_QUANTILE.3","HB_QUANTILE.4")

vlabel<-c(vlabel,"HB_QUANTILE"," 1"," 2"," 3"," 4")

vname<-c(vname,"PLT_QUANTILE","PLT_QUANTILE.1","PLT_QUANTILE.2","PLT_QUANTILE.3","PLT_QUANTILE.4")

vlabel<-c(vlabel,"PLT_QUANTILE"," 1"," 2"," 3"," 4")

vname<-c(vname,"CRP_QUANTILE","CRP_QUANTILE.1","CRP_QUANTILE.2","CRP_QUANTILE.3","CRP_QUANTILE.4")

vlabel<-c(vlabel,"CRP_QUANTILE"," 1"," 2"," 3"," 4")

vname<-c(vname,"AAPR","AAPR.1","AAPR.2","AAPR.3","AAPR.4")

vlabel<-c(vlabel,"AAPR"," 1"," 2"," 3"," 4")

vname<-c(vname,"NLR","NLR.1","NLR.2","NLR.3","NLR.4","PNLR","PNLR.1","PNLR.2","PNLR.3","PNLR.4")

vlabel<-c(vlabel,"NLR"," 1"," 2"," 3"," 4","PNLR"," 1"," 2"," 3"," 4")

vname<-c(vname,"LMR","LMR.1","LMR.2","LMR.3","LMR.4","LCR","LCR.1","LCR.2","LCR.3","LCR.4")

vlabel<-c(vlabel,"LMR"," 1"," 2"," 3"," 4","LCR"," 1"," 2"," 3"," 4")

vname<-c(vname,"PLR","PLR.1","PLR.2","PLR.3","PLR.4","IBI","IBI.1","IBI.2","IBI.3","IBI.4")

vlabel<-c(vlabel,"PLR"," 1"," 2"," 3"," 4","IBI"," 1"," 2"," 3"," 4")

slt.vname<-c()

library(MASS,lib.loc=R.LibLocation)

library(gdata,lib.loc=R.LibLocation)

library(geepack,lib.loc=R.LibLocation)

library(mgcv,lib.loc=R.LibLocation)

ofname<-"_2_tbl";

WD<-EmpowerStatsR; wd.subset="";

svy.DSN.YN <- FALSE;

weights<-1;weights.var <- NA;

WD<-cbind(WD,weights); WD<-WD[!is.na(weights),];

title<-"阈值效应分析";

attach(WD)

subjvname<-NA;

yv<-cbind(BOWEL);

yvname<-c('BOWEL');

yvar<-c('BOWEL');

ydist<-c('binomial');

ylink<-c('logit');

ylv<-c(2);

xv<-cbind(DII);

xvname<-c('DII');

xvar<-c('DII');

xlv<-c(0);

sxf<-NA;

svname<-NA; sv<-NA; slv<-NA;

av<-cbind(GENDER,YEAR,RACE,CANER,ALT,AST,CHOLESTEROL,GST,TRIGLYCERIDE,EOSINOPHIL,SOMKING);

avname<-c('GENDER','YEAR','RACE','CANER','ALT','AST','CHOLESTEROL','GST','TRIGLYCERIDE','EOSINOPHIL','SOMKING');

if (!is.na(avname[1])) avlbl<-vlabel[match(avname, vname)];

nadj<-length(avname);alv<-c(2,0,5,2,0,0,0,0,0,0,3);

saf<-c(NA,0,0,0,0,0,0,0,0,0,0,0)[-1];

timev<-NA; timevname<-NA;

bv<-AAPR;bvar<-"AAPR";bvname<-"AAPR";

colv<-NA; colvname<-NA;

v.start<-NA; vname.start<-NA;

v.stop<-NA; vname.stop<-NA;

par1<-"自动寻找最佳拐点";dec<-3;parm<-c(NA, NA, 1,NA, 0);

if (!exists("pdfwd")) pdfwd<-6;

if (!exists("pdfht")) pdfht<-6;

##R package## MASS gdata geepack mgcv ##R package##;

pvformat<-function(p,dec) {

pp <- sprintf(paste("%.",dec,"f",sep=""),as.numeric(p))

if (is.matrix(p)) {pp<-matrix(pp, nrow=nrow(p)); colnames(pp)<-colnames(p);rownames(pp)<-rownames(p);}

lw <- paste("<",substr("0.00000000000",1,dec+1),"1",sep="");

pp[as.numeric(p)<(1/10^dec)]<-lw

return(pp)

}

numfmt<-function(p,dec) {

if (is.list(p)) p<-as.matrix(p)

pp <- sprintf(paste("%.",dec,"f",sep=""),as.numeric(p))

if (is.matrix(p)) {pp<-matrix(pp, nrow=nrow(p));colnames(pp)<-colnames(p);rownames(pp)<-rownames(p);}

pp[as.numeric(p)>10000000]<- "inf."

pp[is.na(p) | gsub(" ","",p)==""]<- ""

pp[p=="-Inf"]<-"-Inf"

pp[p=="Inf"]<-"Inf"

return(pp)

}

mat2htmltable<-function(mat) {

t1<- apply(mat,1,function(z) paste(z,collapse="</td><td>"))

t2<- paste("<tr><td>",t1,"</td></tr>")

return(paste(t2,collapse=" "))

}

setgam<-function(fml,yi,wdtmp) {

if (ydist[yi]=="") ydist[yi]<-"gaussian"

if (ydist[yi]=="exact") ydist[yi]<-"binomial"

if (ydist[yi]=="breslow") ydist[yi]<-"binomial"

if (ydist[yi]=="gaussian") mdl<-try(gam(formula(fml),weights=wdtmp$weights,data=wdtmp, family=gaussian(link="identity")))

if (ydist[yi]=="binomial") mdl<-try(gam(formula(fml),weights=wdtmp$weights,data=wdtmp, family=binomial(link="logit")))

if (ydist[yi]=="poisson") mdl<-try(gam(formula(fml),weights=wdtmp$weights,data=wdtmp, family=poisson(link="log")))

if (ydist[yi]=="gamma") mdl<-try(gam(formula(fml),weights=wdtmp$weights,data=wdtmp, family=Gamma(link="inverse")))

if (ydist[yi]=="negbin") mdl<-try(gam(formula(fml),weights=wdtmp$weights,data=wdtmp, family=negbin(c(1,10), link="log")))

return(mdl)

}

setgee<-function(fml,yi, wdtmp) {

if (ydist[yi]=="") ydist[yi]<-"gaussian"

if (ydist[yi]=="exact") ydist[yi]<-"binomial"

if (ydist[yi]=="breslow") ydist[yi]<-"binomial"

if (ydist[yi]=="gaussian") md<-try(geeglm(formula(fml),id=wdtmp[,subjvname],corstr=gee.TYPE,family="gaussian",weights=wdtmp$weights,data=wdtmp))

if (ydist[yi]=="binomial") md<-try(geeglm(formula(fml),id=wdtmp[,subjvname],corstr=gee.TYPE,family="binomial",weights=wdtmp$weights,data=wdtmp))

if (ydist[yi]=="poisson") md<-try(geeglm(formula(fml),id=wdtmp[,subjvname],corstr=gee.TYPE,family="poisson",weights=wdtmp$weights,data=wdtmp))

if (ydist[yi]=="gamma") md<-try(geeglm(formula(fml),id=wdtmp[,subjvname],corstr=gee.TYPE,family="Gamma",weights=wdtmp$weights,data=wdtmp))

if (ydist[yi]=="negbin") md<-try(geeglm.nb(formula(fml),id=wdtmp[,subjvname],corstr=gee.TYPE,weights=wdtmp$weights,data=wdtmp))

return(md)

}

setglm<-function(fml,yi, wdtmp) {

if (ydist[yi]=="") ydist[yi]<-"gaussian"

if (ydist[yi]=="exact") ydist[yi]<-"binomial"

if (ydist[yi]=="breslow") ydist[yi]<-"binomial"

if (ydist[yi]=="gaussian") md<-try(glm(formula(fml),family="gaussian",weights=wdtmp$weights,data=wdtmp))

if (ydist[yi]=="binomial") md<-try(glm(formula(fml),family="binomial",weights=wdtmp$weights,data=wdtmp))

if (ydist[yi]=="poisson") md<-try(glm(formula(fml),family="poisson",weights=wdtmp$weights,data=wdtmp))

if (ydist[yi]=="gamma") md<-try(glm(formula(fml),family="Gamma",weights=wdtmp$weights,data=wdtmp))

if (ydist[yi]=="negbin") md<-try(glm.nb(formula(fml),weights=wdtmp$weights,data=wdtmp))

return(md)

}

mdl2oo<-function(mdl, xxname, opt) {

if (is.na(mdl[[1]][1])) return(rep(" ",times=length(xxname)))

if (substr(mdl[[1]][1],1,5)=="Error") return(rep(" ",times=length(xxname)))

decp<-dec+2; if (decp>4) decp<-4

gs<-summary(mdl); print(mdl$formula); print(gs)

if (opt=="gam") {gsparm <- gs$p.table; } else {gsparm <- gs$coefficients;}

gsp<-gsparm[match(xxname,rownames(gsparm)),]

if (length(xxname)==1) {beta<-gsp[1]; se<-gsp[2]; pv<-gsp[4];

} else {beta<-gsp[,1]; se<-gsp[,2]; pv<-gsp[,4]; }

ci1<- beta-1.96*se; ci2<- beta+1.96*se

pvx<-substr(rep("****",length(pv)),1,(pv<=0.05)+(pv<=0.01)+(pv<=0.001))

if (colprn==3) {pvv<-pvx;} else {pvv<-pvformat(pv,decp);}

if ((colprn!=2) & (gs$family[[2]]=="log" | gs$family[[2]]=="logit")) {

o1<-paste(numfmt(exp(beta),dec)," (",numfmt(exp(ci1),dec),", ",numfmt(exp(ci2),dec),")",sep="")

} else {

if (colprn<3) {o1<-paste(numfmt(beta,dec), " (",numfmt(ci1,dec),", ",numfmt(ci2,dec),")",sep="")

} else {o1<-paste(numfmt(beta,dec), "+",numfmt(se,dec),sep="");}

}

o1<-paste(o1,pvv); o1[is.na(beta)]<-NA

return(o1)

}

removeNA<-function(i,j,wdf) {

vvv<-c(yvname[i],xvname[j],avname,subjvname,bvar,vname.start,vname.stop,timevname);

vvv<-vvv[!is.na(vvv)]; vvv<-vvv[vvv>" "]

tmp<-is.na(wdf[,vvv]);

return(wdf[apply(tmp,1,sum)==0,])

}

get.tpval<-function(i,j,g,opt,wdtmp, tppmin=NA, tppmax=NA) {

if (is.na(wdtmp)) {

if (is.na(g)) {wdtmp<-removeNA(i,j,WD);

} else if (g<nblv) {wdtmp0<-WD[WD[,bvar]==blv[g],]; wdtmp<-removeNA(i,j,wdtmp0);

} else {wdtmp<-removeNA(i,j,WD); }

}

if (is.na(g)) {fmladj1<-fmladj;

} else if (g<nblv) {fmladj1<-fmladj;

} else {fmladj1<-paste(fmladj,"+factor(",bvar,")",sep="");}

xTMP <- wdtmp[,xvname[j]]

tmp.ss<-seq(0.05,0.95,0.05)

tp<-quantile(xTMP,probs=tmp.ss,na.rm=TRUE)

tmp.llk<-rep(NA,length(tmp.ss))

fml<-paste(yvname[i],"~",xvname[j],"+tmp.X",fmladj1)

if (!is.na(tppmin) & !is.na(tppmax)) {

tp2.min = tppmin; tp2.max = tppmax;

} else {

for (k in (1:length(tmp.ss))) {

tmp.X<-(xTMP > tp[k])*(xTMP-tp[k]); wdtmp1<-cbind(wdtmp,tmp.X)

if (opt=="glm" | opt=="gee") tmp.mdl<-setglm(fml, i, wdtmp1);

if (opt=="gam") tmp.mdl<-setgam(fml, i, wdtmp1);

tmp.llk[k]<-logLik(tmp.mdl)

rm(wdtmp1, tmp.X)

}

tp1<-tmp.ss[which.max(tmp.llk)]

tp2.min = tp1 - 0.04

tp2.max = tp1 + 0.04

if (tp2.min<0.05) {tp2.min=0.05}

if (tp2.max>0.95) {tp2.max=0.95}

}

tp.pctlrange<-quantile(xTMP,probs=c(tp2.min,tp2.max),na.rm=TRUE)

tp.range<-unique(xTMP[xTMP>tp.pctlrange[1] & xTMP<tp.pctlrange[2]])

while (length(tp.range)>5) {

tmp.pct3<-quantile(tp.range,probs=c(0,0.25,0.5,0.75,1),type=3)

tmp.llk3<-rep(NA,3)

for (k in (2:4)) {

tmp.X<-(xTMP>tmp.pct3[k])*(xTMP-tmp.pct3[k]); wdtmp1<-cbind(wdtmp,tmp.X)

if (opt=="glm" | opt=="gee") tmp.mdl<-setglm(fml, i, wdtmp1);

if (opt=="gam") tmp.mdl<-setgam(fml, i, wdtmp1);

tmp.llk3[k-1]<-logLik(tmp.mdl)

rm(wdtmp1, tmp.X)

}

tmp.min3<-which.max(tmp.llk3)

tp.range<-tp.range[tp.range>=tmp.pct3[tmp.min3] & tp.range<=tmp.pct3[tmp.min3+2]]

}

if (length(tp.range)>0) {

if (length(tp.range)==1) {tp.val=tp.range[1];} else {

tmp.llk<-rep(NA,length(tp.range))

for (k in (1:length(tp.range))) {

tmp.X<-(xTMP>tp.range[k])*(xTMP-tp.range[k]); wdtmp1<-cbind(wdtmp,tmp.X)

if (opt=="glm" | opt=="gee") tmp.mdl<-setglm(fml, i, wdtmp1);

if (opt=="gam") tmp.mdl<-setgam(fml, i, wdtmp1);

tmp.llk[k]<-logLik(tmp.mdl)

rm(wdtmp1, tmp.X)

}

tp.val<-tp.range[which.max(tmp.llk)]

}

} else { tp.val<-tp.pctlrange[1];}

return(round(tp.val,dec));

}

get2lines<-function(i,j,g,tp.value,opt) {

if (is.na(g)) {fmladj1<-fmladj;wdtmp<-removeNA(i,j,WD);

} else if (g<nblv) {fmladj1<-fmladj;wdtmp0<-WD[WD[,bvar]==blv[g],];wdtmp<-removeNA(i,j,wdtmp0);

} else {fmladj1<-paste(fmladj,"+factor(",bvar,")",sep="");wdtmp<-removeNA(i,j,WD);}

xTMP<-wdtmp[,xvname[j]]

tmp.X1<-(xTMP<=tp.value)*(xTMP-tp.value)

tmp.X2<-(xTMP> tp.value)*(xTMP-tp.value)

wdtmp1<-cbind(wdtmp,xTMP,tmp.X1,tmp.X2)

fml0<-paste(yvname[i],"~xTMP+tmp.X2",fmladj1)

fml1<-paste(yvname[i],"~tmp.X1+tmp.X2",fmladj1)

fml2<-paste(yvname[i],"~xTMP",fmladj1)

fmlp<-paste(yvname[i],"~xTMP+tmp.X2")

tmpn<-nrow(wdtmp)

if (opt=="glm") {

tmp.mdl0<-setglm(fml0,i,wdtmp1); tmp.mdl1<-setglm(fml1,i,wdtmp1)

tmp.mdl2<-setglm(fml2,i,wdtmp1); tmp.mdlp<-setglm(fmlp,i,wdtmp1)

}

if (opt=="gam") {

tmp.mdl0<-setgam(fml0,i,wdtmp1); tmp.mdl1<-setgam(fml1,i,wdtmp1)

tmp.mdl2<-setgam(fml2,i,wdtmp1); tmp.mdlp<-setgam(fmlp,i,wdtmp1)

}

if (opt=="gee") {

tmp.mdl0<-setgee(fml0,i,wdtmp1); tmp.mdl1<-setgee(fml1,i,wdtmp1)

tmp.mdl2<-setgee(fml2,i,wdtmp1); tmp.mdlp<-setglm(fmlp,i,wdtmp1)

}

pd<-predict(tmp.mdlp,data.frame(xTMP=tp.value,tmp.X2=0), se.fit=TRUE)

prd<-paste(numfmt(pd$fit,dec)," (",numfmt(pd$fit-1.96*pd$se.fit,dec),", ", numfmt(pd$fit+1.96*pd$se.fit, dec),")",sep="")

m2<-mdl2oo(tmp.mdl2,"xTMP",opt)

m1<-mdl2oo(tmp.mdl1,c("tmp.X1","tmp.X2"),opt)

m0<-mdl2oo(tmp.mdl0,"tmp.X2",opt)

if (opt=="gee") {

plrt<-try(anova(tmp.mdl0,tmp.mdl2)$"P(>|Chi|)",TRUE)

plrt<-ifelse((plrt<="9" && plrt>="0"), pvformat(plrt,3),"-")

} else {plrt<- pvformat(1-pchisq(2*(logLik(tmp.mdl0)[1]-logLik(tmp.mdl2)[1]),1),3);}

oo<-list(c("",m2,"",tp.value,m1,m0,prd,plrt),tmpn)

return(oo)

}

get3lines<-function(i,j,g,tp.value,opt) {

if (is.na(g)) {fmladj1<-fmladj;wdtmp<-removeNA(i,j,WD);

} else if (g<nblv) {fmladj1<-fmladj;wdtmp0<-WD[WD[,bvar]==blv[g],];wdtmp<-removeNA(i,j,wdtmp0);

} else {fmladj1<-paste(fmladj,"+factor(",bvar,")",sep="");wdtmp<-removeNA(i,j,WD);}

xTMP<-wdtmp[,xvname[j]]; tp1<-tp.value[1]; tp2<-tp.value[2]

tmp.X1<- (xTMP< tp1)*(xTMP-tp1)

tmp.X2<-((xTMP>=tp1) & (xTMP<=tp2))*(xTMP-tp1)

tmp.X3<- (xTMP> tp2)*(xTMP-tp2)

tmp.B1<- (xTMP>tp2)

wdtmp1<-cbind(wdtmp,xTMP,tmp.X1,tmp.X2,tmp.X3,tmp.B1)

fml0<-paste(yvname[i],"~xTMP+tmp.X1+tmp.X3+tmp.B1",fmladj1)

fml1<-paste(yvname[i],"~tmp.X1+tmp.X2+tmp.X3+tmp.B1",fmladj1)

fml2<-paste(yvname[i],"~xTMP",fmladj1)

tmpn<-nrow(wdtmp)

if (opt=="glm") {

tmp.mdl0<-setglm(fml0,i,wdtmp1); tmp.mdl1<-setglm(fml1,i,wdtmp1); tmp.mdl2<-setglm(fml2,i,wdtmp1);

}

if (opt=="gam") {

tmp.mdl0<-setgam(fml0,i,wdtmp1); tmp.mdl1<-setgam(fml1,i,wdtmp1); tmp.mdl2<-setgam(fml2,i,wdtmp1);

}

if (opt=="gee") {

tmp.mdl0<-setgee(fml0,i,wdtmp1); tmp.mdl1<-setgee(fml1,i,wdtmp1); tmp.mdl2<-setgee(fml2,i,wdtmp1);

}

m2<-mdl2oo(tmp.mdl2,"xTMP",opt)

m1<-mdl2oo(tmp.mdl1,c("tmp.X1","tmp.X2","tmp.X3"),opt)

m0<-mdl2oo(tmp.mdl0,c("tmp.X1","tmp.X3"),opt)

if (opt=="gee") {

plrt<-try(anova(tmp.mdl0,tmp.mdl2)$"P(>|Chi|)",TRUE)

plrt<-ifelse((plrt<="9" && plrt>="0"), pvformat(plrt,3),"-")

} else {plrt<- pvformat(1-pchisq(2*(logLik(tmp.mdl0)[1]-logLik(tmp.mdl2)[1]),1),3);}

oo<-list(c("",m2,"",paste(tp.value,collapse=", "),m1,m0,plrt),tmpn)

return(oo)

}

getci4tp<-function(i,j,g,opt,tp0=NA) {

set.seed(123456)

if (is.na(g)) {wdt<-removeNA(i,j,WD);

} else if (g<nblv) {wdt<-WD[WD[,bvar]==blv[g],];wdt<-removeNA(i,j,wdt);

} else {wdt<-removeNA(i,j,WD);}

nnwd<-nrow(wdt); tp.vv<-rep(NA,1000)

if (!is.na(tp0)) {

tpp0 = sum(wdt[,xvname[j]] < tp0)/length(wdt[,xvname[j]])

tppmin = max(tpp0 - 0.09, 0.05)

tppmax = min(tpp0 + 0.09, 0.95)

} else {

tppmin = NA; tppmax = NA

}

for (s in (1:1000)) {

WDi<-wdt[sample(1:nnwd,nnwd,replace=T),]

tp.vv[s]<-get.tpval(i, j, NA, opt, WDi, tppmin, tppmax); rm(WDi)

}

tpci<-paste(quantile(tp.vv,probs=c(0.025,0.975)),collapse=", ")

return(tpci);

}

vlabelN<-(substr(vlabel,1,1)==" ");

vlabelZ<-vlabel[vlabelN];vlabelV<-vlabel[!vlabelN]

vnameV<-vname[!vlabelN];vnameZ<-vname[vlabelN];

allvname<-c(yvname,xvname,bvar,avname,subjvname,vname.start,vname.stop,timevname,"weights");

allvname<-allvname[!is.na(allvname)]

WD<-WD[,allvname];

w<-c("<html><head>","<meta http-equiv=\"Content-Type\" content=\"text/html\" charset=\"gb2312\" /></head><body>")

if (!is.na(avname[1])) {

if (sum((saf=="s" | saf=="S") & alv>0)>0) w<-c(w,"</br>Spline smoothing only applies for continuous variables")

if (!is.na(subjvname)) saf<-rep(0,length(saf))

}

if (sum(xlv>0)>0) w<-c(w,"Categorical exposure variables were ignored")

xvname<-xvname[xlv==0];

if (!is.na(subjvname)) WD<-WD[order(WD[,subjvname]),];

fmladj<-""; avb=""; smoothav<-0;

if (!is.na(avname[1])) {

avb<-vlabelV[match(avname,vnameV)];

avname_ <- avname

smoothavi<-((saf=="s" | saf=="S") & alv==0)

smoothav<-sum(smoothavi)

avname_[smoothavi]<-paste("s(",avname[smoothavi],")",sep="")

avb1<-avb

avb1[smoothavi]<-paste(avb[smoothavi],"(Smooth)",sep="")

avname_[alv>0]<-paste("factor(",avname[alv>0],")",sep="")

fmladj<-paste("+",paste(avname_,collapse="+"))

}

if (is.na(bvar)) {

blvb<-"N"; blvb_<-"N"; nblv<-1; blbl<-"";

} else {

blbl<-vlabelV[match(bvar,vnameV)]; if (is.na(blbl)) blbl<-bvar;

blv<-levels(factor(WD[,bvar])); nblv<-length(blv)+1

blvb_<-vlabelZ[match(paste(bvar,".",blv,sep=""),vnameZ)];

blvb_[is.na(blvb_)]<-blv[is.na(blvb_)];

blvb<-c(paste(blbl,blvb_,sep="="),"Total");

blvb_<-c(blvb_,"Total")

WD<-WD[!is.na(WD[,bvar]),]

}

ny=length(yvname); nx=length(xvname);

xb<-vlabelV[match(xvname,vnameV)]; xb[is.na(xb)]<-xvname[is.na(xb)]

yb<-vlabelV[match(yvname,vnameV)]; yb[is.na(yb)]<-yvname[is.na(yb)]

opt<-ifelse(!is.na(subjvname), "gee", ifelse(smoothav>0, "gam", "glm")) ;

colprn<-parm[3]

if (is.na(par1)) par1<-"";

if (is.numeric(par1)) {tp.vv<-par1;

} else {

tmp<-as.numeric(strsplit(par1," ")[[1]]); tp.vv<-c(tmp[!is.na(tmp)],NA)

}

prn<-ifelse(!is.na(bvar), "S", ifelse(nx>1 & ny==1, "X", "Y"));

if (length(tp.vv)>2) tp.vv<-tp.vv[1:2]

ntp<-length(tp.vv);

getci<-FALSE

prnopt<-c("β (95%CI) Pvalue / OR (95%CI) Pvalue", "β (95%CI) Pvalue", "β+se / OR (95%CI) *P<0.05 **P<0.01 ***P<0.001")

if (ntp==1) {

cc0<-c("模型 I","&nbsp&nbsp一条直线效应");

cc0<-c(cc0,"模型 II","&nbsp&nbsp折点(K)","&nbsp&nbsp &lt K 段效应 1","&nbsp&nbsp &gt K 段效应 2","&nbsp&nbsp 2与1的效应差")

cc0<-c(cc0,"&nbsp&nbsp折点处方程预测值")

if (is.na(tp.vv[1]) & !is.na(parm[1])) getci<-TRUE;

} else {

cc0<-c("模型 I","&nbsp&nbsp一条直线效应");

cc0<-c(cc0,"模型 II","&nbsp&nbsp折点(K1,K2)","&nbsp&nbsp &lt K1 段效应 1","&nbsp&nbsp K1-K2 段效应 2","&nbsp&nbsp &gt K2 段效应 3")

cc0<-c(cc0,"&nbsp&nbsp 1与2的效应差","&nbsp&nbsp 3与2的效应差")

}

if (opt=="gee") {cc0<-c(cc0,"ANOVA 两模型比较");} else {cc0<-c(cc0,"对数似然比检验");}

if (getci) cc0<-c(cc0,"折点的95可信区间");

sink(paste(ofname,".lst",sep=""))

w<-c(w,paste("<h2>", title, "</h2>"))

nn<-c("Outcome","Exposure",blvb);

if (prn=="Y") {

for (j in 1:nx) {

tt<-cc0;

for (i in 1:ny) {

if (is.na(tp.vv[1])) {tp.v<-get.tpval(i,j,NA,opt,NA);} else {tp.v<-tp.vv;}

if (ntp==1) tmpij<-get2lines(i,j,NA,tp.v,opt);

if (ntp==2) tmpij<-get3lines(i,j,NA,tp.v,opt);

if (getci) {tt<-cbind(tt,c(tmpij[[1]],getci4tp(i,j,NA,opt,tp.v)));} else {tt<-cbind(tt,tmpij[[1]]);}

nn<-rbind(nn,c(yb[i],xb[j],tmpij[[2]]))

}

tt<-rbind(c("Outcome: ",yb),tt)

w<-c(w,paste("</br>For exposure:",xb[j]))

w<-c(w,"</br><table border=3>", mat2htmltable(tt), "</table>")

}

}

if (prn=="X") {

for (i in 1:ny) {

tt<-cc0;

for (j in 1:nx) {

if (is.na(tp.vv[1])) {tp.v<-get.tpval(i,j,NA,opt,NA);} else {tp.v<-tp.vv;}

if (ntp==1) tmpij<-get2lines(i,j,NA,tp.v,opt);

if (ntp==2) tmpij<-get3lines(i,j,NA,tp.v,opt);

if (getci) {tt<-cbind(tt,c(tmpij[[1]],getci4tp(i,j,NA,opt,tp.v)));} else {tt<-cbind(tt,tmpij[[1]]);}

nn<-rbind(nn,c(yb[i],xb[j],tmpij[[2]]))

}

tt<-rbind(c("Exposure: ",xb),tt)

w<-c(w,paste("</br>For outcome:",yb[i]))

w<-c(w,"</br><table border=3>", mat2htmltable(tt), "</table>")

}

}

if (prn=="S") {

for (i in 1:ny) {

tt<-cc0;

for (j in 1:nx) {

nnij<-c(yb[i],xb[j])

for (g in 1:nblv) {

if (is.na(tp.vv[1])) {tp.v<-get.tpval(i,j,g,opt,NA);} else {tp.v<-tp.vv;}

if (ntp==1) tmpij<-get2lines(i,j,g,tp.v,opt);

if (ntp==2) tmpij<-get3lines(i,j,g,tp.v,opt);

if (getci) {tt<-cbind(tt,c(tmpij[[1]],getci4tp(i,j,g,opt,tp.v)));} else {tt<-cbind(tt,tmpij[[1]]);}

nnij<-c(nnij,tmpij[[2]])

}

nn<-rbind(nn,nnij)

}

tt<-rbind(c(blbl,blvb_),tt)

w<-c(w,paste("</br>For outcome:",yb[i]))

w<-c(w,paste("</br>For Exposure:",xb[j]))

w<-c(w,"</br><table border=3>", mat2htmltable(tt), "</table>")

}

}
